# T cell receptor repertoire sequencing reveals chemotherapy-driven clonal expansion in colorectal liver metastases

**DOI:** 10.1093/gigascience/giad032

**Published:** 2023-05-10

**Authors:** Eirik Høye, Vegar J Dagenborg, Annette Torgunrud, Christin Lund-Andersen, Åsmund A Fretland, Susanne Lorenz, Bjørn Edwin, Eivind Hovig, Bastian Fromm, Else M Inderberg, Victor Greiff, Anne H Ree, Kjersti Flatmark

**Affiliations:** Department of Tumor Biology, Institute for Cancer Research, The Norwegian Radium Hospital, Oslo University Hospital, 0379 Oslo, Norway; Institute of Clinical Medicine, Medical Faculty, University of Oslo, 0318 Oslo, Norway; Department of Tumor Biology, Institute for Cancer Research, The Norwegian Radium Hospital, Oslo University Hospital, 0379 Oslo, Norway; Department of Gastroenterological Surgery, The Norwegian Radium Hospital, 0379 Oslo, Norway; Department of Tumor Biology, Institute for Cancer Research, The Norwegian Radium Hospital, Oslo University Hospital, 0379 Oslo, Norway; Department of Tumor Biology, Institute for Cancer Research, The Norwegian Radium Hospital, Oslo University Hospital, 0379 Oslo, Norway; Institute of Clinical Medicine, Medical Faculty, University of Oslo, 0318 Oslo, Norway; The Intervention Centre, Rikshospitalet, Oslo University Hospital, 0372 Oslo, Norway; Department of Hepato-Pancreato-Biliary Surgery, Rikshospitalet, Oslo University Hospital, 0372 Oslo, Norway; Department of Core Facilities, Institute for Cancer Research, The Norwegian Radium Hospital, Oslo University Hospital, 0379 Oslo, Norway; Institute of Clinical Medicine, Medical Faculty, University of Oslo, 0318 Oslo, Norway; The Intervention Centre, Rikshospitalet, Oslo University Hospital, 0372 Oslo, Norway; Department of Hepato-Pancreato-Biliary Surgery, Rikshospitalet, Oslo University Hospital, 0372 Oslo, Norway; Center for Bioinformatics, Department of Informatics, University of Oslo, 0316 Oslo, Norway; The Arctic University Museum of Norway, UiT – The Arctic University of Norway, 9037 Tromsø, Norway; Translational Research Unit, Department of Cellular Therapy, Oslo University Hospital, 0379 Oslo, Norway; Department of Immunology, University of Oslo and Oslo University Hospital, 0372 Oslo, Norway; Institute of Clinical Medicine, Medical Faculty, University of Oslo, 0318 Oslo, Norway; Department of Oncology, Akershus University Hospital, 1478 Lørenskog, Norway; Department of Tumor Biology, Institute for Cancer Research, The Norwegian Radium Hospital, Oslo University Hospital, 0379 Oslo, Norway; Institute of Clinical Medicine, Medical Faculty, University of Oslo, 0318 Oslo, Norway; Department of Gastroenterological Surgery, The Norwegian Radium Hospital, 0379 Oslo, Norway

**Keywords:** colorectal cancer, T-cell receptor sequencing, liver metastasis, neoadjuvant chemotherapy, clonal expansion, clinical samples

## Abstract

**Background:**

Colorectal liver metastasis (CLM) is a leading cause of colorectal cancer mortality, and the response to immune checkpoint inhibition (ICI) in microsatellite-stable CRC has been disappointing. Administration of cytotoxic chemotherapy may cause increased density of tumor-infiltrating T cells, which has been associated with improved response to ICI. This study aimed to quantify and characterize T-cell infiltration in CLM using T-cell receptor (TCR) repertoire sequencing. Eighty-five resected CLMs from patients included in the Oslo CoMet study were subjected to TCR repertoire sequencing. Thirty-five and 15 patients had received neoadjuvant chemotherapy (NACT) within a short or long interval, respectively, prior to resection, while 35 patients had not been exposed to NACT. T-cell fractions were calculated, repertoire clonality was analyzed based on Hill evenness curves, and TCR sequence convergence was assessed using network analysis.

**Results:**

Increased T-cell fractions (10.6% vs. 6.3%) were detected in CLMs exposed to NACT within a short interval prior to resection, while modestly increased clonality was observed in NACT-exposed tumors independently of the timing of NACT administration and surgery. While private clones made up >90% of detected clones, network connectivity analysis revealed that public clones contributed the majority of TCR sequence convergence.

**Conclusions:**

TCR repertoire sequencing can be used to quantify T-cell infiltration and clonality in clinical samples. This study provides evidence to support chemotherapy-driven T-cell clonal expansion in CLM in a clinical context.

## Introduction

Increased understanding of how the immune system is involved in cancer development and progression has resulted in development of therapeutic interventions successfully targeting the immune system, such as the immune checkpoint inhibitors (ICIs) targeting the PD-1/PD-L1 axis. Mismatch repair–deficient cancers with high tumor mutational burden have been shown to respond strongly to ICIs regardless of histologic type [1], which has led to interest in identification of specific tumor neoantigens. Characterization of tumor antigen-specific T cells has therefore become an important step to further understand antitumor immunity. The noncoding part of the genome is increasingly perceived as a major contributor to tumor neoantigens, as up to 70% of the genome is transcribed in some form [2]. Abnormally expressed RNA in tumor tissue could therefore contribute as neoantigens in cancers with modest tumor mutational burden. T cells recognize their cognate antigens through interaction with the peptide-major histocompatibility complex Major histocompatibility complex (MHC) presented on the surface of target cells via the complementarity determining region 3 (CDR3) of the T-cell receptor (TCR). Recent advances in deep sequencing of the TCR CDR3 region have enabled quantification of T-cell clones with the same antigen specificity and characterization of TCR repertoires across biological compartments and over time [3, 4]. Applying this technology to metastatic tumor samples represents an important opportunity to further understand T-cell immunity in metastatic cancer and possibly identify neoantigens associated with immune responses.

Colorectal cancer (CRC) accounts for about 10% of all diagnosed cancers and cancer-related deaths worldwide, and colorectal liver metastasis (CLM) is a leading cause of CRC-related mortality [5]. Surgery is a curative treatment option for a minority of patients with limited metastatic disease, but for most patients with CLM, systemic chemotherapy is the main treatment option, and the survival rates are poor [6]. Durable responses to ICIs have been observed in patients with CRC with microsatellite-unstable tumors, but with the majority of patients having microsatellite-stable (MSS) cancers, responses are generally disappointing [7]. Still, in MSS CRC, there is evidence to suggest that the microenvironmental immune contexture is important, such as in primary CRC, where a high density of tumor-infiltrating T cells was shown to strongly correlate with a favorable long-term outcome [8]. In previous studies of CLM, we observed upregulation of immune-related genes and increased T-cell intratumoral densities after exposure to neoadjuvant chemotherapy (NACT) [9, 10]. This suggests that NACT can modify the CLM immune microenvironment, possibly by induction of immunogenic cell death, toward a state that could potentially be more responsive to ICIs.

In this work, we have sequenced TCR repertoires in resected CLMs from 85 patients included in the Oslo Randomized Laparoscopic Versus Open Liver Resection for Colorectal Metastases Study (OSLO-COMET) study, and repertoires were compared according to NACT exposure to analyze T-cell fractions and clonality. In addition, network analysis was used to assess sequential convergence reported to be associated with antigen-experienced repertoires.

## Methods

### Patient samples

**Figure 1:**
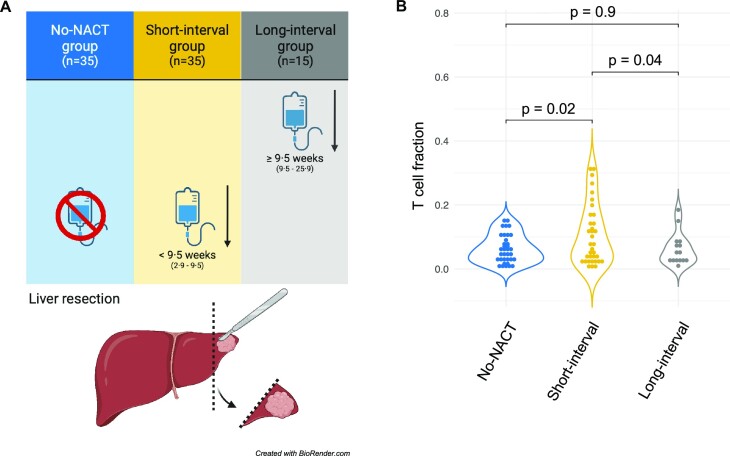
Patient cohort and T-cell fractions according to neoadjuvant chemotherapy (NACT) administration groups. (A) Overview of NACT administration groups. We previously determined the NACT administration cutoff level to be 9.5 weeks based on receiver operator curve analysis [9]: the no-NACT group did not receive chemotherapy prior to liver resection, while the short- and long-interval groups received NACT 2.9 to <9.5 and ≥9.5 to 25.9 weeks prior to liver resection, respectively. (B) The mean T-cell fraction was higher in the short-interval group than in the no-NACT group. *P* values are from 2-sided Welch *t*-test.

CLM samples were collected from 85 patients included in the OSLO-COMET study (NCT01516710) (for clinical data, see Table 1). Written informed consent was obtained from all participating patients. The OSLO-COMET study was approved by Norway's Regional Committees for Medical and Health Research Ethics (ID# 2011/1285/REK sør-øst B). NACT was administered to 50 patients (59%), while 35 patients (41%) did not receive NACT (no-NACT group) (Fig. 1A). The NACT regimen, the timing of liver resection after NACT, and the number of NACT cycles were not predefined by the OSLO-COMET study protocol but were decided for the individual patient by a multidisciplinary team [9]. We previously determined that a 9.5-week interval between completion of NACT and liver resection was a cutoff for observing an increase of intratumoral T-cell density [9]. Applying the cutoff to this cohort, 35 (41%) and 15 (18%) patients had received NACT less than and more than 9.5 weeks prior to liver resection, respectively (hereafter termed the short-interval [median 7, min–max 2.9–9.5 weeks] and long-interval [median 16, min–max 9.5–25.9 weeks] groups). Tumor samples were fresh frozen in liquid nitrogen and stored at −80˚C. The tumor content was evaluated by the study pathologist and the tissues were processed and homogenized as previously described [9]. Briefly, DNA was isolated using the Allprep DNA/RNA/miRNA Universal Kit (Qiagen, Düsseldorf, Germany; cat. 80224), and DNA purity was determined using the Nanodrop 2000 spectrophotometer (Thermo Fisher, Waltham, MA, USA). Aliquots were diluted to 200 ng/µL, as measured with the QuBit dsDNA Broad Range Assay Kit (Thermo Fisher; cat. Q32850). Three of the 85 patients included in the study had 2 TCR repertoire datasets from the same metastasis but different tissue aliquot, representing technical replicates. For 4 patients, TCR repertoire datasets were derived from 2 separate metastases resected at the same procedure, providing information about potential heterogeneity of TCR repertoires in metastases located in the same liver.

**Table 1:** Clinical data for cohort used in this study.

Variable	Group	*N* (%)	Median (min–max)
Gender	Male	44 (52)	
	Female	41 (48)	
Age; years			65 (57–70)
Microsatellite-stable cases		85 (100)	
Primary tumor location			
	Right colon	17 (20)	
	Left colon	30 (35)	
	Rectum	35 (41)	
Overall survival (from CLM surgery), months			56 (37–79)
NACT	No	35 (41)	
	Yes	50 (59)	
			
Interval NACT and CLM surgery, weeks	Short-interval group	35	7 (2.9–9.5)
	Long-interval group	15	16 (9.5–25.9)

### T-cell receptor sequencing

TCR repertoire sequencing libraries were prepared using the hsTCRB v3 immunoSEQ library preparation kit (Adaptive Biotechnologies, Seattle, WA, USA). Briefly, two 16-µL polymerase chain reaction (PCR) replicates were prepared for each biological sample, with genomic DNA (gDNA) content within the range recommended by the manufacturer for nonlymphoid tissues, which is predicted to yield the optimal 30,000 to 45,000 T cells per PCR replicate. The library preparation protocol uses multiplex PCR with primers for all possible V and J fragments in the TCR-β gene with adjustment of primer concentrations to account for variable primer efficiencies. Synthetic repertoires with known quantities are present in each PCR reaction to allow accurate quantification of T cells in each sample [11]. Pooled libraries were sequenced using the NextSeq 500/550 Mid Output kit v2.5 (150 cycles) (Illumina, San Diego, CA, USA). Bioinformatics processing of raw data was done by Adaptive Biotechnologies proprietary analysis pipeline, which provided data on the immunoSEQ rearrangement-level file format. The T-cell fraction was determined by dividing the sum of detected rearranged DNA templates (1 rearranged DNA template as a proxy for 1 T cell) with the total number of genomes in the sample, which was calculated by the total amount of DNA/6.6 pg (mean DNA content/cell). T cells with identical CDR3β sequence and length were defined as 1 clonotype for subsequent analyses. As previously described, a clone was considered “private” if detected in only 1 patient, with “public” clones being present in more than 1 patient [12], and the sharing level was defined as the number of patients in whom a specific clonotype was detected.

### TCR repertoire clonality using Hill diversity and evenness profiles

The alakazam v1.1.0 R package [13] was used to generate Hill diversity and evenness profiles for each dataset, as previously described [14]. The range of *q* parameters was set between *q* = 0 and *q* = 10, with steps of 0.2. Evenness profiles are defined by the equation ^α^*E =*  ^α^*D/SR* (or the Hill diversity profile divided by Hill diversity at *q* = 0), as previously described in [15]. For each repertoire, a clonality index, defined as 10 minus the area under the curve (AUC), was calculated for individual evenness curves using the sintegral() function from the Bolstad2 v1.0–28 R package [16], which gives a parameter ranging from 0 to 10, with higher values signifying more oligoclonal repertoires.

### Analysis of within-repertoire TCR sequence similarity using Levenshtein distance networks

To analyze TCR sequence similarity within T-cell repertoires, the ImNet v0.2.1 package was used to generate Levenshtein distance (LD) [17] matrices for all unique CDR3β sequences in each dataset. These distance matrices were then used to construct CDR3β sequential convergence networks, where connections were built between CDR3β sequences at LD = 1. Visualization of the networks was accomplished with Cytoscape v3.9.0 [18]. The resulting networks illustrate the overall similarity of CDR3β sequences within a T-cell repertoire and the presence of subclusters of CDR3β sequences. Global parameters calculated for each network included the number of nodes (unique clones) and number of edges (clonal connections), and the network connectivity fraction was calculated by dividing edges with nodes. The powerlaw() R package [19] was used to assess whether the network degree distribution followed a power law function. A goodness-of-fit test was conducted, where the null hypothesis was power law distribution. X-min value was determined for each distribution. A degree distribution with a power law goodness-of-fit *P* value greater than 0.1 was considered a plausible power law distribution. Local parameters to characterize individual clones included the number of degrees (connections between clones in a network) and the sharing level (number of patients where a clone was detected). As was done previously in [12, 20], to compare network connectivity of public and private clones, clones from all repertoires were classified as public or private, subsampled to 1,000 clones to compensate for the numerical overrepresentation of private clones, and networks were generated with the same method as above.

To further explore potential associations between TCR connectivity, sharing level, clonal expansion, and potential disease pathologies, the McPAS database [21] was used. The database was downloaded on 10 May 2022 and is available in the code repository. Two groups of TCRs were defined, one consisting of top 10% of clones with highest connectivity and/or sharing level and the other consisting of the top 10% most clonally expanded TCRs. The fraction of TCRs that matched at LD = 0 with known pathology-associated TCRs in McPAS was calculated for each sample.

### Analysis of overlapping clones between repertoires

The Morisita–Horn index (MHI) was used to assess clonal similarity between repertoires. This index shows the overall clonal overlap between 2 repertoires, weighted by clonal frequency, ranging from 0 (no overlap) to 1 (complete overlap). A combined rearrangement file was downloaded from the immunoSEQ Analyzer. This file format has all clones across all datasets for each row and the count of that clonotype in each dataset for columns. This was input to the divo R package mh() v1.0.1 function, which yielded pairwise MHI comparison for all datasets. Pairwise comparisons were made between repertoires from all analyzed patients; in addition, comparisons were made between repertoires generated from different aliquots from the same metastasis (*n* = 3) and repertoires from different metastases in the same patient (*n* = 4).

### Statistical analyses

For T-cell fraction analysis, 1 dataset per patient was included (*n* = 85), while for subsequent analyses, in order to avoid bias due to low-frequency clones, 8 datasets with sequencing coverage <5 were excluded. Total number of samples available for downstream analysis was 77 (No-NACT = 32, short-NACT = 30, long-NACT = 15). T-cell fractions and clonality were described using mean and 95% confidence intervals (CIs). Mean T-cell fractions and clonality in the NACT administration groups were compared using the Welch *t*-test using the compare_means() function from ggpubr v0.4.0. Comparison of clonality against the location of the primary colorectal cancer (pCRC) was also made. Linear regression was used to analyze the relationship between clonality and the number of T cells, as well as the network connectivity fraction and number of clones, using the lm() function in base R v4.0.5. Associations between clonality and primary tumor location (right colon, left colon, or rectum) were also analyzed using the Welch *t*-test using the compare_means() function from ggpubr (RRID:SCR_021139) v0.4.0. Overall survival was measured from the time of CLM resection. The last liver resection date was on 28 January 2016, and the censoring date was on 8 January 2020. The Kaplan–Meier method was used to estimate patient survival, while the log-rank test was used to see if there was a difference between survival curves of patients with low, medium, or high clonality, using the survival (RRID:SCR_021137) v3.2–12 package. *P* values <0.05 were considered to indicate statistical significance.

## Results

### High T-cell fraction was associated with a short interval between NACT administration and surgery

The mean number of productive CDR3β DNA templates per sample was 83,098, with 95% CI (66,615–99,582). When normalized against the total amount of gDNA, the mean T-cell fraction was 8.1%, with 95% CI (6.5%–9.6%). The mean T-cell fraction was higher in the short-interval group (10.6% [7.4–13.8%]) compared to the no-NACT group (6.3% [4.8–7.9%]), *P* = 0.02 (Fig. 1B). The T-cell fraction in the long-interval group (6.2% [3.4–8.9%]) was also significantly lower compared to the short-interval group (*P* = 0.04), while there was no difference compared to the no-NACT group.

### NACT exposure was associated with more clonal TCR repertoires

**Figure 2:**
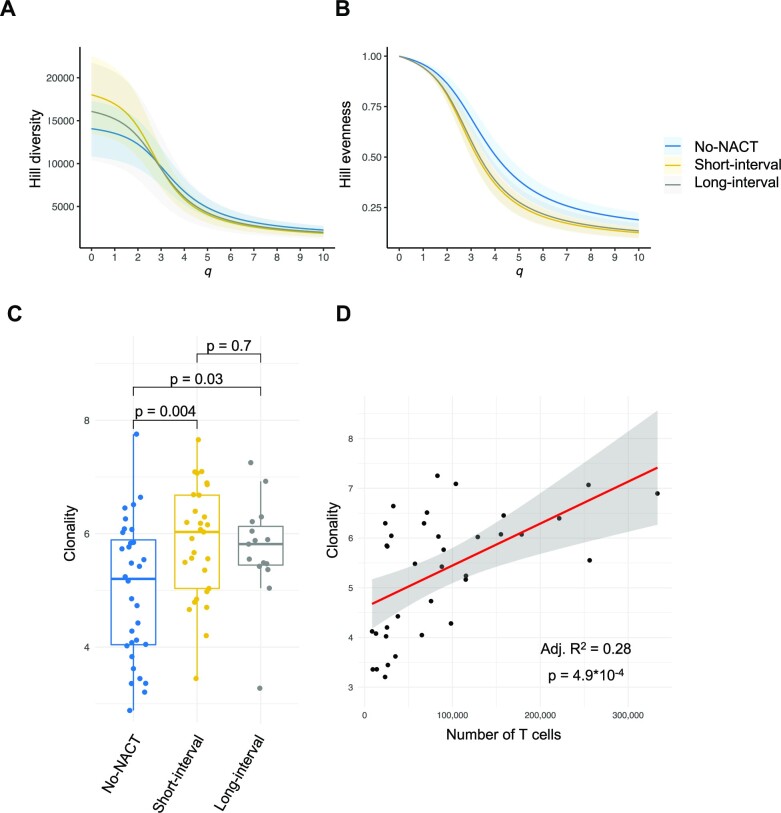
Hill diversity and evenness profiles according to neoadjuvant chemotherapy (NACT) administration group. (A) Hill diversity profiles for 77 individual datasets by mean (solid line) with 95% confidence interval (shaded area), with *q* values between 0 and 10. The left part of each curve (*q* = 0) represents the number of unique clones present in the repertoire. The further and more rapidly each curve drops at increasing values of *q*, the more uneven the clonal frequency distribution (more clonal). (B) Hill evenness profiles illustrated by mean (solid line) with 95% confidence interval (shaded area). Evenness profiles are diversity profiles normalized by the number of unique clones present in the repertoire. (C) Boxplot of TCR repertoire clonality, defined by 10 – AUC of evenness profile. The middle bar denotes median clonality. Box represents first and third quartiles, while whiskers represent minimum and maximum values. *P* -values are from a 2-sided Welch *t*-test. (D) Linear regression of T-cell clonality, and the number of T cells (red line) shows a modest correlation between clonality and a high number of intratumoral T cells. Shaded area is the 95% confidence interval of the linear regression intercept and slope. Number of T cells is determined from the total number of rearranged DNA templates detected by sequencing.

In total, 1,413,435 unique clones were identified across all datasets, median 15,596 (min–max, 1,476–66,976). Hill diversity profiles (Fig. 2A) showed considerable variability at the species richness range (*q* = 0) (mean 16,000, 95% CI [13,611–18,388]). Both the short-interval (18,020 [13,524–22,515]) and long-interval groups (16,079 [10,394–21,764]) exhibited nonsignificant trends toward a higher number of unique clones compared to the no-NACT group (14,069 [10,833–17,304]). At increasing values of *q*, the mean in the short- and long-interval groups intersected and fell below the mean of the no-NACT group but with overlapping CIs. When comparing the Hill evenness profiles (Fig. 2B), which are diversity profiles normalized against the number of unique clones (also called species richness), the short- and long-interval groups had very similar means, and the curve for both groups was lower than for the no-NACT group, with nonoverlapping 95% CI. Comparison of clonality based on AUC calculations from individual evenness curves showed that the short-interval (5.8 [5.5–6.2]) and long-interval (5.7 [5.2–6.2]) groups had higher mean values than the no-NACT group (5.0 [4.6–5.4]) (*P* = 0.004 and *P* = 0.03, respectively) (Fig. 2C). Furthermore, regression analysis revealed a modest association between a high absolute number of T cells and clonality (Fig. 2D). There were no associations between clonality and overall survival (Supplementary Fig. S1), but a nonsignificant trend toward increased clonality was observed when comparing samples from right-sided with left-sided primary tumors (*P* = 0.09, Supplementary Fig. S2).

### T-cell similarity networks clustered around publicly conserved subsequences

**Figure 3:**
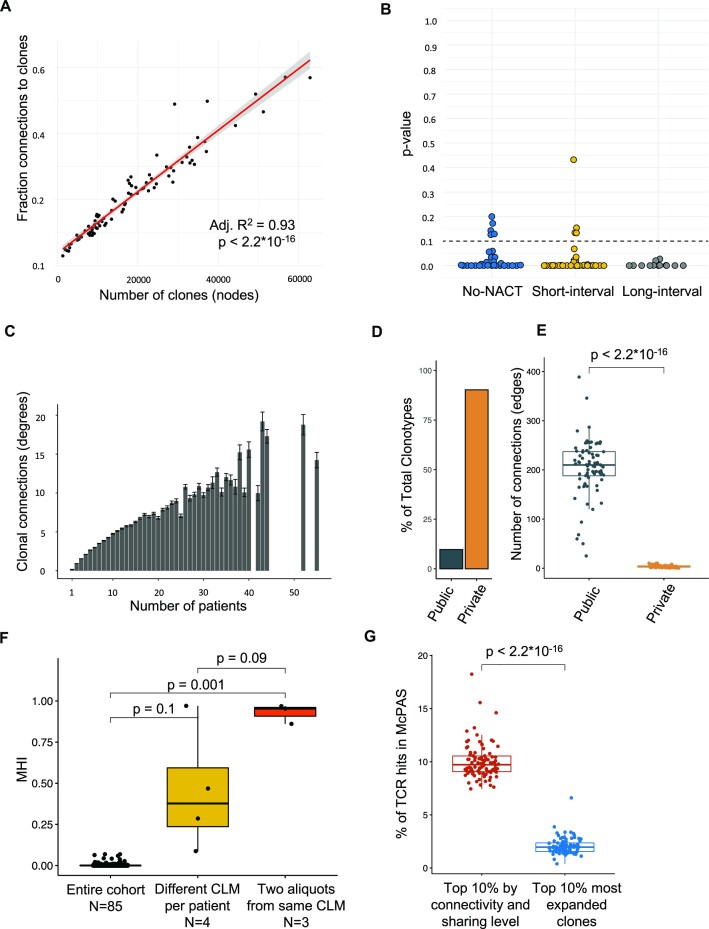
Network analyses. (A) The network connectivity fraction (number of edges to number of nodes) plotted against the number of nodes. A linear regression model was fitted to the data (red line), and the equation parameters are shown top left. The connectivity of the networks increased exponentially with the network size. Shaded area is the 95% confidence interval of the linear regression intercept and slope. (B) Plot of *P* values from goodness-of-fit test to a power law function of TCR repertoire network degree distributions. A *P* value greater than 0.1 was considered to indicate power law distribution. (C). Barplot of the number of clonal connections (degrees) against the number of patients in which a clone was detected. Error bars represent 95% confidence interval. (D) Barplot of the percentage of public versus private clones across all datasets. (E) Comparison of network connectivity generated from TCR repertoires stratified into public and private clones, randomly subsampled to 1,000 clones per network (because of the low abundance of public clones). The middle bar denotes the median, while the box represents the first and third quartiles, and whiskers represent minimum and maximum values (values outside this range reflect outliers). (F) TCR repertoire similarity assessed using the Morisita–Horn index (MHI). The MHI gives an estimate of the clonal similarity between 2 repertoires, weighted by clonal frequency, ranging from 0 (no similarity) to 1 (identical). The figure shows TCR repertoire similarity for the entire cohort, for datasets from different CLMs from the same patient (*n* = 4) and for datasets from analysis of 2 tissue aliquots from the same CLM (*n* = 3). The middle bar denotes the median, while the box represents the first and third quartiles, and whiskers represent minimum and maximum values. (G) Fraction of TCRs that were detected in the McPAS pathology-associated TCR database. For each repertoire, TCR clones were stratified into 2 groups: the top 10% most highly connected (degree) and public (sharing level) clones and the top 10% most expanded clones. The fraction was calculated from the number of clones with McPAS hits at a Levenshtein distance of 0 in each group, divided by total number of clones among the top 10% in each group. The middle bar denotes the median, while the box represents the first and third quartiles, and whiskers represent minimum and maximum values (values outside this range reflect outliers).

Networks were generated based on the LD of TCR sequences for each repertoire. The purpose of this analysis was to see if there was a tendency of TCR sequence convergence, which might indicate shared specificity to common antigen epitopes. The mean number of nodes detected per repertoire was 16,056 (95% CI, 13,637–18,475), while the mean number of edges was 4,133 (3,015–5,251), generating a mean network connectivity fraction of 19.0% (16.5%–21.5%). The connectivity fraction increased linearly as a function of the total number of clones, from less than 5% in small networks to greater than 50% in large networks (Fig. 3A).

This observation suggests that the network connectivity fraction was not associated with the clonality of the T-cell repertoire frequency distribution. Furthermore, prior studies on antibody repertoires reported that the clonal degree distribution of LD-based networks resembles a power law distribution, whereas naive networks do not [17, 22]. While the majority of the networks in the context of T-cell repertoires in this study did not follow a power law distribution (Fig. 3B), 4 short-interval and 5 no-NACT repertoires passed the power law fit test. The mean clonality for the networks that passed the test was 4.4, which was lower than the mean clonality across all datasets of 5.5. Clonally expanded datasets were therefore also not associated with power law degree distributions, suggesting that T-cell repertoires follow different dynamics compared to B-cell repertoires.

Instead, connectivity was associated with public clonal sharing level, where the number of degrees increased linearly with increasing sharing level (Fig. 3C). The majority of the detected clones were private (90.3%), while 9.7% of the clones were public. (Fig. 3D). The public clones exhibited a much higher level of connectivity than the private clones, with a mean connectivity fraction of 20.5% (19.2%–21.8%) compared to 0.4% (0.3%–0.4%), respectively (Fig. 3E). This finding is further illuminated by the MHI comparisons (Fig. 3F), where repertoires from different patients had very low overlap of mean 0.0007 (min–max, 0–0.07). MHI calculated from repertoires generated from different aliquots from the same metastasis was high, with a mean of 0.93 (min–max, 0.86–0.97), while repertoires from different metastases in the same patient exhibited moderate overlap, with a mean MHI of 0.5 (min–max, 0.09–0.97). The TCR clones with highest connectivity and/or sharing level were more commonly associated with known pathogens (10% of clones), according to the McPAS database, compared with the most expanded TCR clones (3% of clones) (Fig. 3G). Interestingly, the 5 most common pathologies detected were influenza, tuberculosis, colorectal cancer, cytomegalovirus, and Epstein–Barr virus (see Supplementary Table S1).

**Figure 4:**
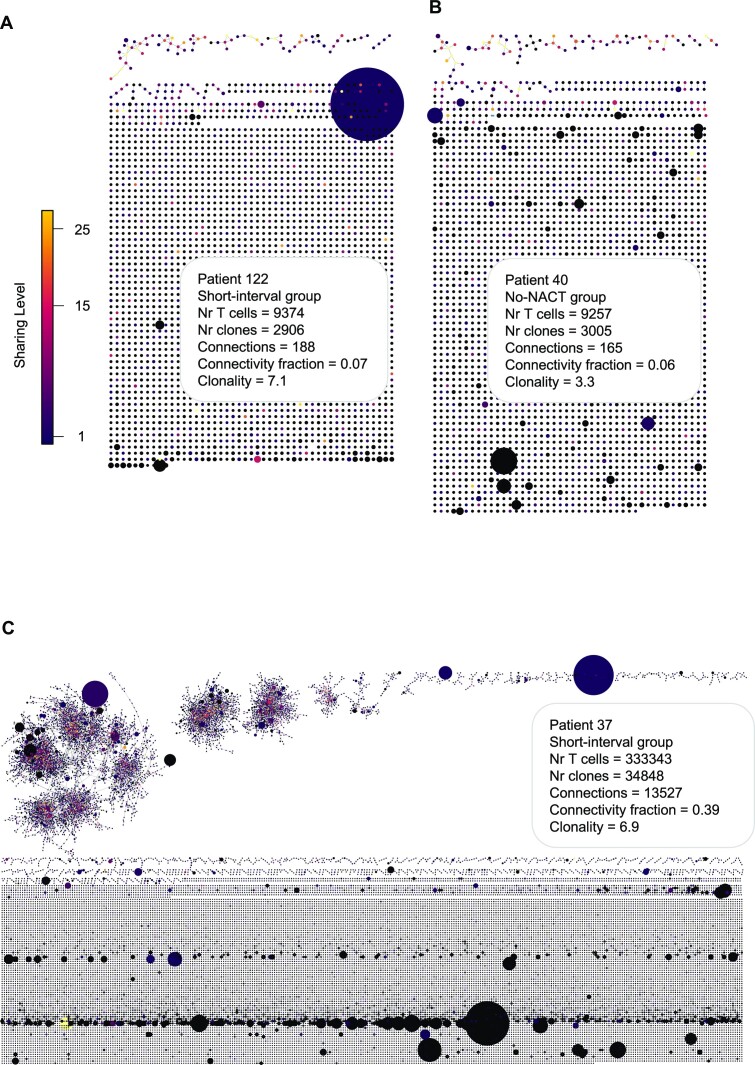
Visualization of representative CDR3β sequence connectivity networks. Each node (dot) corresponds to a unique T-cell CDR3β amino acid sequence, while connections are made between nodes when LD = 1. The size of each node corresponds to its relative clonal abundance, while the color scale represents the sharing level of the clone; dark blue represents clones unique to the individual patient, while yellow represents clones present across a large number of patients. (A, B) Networks selected from the short-interval (A) and no-NACT (B) groups. The 2 networks exhibit similar TCR parameters with respect to numbers of T cells and unique clones, as well as number of connections and connectivity fraction, but exemplify opposite ends of the clonality spectrum. (C) A network from the short-interval group exemplifying a network with a very high number of T cells and unique clones.

Three representative networks are shown in Fig. 4. A network from the short-interval group in the highly clonal end of the spectrum (clonality = 7.1) had a connectivity profile with 2,907 clonotypes, 188 connections, and a network connectivity fraction of 7% (sample 122; Fig. 4A). The most expanded clone (2,053 T cells) was not detected in any other samples, representing a private clone. A more heterogeneous, lowly clonal network (clonality = 3.3) from the no-NACT group had a very similar connectivity profile, exhibiting 3,005 clones, 165 connections, and a network connectivity fraction of 6% (sample 40; Fig. 4B). Finally, a very large, highly clonal network (clonality = 6.9) was visualized, with 34,849 clones and 13,527 connections, and a high network connectivity fraction of 39% (sample 37; Fig. 4C). Again, the majority of expanded clones were private to this sample.

## Discussion

In this work, using TCR sequencing, we found that the T-cell fraction was significantly higher in tumors with a short interval between NACT exposure and liver resection compared to tumors not exposed to NACT. This finding is in line with previously published work from our group, showing higher T-cell density in the short-interval group compared to the no-NACT and long-interval groups [9]. Considering that different parts of the tumors were analyzed (whole-section immunohistochemistry vs. snap-frozen tissue from the surgical samples) and that different methods were used to quantify T cells, the concordance is remarkable.

Although a significant increase of infiltrating T cells was detected in the short-interval group only, analysis of Hill diversity curves revealed that both the short- and long-interval groups had more uneven clonal frequency distributions compared to the no-NACT group. As previously explained by Hill [14], the diversity at *q* = 0 corresponds to the total number of clones in the repertoire (clonal richness). Because clonal richness is influenced by sampling depth and the presence of rare clones, the frequencies should also be assessed across a range of diversity parameters [14, 15], rather than by commonly used single-point estimates, to obtain a complete picture of clonal frequency distributions. At *q* values greater than 2 (Simpson index [14]), the influence of rare clones on the Hill diversity estimate becomes negligible and is instead influenced by abundant clones. For our datasets, the short-interval group diversity profile was higher at *q* = 0, corresponding with the higher T-cell fraction observed in this group. Yet, it intersected and became similar to the no-NACT profile at higher values of *q*, indicating that the higher clonal richness was driven by rare clones, likely reflecting the higher T-cell fraction in this group. However, the slope of both short-interval and long-interval profiles was steeper than the no-NACT profile, still suggesting qualitative differences in the clonal frequency distribution that could be related to NACT exposure. Hill diversity estimates cannot be used to quantitatively compare the clonality of repertoires if the number of sampled T cells is very different. Instead, Hill evenness profiles, which normalize datasets by clonal richness, will provide a more correct comparison of clonality between repertoires. We therefore calculated AUC values from Hill evenness profiles to compare the degree of clonal expansion between repertoires. The short- and long-interval groups had more monoclonal frequency distributions compared to the no-NACT group. Taken together, TCR sequencing conducted in this study supports our previous finding that NACT exposure leads to a transient increase of intratumoral T cells in CLMs, while at the same time resulting in a persistent increase of TCR repertoire clonality. Both findings are in line with the hypothesis that NACT may cause immunogenic cell death, resulting in clonal expansion and T-cell response to tumor antigens, including neoantigens.

This work currently represents, to our knowledge, the largest study describing TCR sequencing in metastatic CRC. While studies have been performed in other cancer entities, including breast cancer [23, 24], bladder cancer [25], melanoma [26], lung cancer [27–29], and hepatocellular carcinoma [30], most of these have been smaller studies, including 12 to 40 patients. A notable exception was a study comparing TCR sequencing data from small cell lung cancer (SCLC, *n* = 67) [31] with non–small cell lung cancer (NSCLC, *n* = 236) [32], where SCLC tumors were characterized as “cold and heterogeneous” and less monoclonal compared to NSCLC. The estimated T-cell fractions in our samples were at an intermediate level (median 5.7%) compared to a very low value in SCLC and the much higher values observed in NSCLC (medians 1.7% and 21%, respectively). When comparing other TCR parameters, our cohort exhibited a higher number of unique clones than either of the lung cancer cohorts (median 15 596 vs. 510 and 3,246 for SCLC and NSCLC, respectively). While differences in absolute values between the studies may be caused by differences in sampling strategy, the detected differences in clonality based on TCR sequencing between the immunologically “cold” SCLC and “hot” NSCLC suggest that clonality could be a predictor of response to current immunotherapy approaches. Therefore, the differences in clonality observed between NACT exposed and unexposed CLM in this study might also be of clinical relevance in metastatic colorectal cancer (mCRC). One hypothesis is that NACT might evoke immune activation by induction of immunogenic cell death, resulting in improved responses to immunotherapy. This hypothesis is currently being tested by our team in the ongoing METIMMOX randomized clinical trial (NCT03388190).

Pairwise comparison of TCR datasets from analysis of 2 tissue aliquots from the same CLM had a high mean MHI (0.9), indicating almost complete overlap of clonal frequencies. In contrast, the MHI for datasets from different CLMs from the same patient was lower (0.3) but still exhibiting a higher degree of overlap than between repertoires from different CLM patients, where the overlap was almost nonexistent (0.0007). Although the numbers are low, this finding indicates high technical reproducibility of the TCR sequencing strategy. It also exemplifies that the immune microenvironment may vary between metastatic lesions from the same cancer within the same organ. In the SCLC and NSCLC comparison study, SCLC exhibited greater intratumoural variability (MHI <0.2) compared to NSCLC (MHI >0.8) [31]. This is concordant with the clonality parameter analysis and points to NSCLC having a more homogeneous neoantigen landscape than SCLC. An interesting follow-up study in our cohort would be to extend the analysis of pairwise comparison of CLM samples resected from the same patient by increasing the number of cases included.

B cells, in contrast to T cells, undergo somatic hypermutation. Previous studies of network repertoires generated from analysis of plasma cells show that B cells may exhibit highly centralized networks, with 1 clone highly connected to a large number of peripheral (but very similar) clones. The degree distribution of such networks follows a power law function, suggesting reactivity toward a single antigen [17, 22]. Very few of the TCR networks generated from our CLM cohort showed evidence of such sequential centralization. Instead, a small number of public clones (<10% of the detected clones) accounted for the majority of network connectivity. This is in line with prior findings from analysis of murine and human TCR repertoires [33] and suggests the existence of a small number of CDR3β subsequences that, although composing only a fraction of the entire clonal landscape, are overrepresented in the repertoires of many patients but also within individual patient repertoires. The finding is also congruent with the finding that a high proportion of the highly connected and public clones were linked to known pathology-associated TCRs in the McPAS database (Fig. 3G). Among the most frequent pathologies included influenza, tuberculosis, colorectal cancer, cytomegalovirus and Epstein–Barr virus. Some of these TCR subsequences could therefore be conserved in the Norwegian population due to common vaccination or viral exposure [34]. An alternative possibility, more specific to this cohort, is that they could represent a subset of T cells recognizing common tumor-associated antigens, or neoantigens, that are conserved in CRC, and these sequences could be potential candidates for further analyses [35, 36]. A third possibility is that the sequence convergence is generated by biases in V(D)J recombination [37, 38].

Although statistically significant, the differences between the NACT-exposed and nonexposed tumors with respect to T-cell infiltration and clonality were moderate, imposing limitations to the interpretation of the data. The methodological approach was also not able to distinguish T-cell subsets such as CD4 or CD8. The observed differences in T-cell infiltration seem to be driven by a subgroup of tumors that had a strong T-cell response to NACT in the short-interval group. Given that all the included patients had microsatellite-stable disease [9], such responses could indicate a CLM subgroup that is immunologically interesting with respect to the response to chemotherapy. For further studies, the time interval between chemotherapy exposure and TCR analysis should be standardized, as we have done in the ongoing METIMMOX trial (NCT03388190), where cytotoxic chemotherapy is administered sequentially with ICIs in microsatellite-stable mCRC. Although TCR sequencing data suggest the presence of NACT-driven clonal expansion, further exploration of the sequential makeup and possible sequential convergence of antigen binding TCR clones is warranted [39].

## Conclusions

Analysis of TCR repertoires in CLM confirmed our previous finding that NACT exposure was associated with a transient increase in T-cell infiltration, while a more persistent increase in clonality was observed independently of the timing of NACT administration and liver resection. The findings are consistent with a chemotherapy-driven clonal expansion and T-cell response, possibly to tumor neoantigens. The included samples represent an excellent starting point for further studies to identify potential public and private antigenic drivers. The results underline the importance of attention to the timing of drug administration in combination trials, and the standardized, high-throughput workflow supports the inclusion of TCR sequencing analysis in immunotherapy trials.

## Availability of Supporting Source Code and Requirements

Project name: airr_tools

Project homepage: https://github.com/eirikhoye/airr_tools

Data and code DOI: 10.5281/zenodo.7614598

biotoolsID: biotools:airr_tools

RRID: SCR_023297

Operating system(s): Platform independent

Programming language: R 4.0.5 or higher and Python 3.6 or higher

Other requirements: r-tidyverse 1.2.1, r-alakazam 1.0.2, r-bolstad2, r-ggpubr 0.4.0, imnet, pyspark, findspark

License: Open Source

## Supplementary Material

giad032_GIGA-D-22-00270_Original_Submission

giad032_GIGA-D-22-00270_Revision_1

giad032_GIGA-D-22-00270_Revision_2

giad032_Response_to_Reviewer_Comments_Original_Submission

giad032_Response_to_Reviewer_Comments_Revision_1

giad032_Reviewer_1_Report_Original_Submission James M Heather -- 11/28/2022 Reviewed

giad032_Reviewer_2_Report_Original_Submission Satoshi Ueha -- 12/2/2022 Reviewed

giad032_Reviewer_2_Report_Revision_1 Satoshi Ueha -- 2/16/2023 Reviewed

giad032_Supplemental_File

## Data Availability

The data sets supporting the results of this article are available in the Adaptive immuneACCESS repository [40]. All supporting data are available in the *GigaScience* GigaDB database [41].

## Additional Files

**Supplementary Table S1**. Clonal amino acid hits for pathogens listed in the McPAS database, for top 10% most public and most connected clones.

**Supplementary Fig. S1**. Kaplan–Meier plot comparing the effect of clonality on overall survival. Estimated from time of CLM resection and censored at time of death.

**Supplementary Fig. S2**. Comparison of clonality and the location of the primary tumor.

**Supplementary Fig. S3**. Association between (A) sequencing coverage and T-cell fraction, (B) nanogram gDNA and T-cell fraction, and (C) nanogram DNA and sequencing coverage. Red dots are samples with T-cell fraction >0.15. Dotted line is the minimal cutoff point for sequencing coverage (5).

**Supplementary Fig. S4**. Comparison of Hill-based clonality estimate and sequencing coverage. Dotted line is the minimal cutoff point for sequencing coverage.

## Abbreviations

AUC: area under the curve; CDR3: complementarity determining region 3; CI: confidence interval; CLM: colorectal cancer liver metastasis; CRC: colorectal cancer; gDNA: genomic DNA; ICI: immune checkpoint inhibitor; LD: Levenshtein distance; MHI: Morisita–Horn index; MSS: microsatellite stable; NACT: neoadjuvant chemotherapy; NSCLC: non–small cell lung cancer; PCR: polymerase chain reaction; SCLC: small cell lung cancer; TCR: T-cell receptor.
