# Supplementary material for: T cell receptor repertoire sequencing reveals chemotherapy-driven clonal expansion in colorectal liver metastases
Source: Gigascience. 2023 May 10;12:giad032. doi: 10.1093/gigascience/giad032 (PMC10170408; doi:10.1093/gigascience/giad032)
Supplement: giad032_GIGA-D-22-00270_Original_Submission [file giad032_giga-d-22-00270_original_submission.pdf]

GigaScience

T cell receptor repertoire sequencing reveals chemotherapy-driven clonal expansion in colorectal liver metastases

--Manuscript Draft--

**Manuscript Number:**	GIGA-D-22-00270
**Full Title:**	T cell receptor repertoire sequencing reveals chemotherapy-driven clonal expansion in colorectal liver metastases
**Article Type:**	Research
**Funding Information:**	Helse Sør-Øst RHF (2018014) — Professor Kjersti Flatmark<br>Kreftforeningen (215817) — Dr Victor Greiff
**Abstract:**	Background<br><br>Colorectal liver metastasis (CLM) is a leading cause of colorectal cancer mortality, and the response to immune checkpoint inhibition (ICI) in microsatellite stable CRC has been disappointing. Administration of cytotoxic chemotherapy may cause increased density of tumour infiltrating T cells, which has been associated with improved response to ICI. This study aimed to quantify and characterize T cell infiltration in CLM using T cell receptor (TCR) repertoire sequencing. Eighty-five resected CLM from patients included in the Oslo CoMet study were subjected to TCR repertoire sequencing. Thirty-five and 15 patients had received neoadjuvant chemotherapy (NACT) within a short or long interval, respectively, prior to resection, while 35 patients had not been exposed to NACT. T cell fractions were calculated, repertoire clonality was analysed based on Hill evenness curves, and TCR sequential convergence was assessed using network analysis.<br><br>Results<br><br>Increased T cell fractions (10.6% vs 6.3%) were detected in CLM exposed to NACT within a short interval prior to resection, while modestly increased clonality was observed in NACT exposed tumours independently of the timing of NACT administration and surgery. While private clones made up >90% of detected clones, network connectivity analysis revealed that public clones contributed the majority of TCR sequence convergence.<br><br>Conclusions<br><br>TCR repertoire sequencing can be used to quantify T cell infiltration and clonality in clinical samples. This study provides evidence to support chemotherapy-driven T cell clonal expansion in CLM in a clinical context.
**Corresponding Author:**	Kjersti Flatmark, PhD<br>Radiumhospitalet Institutt for kreftforskning: Oslo Universitetssykehus Institutt for kreftforskning<br>Oslo, NORWAY
**Corresponding Author Secondary Information:**	
**Corresponding Author's Institution:**	Radiumhospitalet Institutt for kreftforskning: Oslo Universitetssykehus Institutt for kreftforskning
**Corresponding Author's Secondary Institution:**	
**First Author:**	Eirik Høye
**First Author Secondary Information:**	
**Order of Authors:**	Eirik Høye

Powered by Editorial Manager® and ProduXion Manager® from Aries Systems Corporation

	Vegar Johansen Dagenborg
	Annette Torgunrud
	Christin Lund-Andersen
	Åsmund Avdem Fretland
	Susanne Lorenz
	Bjørn Edwidn
	Eivind Hovig
	Bastian Fromm, PhD
	Else Marit Inderberg, PhD
	Victor Greiff, PhD
	Anne Hansen Ree, PhD
	Kjersti Flatmark, PhD
**Order of Authors Secondary Information:**	
**Additional Information:**	
**Question**	**Response**
Are you submitting this manuscript to a special series or article collection?	No
**Experimental design and statistics**<br><br>Full details of the experimental design and statistical methods used should be given in the Methods section, as detailed in our Minimum Standards Reporting Checklist. Information essential to interpreting the data presented should be made available in the figure legends.<br><br>Have you included all the information requested in your manuscript?	Yes
**Resources**<br><br>A description of all resources used, including antibodies, cell lines, animals and software tools, with enough information to allow them to be uniquely identified, should be included in the Methods section. Authors are strongly encouraged to cite Research Resource Identifiers (RRIDs) for antibodies, model organisms and tools, where possible.	Yes

Powered by Editorial Manager® and ProduXion Manager® from Aries Systems Corporation

Have you included the information requested as detailed in our Minimum Standards Reporting Checklist?	
**Availability of data and materials**<br><br>All datasets and code on which the conclusions of the paper rely must be either included in your submission or deposited in publicly available repositories (where available and ethically appropriate), referencing such data using a unique identifier in the references and in the "Availability of Data and Materials" section of your manuscript.<br><br>Have you have met the above requirement as detailed in our Minimum Standards Reporting Checklist?	Yes

Manuscript

Click here to access/download;Manuscript;CLM-TCRseq_gigascience_v3.docx

T cell receptor repertoire sequencing reveals chemotherapy-driven clonal expansion in colorectal liver metastases

Eirik Høye[1,2], Vegar J. Dagenborg[1,3], Annette Torgunrud[1], Christin Lund-Andersen[1,2], Åsmund A. Fretland[4,5], Susanne Lorenz[6], Bjørn Edwin[2,4,5,], Eivind Hovig[7], Bastian Fromm[8], Else M. Inderberg[9], Victor Greiff[10], Anne H. Ree[2,11], Kjersti Flatmark[1,2,3,*]

[1] Department of Tumor Biology, Institute for Cancer Research, The Norwegian Radium Hospital, Oslo University Hospital 0379 Oslo, Norway

[2] Institute of Clinical Medicine, Medical Faculty, University of Oslo, 0318 Oslo, Norway

[3] Department of Gastroenterological Surgery, The Norwegian Radium Hospital 0379 Oslo, Norway

[4] The Intervention Centre, Rikshospitalet, Oslo University Hospital, 0372 Oslo, Norway

[5] Department of Hepato-Pancreato-Biliary Surgery, Rikshospitalet, Oslo University Hospital, 0372 Oslo, Norway

[6] Department of Core Facilities, Institute for Cancer Research, The Norwegian Radium Hospital, Oslo University Hospital, 0379 Oslo, Norway

[7] Center for Bioinformatics, Department of Informatics, University of Oslo, 0316 Oslo, Norway

[8] The Arctic University Museum of Norway, UiT – The Arctic University of Norway, 9037 Tromsø, Norway

[9] Translational Research Unit, Department of Cellular Therapy, Oslo University Hospital, 0379 Oslo, Norway

[10] Department of Immunology, University of Oslo and Oslo University Hospital, 0372 Oslo, Norway

[11] Department of Oncology, Akershus University Hospital, 1478 Lørenskog, Norway

* Corresponding author at: Department of Tumor Biology, Institute for Cancer Research, The Norwegian Radium Hospital, 0379 Oslo
E-mail address: kjersti.flatmark@rr-research.no

Abstract

Background: Colorectal liver metastasis (CLM) is a leading cause of colorectal cancer mortality, and the response to immune checkpoint inhibition (ICI) in microsatellite stable CRC has been disappointing. Administration of cytotoxic chemotherapy may cause increased density of tumour infiltrating T cells, which has been associated with improved response to ICI. This study aimed to quantify and characterize T cell infiltration in CLM using T cell receptor (TCR) repertoire sequencing. Eighty-five resected CLM from patients included in the Oslo CoMet study were subjected to TCR repertoire sequencing. Thirty-five and 15 patients had received neoadjuvant chemotherapy (NACT) within a short or long interval, respectively, prior to resection, while 35 patients had not been exposed to NACT. T cell fractions were calculated, repertoire clonality was analysed based on Hill evenness curves, and TCR sequential convergence was assessed using network analysis.

Results: Increased T cell fractions (10.6% vs 6.3%) were detected in CLM exposed to NACT within a short interval prior to resection, while modestly increased clonality was observed in NACT exposed tumours independently of the timing of NACT administration and surgery. While private clones made up >90% of detected clones, network connectivity analysis revealed that public clones contributed the majority of TCR sequence convergence.

Conclusions: TCR repertoire sequencing can be used to quantify T cell infiltration and clonality in clinical samples. This study provides evidence to support chemotherapy-driven T cell clonal expansion in CLM in a clinical context.

Key words

- Colorectal cancer
- T cell receptor sequencing
- Liver metastasis
- Neoadjuvant chemotherapy

- Clonal expansion
- Clinical samples

Introduction

Increased understanding of how the immune system is involved in cancer development and progression has resulted in development of therapeutic interventions successfully targeting the immune system, such as the immune checkpoint inhibitors (ICI) targeting the PD-1/PD-L1 axis. Mismatch repair deficient cancers with high tumour mutational burden have been shown to respond strongly to ICI regardless of histologic type [1], which has led to interest in identification of specific tumour neoantigens. Characterisation of tumour antigen-specific T cells has therefore become an important step to further understand anti-tumour immunity. The non-coding part of the genome is increasingly perceived as a major contributor to tumour neoantigens, as up to 70 % of the genome is transcribed in some form [2]. Abnormally expressed RNA in tumour tissue could therefore contribute as neoantigens in cancers with modest tumour mutational burden. T cells recognise their cognate antigens through interaction with the peptide-MHC complexes presented on the surface of target cells via the complementarity determining region 3 (CDR3) of the T cell receptor (TCR). Recent advances in deep sequencing of the TCR CDR3 region have enabled quantification of T cell clones with the same antigen specificity and characterisation of TCR repertoires across biological compartments and over time [3,4]. Applying this technology to metastatic tumor samples represents an important opportunity to further understand T cell immunity in metastatic cancer and possibly identify neoantigens associated with immune responses.

Colorectal cancer (CRC) accounts for about 10 % of all diagnosed cancers and cancer-related deaths worldwide, and colorectal liver metastasis (CLM) is a leading cause of CRC-related mortality [5]. Surgery is a curative treatment option for a minority of patients with limited metastatic disease, but for most patients with CLM, systemic chemotherapy is the main treatment option, and the survival rates are poor [6]. Durable responses to ICI have been observed in CRC patients with microsatellite instable tumours, but with the majority of patients suffering from microsatellite stable (MSS) cancers, responses are generally disappointing [7]. Still, in MSS CRC there is evidence to suggest that the microenvironmental immune

contexture is important, such as in primary CRC, where a high density of tumour infiltrating T cells was shown to strongly correlate with a favourable long-term outcome [8]. In previous studies of CLM we observed up-regulation of immune-related genes and increased T cell intratumoural densities after exposure to neoadjuvant chemotherapy (NACT) [9,10]. This suggests that NACT can modify the CLM immune microenvironment, possibly by induction of immunogenic cell death, towards a state that could potentially be more responsive to ICI.

In this work, we have sequenced TCR repertoires in resected CLM from 85 patients included in the OSLO-COMET study and repertoires were compared according to NACT exposure to analyse T cell fractions and clonality. In addition, network analysis was used to assess sequential convergence reported to be associated with antigen-experienced repertoires.

Methods

Patient samples

CLM samples were collected from 85 patients included in the *Oslo Randomized Laparoscopic Versus Open Liver Resection for Colorectal Metastases Study* (OSLO-COMET study; NCT01516710) (for clinical data, see Table 1). Written informed consent was obtained from all participating patients. The OSLO-COMET study was approved by Norway's Regional Committees for Medical and Health Research Ethics (ID# 2011/1285/REK sør-øst B). Neoadjuvant chemotherapy (NACT) was administered to 50 patients (59%), while 35 patients (41%) did not receive NACT (no-NACT group) (Fig. 1a). The NACT regimen, the timing of liver resection after NACT, and the number of NACT cycles were not predefined by the OSLO-COMET study protocol, but were decided for the individual patient by a multidisciplinary team [9]. We previously determined that a 9.5-week interval between completion of NACT and liver resection was a cut-off for observing an increase of intra-tumoral T cell density [9]. Applying the cut-off to this cohort, 35 (41%) and 15 (18%) patients had received NACT less, and more than 9.5 weeks prior to liver resection, respectively (hereafter termed the short-interval (min-max 2.9-9.5 weeks) and long-interval (min-max 9.5-25.9 weeks) groups. Tumour samples were fresh frozen in liquid nitrogen and stored at -80˚C. The tumour content was evaluated by the study pathologist and the tissues were processed and homogenized as previously described [9]. Briefly, DNA was isolated using the Allprep DNA/RNA/miRNA Universal Kit (Qiagen, Düsseldorf, Germany; Cat. No. 80224) and DNA purity was determined using the Nanodrop 2000 spectrophotometer (Thermo Fisher, Waltham, Massachusetts, USA). Aliquots were diluted to 200 ng/µL, as measured with the QuBit dsDNA Broad Range Assay Kit (Thermo Fisher; Cat. No. Q32850). Three of the 85 patients included in the study had two TCR repertoire datasets from the same metastasis but different tissue aliquot, representing technical replicates. For four patients, TCR repertoire datasets were derived from two separate metastases resected at the same procedure, providing information about potential heterogeneity of TCR repertoires in metastases located in the same liver.

T cell receptor sequencing

TCR repertoire sequencing libraries were prepared using the hsTCRB v3 immunoSEQ library preparation kit (Adaptive Biotechnologies, Seattle, Washington, USA). Briefly, two 16-µL polymerase chain reaction (PCR) replicates were prepared for each biological sample, with DNA content within the range recommended by the manufacturer for non-lymphoid tissues. The library preparation protocol uses multiplex PCR with primers for all possible V and J fragments in the TCR-beta gene with adjustment of primer concentrations to account for variable primer efficiencies. Synthetic repertoires with known quantities are present in each PCR reaction to allow accurate quantification of T cells in each sample [11]. Pooled libraries were sequenced using the NextSeq 500/550 Mid Output kit v2.5 (150 cycles) (Illumina, San Diego, California, USA). Bioinformatics processing of raw data was done by Adaptive Biotechnologies proprietary analysis pipeline which provided data on the immunoSEQ rearrangement-level file format. The T cell fraction was determined by dividing the number of detected rearranged CDR3β sequences (as a proxy for the number of T cells) with the total number of genomes in the sample which was calculated by total amount of DNA/6.6 pg (mean DNA content/cell). T cells with identical CDR3β sequence and length were defined as one clonotype for subsequent analyses. As previously described, a clone was considered "private" if detected in only one patient, with "public" clones being present in more than one patient [12], and the sharing level was defined as the number of patients in whom a specific clonotype was detected.

TCR repertoire clonality using Hill diversity and evenness profiles

The alakazam v1.1.0 R package [13] was used to generate Hill diversity and evenness profiles for each dataset, as previously described [14]. The range of q parameters was set between q=0 to q=10, with steps of 0.2. Evenness profiles are defined by the equation ${}^{\alpha}E = {}^{\alpha}D / SR$ (or the Hill diversity profile divided by Hill diversity at q=0), as previously described in [15]. For each repertoire, a clonality index, defined as ten minus the area under the curve (AUC), was calculated for individual evenness curves using the sintegral() function from the Bolstad2 v1.0-

28 R package [16], which gives a parameter ranging from zero to ten, with higher values signifying more oligoclonal repertoires.

Analysis of within TCR repertoire TCR sequence similarity using Levenshtein distance networks

To analyse TCR sequence similarity within T cell repertoires, the ImNet v0.2.1 package was used to generate Levenshtein distance (LD) [17] matrices for all unique CDR3β sequences in each dataset. These distance matrices were then used to construct CDR3β sequential convergence networks, where connections were built between CDR3β sequences at LD=1. Visualization of the networks was accomplished with Cytoscape v3.9.0 [18]. The resulting networks illustrate the overall similarity of CDR3β sequences within a T cell repertoire and the presence of subclusters of CDR3β sequences. Global parameters calculated for each network included the number of nodes (unique clones) and number of edges (clonal connections), and the network connectivity fraction was calculated by dividing edges with nodes. The powerlaw() R package [19] was used to assess whether the network degree distribution followed a power law function. A goodness of fit test was conducted, where the null hypothesis was power law distribution. X-min value was determined for each distribution. A degree distribution with a power law goodness of fit p-value greater than 0.1 was considered a plausible power law distribution. Local parameters to characterize individual clones included the number of degrees (connections between clones in a network) and the sharing level (number of patients where a clone was detected). As was done previously in [12,20], to compare network connectivity of public and private clones, clones from all repertoires were classified as public or private, subsampled to 1000 clones to compensate for the numerical overrepresentation of private clones, and networks were generated with the same method as above.

To further explore potential associations between TCR connectivity, sharing level, clonal expansion and potential disease pathologies, the McPAS database [21] was used. Two groups of TCRs were defined, one consisting of top 10% of clones with highest connectivity and/or sharing level, the other consisting of the top 10% most clonally expanded TCRs. The fraction

of TCRs that matched at LD = 0 with known pathology associated TCRs in McPAS was calculated for each sample.

Analysis of overlapping clones between repertoires

The Morisita Horn index (MHI) was used to assess clonal similarity between repertoires. This index shows the overall clonal overlap between two repertoires, weighted by clonal frequency, ranging from 0 (no overlap) to 1 (complete overlap). A combined rearrangement file was downloaded from the immunoSEQ Analyzer. This file format has all clones across all datasets for each row, and the count of that clonotype in each dataset for columns. This was input to the divo R package mh() v1.0.1 function, which yielded pairwise MHI comparison for all datasets. Pairwise comparisons were made between repertoires from all analysed patients; in addition, comparisons were made between repertoires generated from different aliquots from the same metastasis (n=3), and repertoires from different metastases in the same patient (n=4).

Statistical analyses

For T cell fraction analysis, one dataset per patient was included (n=85), while for subsequent analyses, in order to avoid bias due to low frequency clones, eight datasets with sequencing coverage <5 were excluded (total number of samples available for downstream analysis, n=77). T cell fractions and clonality were described using mean and 95% confidence intervals (CI). Mean T cell fractions and clonality in the NACT administration groups were compared using the Welch t-test using the compare_means() function from ggpubr v0.4.0. Comparison of clonality against the location of the pCRC was also made. Linear regression was used to analyse the relationship between clonality and the number of T cells, and the network connectivity fraction and number of clones, using the lm() function in base R v4.0.5. Associations between clonality and primary tumour location (right colon, left colon or rectum) were also analysed using the Welch t-test using the compare_means() function form ggpubr v0.4.0. Overall survival was measured from the time of CLM resection. The last liver resection

date was on 28 January 2016, and the censoring date was on 8 January 2020. The Kaplan-Meier method was used to estimate patient survival, while the log-rank test was used to see if there was a difference between survival curves of patients with low, medium or high clonality, using the survival v3.2-12 package. P-values <0.05 were considered to indicate statistical significance.

Role of Funding source

This work was financially supported by the South-Eastern Norway Regional Health Authority [grant #2018014, to KF] to fund the PhD position for EH, and Norwegian Cancer Society [grant #215817, to VG].

Results

High T cell fraction was associated with a short interval between NACT administration and surgery

The mean number of productive CDR3β DNA templates per sample was 83 098, with 95% CI [66 615-99 582]. When normalized against the total amount of genomic DNA, the mean T cell fraction was 8.1%, with 95% CI [6.5-9.6%]. The mean T cell fraction was higher in the short-interval group (10.6% [7.4-13.8%]) compared to the no-NACT group (6.3% [4.8-7.9%]), p=0.02 (Fig. 1b). The T cell fraction in the long-interval group (6.2% [3.4-8.9%]) was also significantly lower compared to the short-interval group (p=0.04), while there was no difference compared to the no-NACT group.

NACT exposure was associated with more clonal TCR repertoires

In total, 1 413 435 unique clones were identified across all datasets, median 15 596 (min-max, 1476-66 976). Hill diversity profiles (Fig. 2a) showed considerable variability at the species richness range (q=0) (mean 16 000, 95% CI [13 611-18 388]). Both the short-interval (18 020 [13 524-22 515]) and long-interval groups (16 079 [10 394-21 764]) exhibited non-significant trends towards a higher number of unique clones compared to the no-NACT group (14 069 [10 833-17 304]). At increasing values of q, the mean in the short- and long-interval groups intersected and fell below the mean of the no-NACT group, but with overlapping CIs. When comparing the Hill evenness profiles (Fig. 2b), which are diversity profiles normalized against the number of unique clones (also called species richness), the short- and long-interval groups had very similar means, and the curve for both groups were lower than for the no-NACT group, with non-overlapping 95% CI. Comparison of clonality based on AUC calculations from individual evenness curves showed that the short-interval (5.8 [5.5-6.2]) and long-interval (5.7 [5.2-6.2]) groups had higher mean values than the no-NACT group (5.0 [4.6-5.4]) (p=0.004 and p=0.03, respectively) (Fig. 2c). Furthermore, regression analysis revealed a modest association between a high absolute number of T cells and clonality (Fig. 2d). There were no

associations between clonality and overall survival (Supplementary Fig 1), but a non-significant trend towards increased clonality was observed when comparing samples from right-sided with left-sided primary tumours (median og y, p=0.08, Supplementary Fig 2).

T cell similarity networks clustered around publicly conserved sub-sequences

The mean number of nodes detected was 16 056, 95% CI [13 637-18 475], while the mean number of edges was 4133 [3015-5251], generating a mean network connectivity fraction of 19.0% [16.5-21.5%]. The connectivity fraction increased linearly as a function of the total number of clones, from less than 5% in small networks, to greater than 50% in large networks (Fig. 3a). While the majority of the networks did not follow a power-law distribution (Fig. 3b), four short-interval and five no-NACT repertoires passed the power law fit test. The mean clonality for the networks that passed the test was 4.4, which was lower than the mean clonality across all datasets of 5.5. Clonally expanded datasets were therefore not associated with power law degree distributions. Instead, connectivity was associated with the sharing level, where the number of degrees increased linearly with increasing sharing level (Fig. 3c). The majority of the detected clones were private (90.3 %), while 9.7% of the clones were public. (Fig. 3d). The public clones exhibited a much higher level of connectivity than the private clones, with mean connectivity fraction of 20.5% [19.2%-21.8%] compared to 0.4% [0.3%-0.4%], respectively (Fig. 3d). This finding is in line with the MHI comparisons (Fig. 3e), where repertoires from different patients had very low overlap of mean 0.0007 (min-max, 0-0.07). MHI calculated from repertoires generated from different aliquots from the same metastasis were high, with a mean of 0.93 (min-max, 0.86-0.97), while repertoires from different metastases in the same patient exhibited moderate overlap, with a mean MHI of 0.5 (min-max=0.09-0.97). The TCR clones with highest connectivity and/or sharing level were more commonly associated with known pathogens (10% of clones), according to the McPAS database, compared with the most expanded TCR clones (3% of clones) (Fig. 3g). Interestingly, the five most common pathologies detected were influenza, tuberculosis, colorectal cancer, cytomegalovirus and epstein barr virus.

Three representative networks are shown in Fig. 4. A network from the short-interval group was in the monoclonal end of the spectrum (Clonality=7.1) had a connectivity profile with 2907 clonotypes, 188 connections and a network connectivity fraction of 7% (Sample 122; Fig. 4a**)**. The most expanded clone (2053 T cells) was not detected in any other samples, representing a private clone. A more heteroclonal network (Clonality=3.3) from the no-NACT group had a very similar connectivity profile, exhibiting 3005 clones, 165 connections, and a network connectivity fraction of 6% (Sample 40; Fig. 4b). Finally, a very large monoclonal network (Clonality=6.9) is visualized, with 34 849 clones and 13 527 connections, and a high network connectivity fraction of 39% (Sample 37; Fig. 4c). Again, the majority of expanded clones were private to this sample.

Discussion

In this work, using TCR sequencing, we found that the T cell fraction was significantly higher in tumours with a short interval between NACT exposure and liver resection compared to tumours not exposed to NACT. This finding is in line with previously published work from our group, showing higher T cell density in the short-interval group compared to the no-NACT and long-interval groups [9]. Considering that different parts of the tumours were analysed (whole section immunohistochemistry versus snap frozen tissue from the surgical samples) and that different methods were used to quantify T cells, the concordance is remarkable.

Although a significant increase of infiltrating T cells was detected in the short-interval group only, analysis of Hill diversity curves revealed that both the short- and long-interval groups had more uneven clonal frequency distributions compared to the no-NACT group. As previously explained by Hill [14], the diversity at q=0 corresponds to the total number of clones in the repertoire (clonal richness). Because clonal richness is influenced by sampling depth and the presence of rare clones, the frequencies should also be assessed across a range of diversity parameters [14,15], rather than by commonly used single point estimates, to obtain a complete picture of clonal frequency distributions. At q values greater than 2 (Simpson index [14]), the influence of rare clones on the Hill diversity estimate becomes negligible, and is instead influenced by abundant clones. For our datasets, the short-interval group diversity profile was higher at q=0, corresponding with the higher T cell fraction observed in this group. Yet, it intersected and became similar to the no-NACT profile at higher values of q, indicating that the higher clonal richness was driven by rare clones, likely reflecting the higher T cell fraction in this group. However, the slope of both short-interval and long-interval profiles were steeper than the no-NACT profile, still suggesting qualitative differences in the clonal frequency distribution that could be related to NACT exposure. Hill diversity estimates cannot be used to quantitatively compare the clonality of repertoires if the number of sampled T cells is very different. Instead, Hill evenness profiles, which normalize datasets by clonal richness, will provide a more correct comparison of clonality between repertoires. We therefore calculated

AUC values from Hill evenness profiles to compare the degree of clonal expansion between repertoires. The short- and long-interval groups had more monoclonal frequency distributions compared to the no-NACT group. Taken together, TCR sequencing conducted in this study supports our previous finding that NACT exposure leads to a transient increase of intratumoral T cells in CLM, while at the same time resulting in a persistent increase of TCR repertoire clonality. Both findings are in line with the hypothesis that NACT may cause immunogenic cell death, resulting in clonal expansion and T cell response to tumour antigens, including neoantigens.

This work currently represents, to our knowledge, the largest study describing TCR sequencing in metastatic CRC. While studies have been performed in other cancer entities, including breast cancer [22,23], bladder cancer [24], melanoma [25], lung cancer [26–28] and hepatocellular carcinoma [29], most of these have been smaller studies, including 12 to 40 patients. A notable exception was a study comparing TCR sequencing data from small cell lung cancer (SCLC, n=67) [30] with non-small cell lung cancer (NSCLC, n=236) [31], where SCLC tumours were characterized as “cold and heterogeneous” and less monoclonal compared to NSCLC. The estimated T cell fractions in our samples were at an intermediate level (median 5.7%) compared to a very low value in SCLC and the much higher values observed in NSCLC (medians 1.7% and 21%, respectively). When comparing other TCR parameters, our cohort exhibited a higher number of unique clones than either of the lung cancer cohorts (median 15 596, versus 510 and 3246, for SCLC and NSCLC respectively). While differences in absolute values between the studies may be caused by differences in sampling strategy, the detected differences in clonality based on TCR sequencing between the immunologically “cold” SCLC and “hot” NSCLC suggest that the differences in clonality between NACT exposed and unexposed CLM might be of clinical relevance.

Pairwise comparison of TCR datasets from analysis of two tissue aliquots from the same CLM had a high mean MHI (0.9), indicating almost complete overlap of clonal frequencies. In

contrast, the MHI for datasets from different CLM from the same patient was lower (0.3), but still exhibiting a higher degree of overlap than between repertoires from different CLM patients, where the overlap was almost non-existent (0.0007). Although the numbers are low, this finding indicates high technical reproducibility of the TCR sequencing strategy. It also exemplifies that the immune microenvironment may vary between metastatic lesions from the same cancer within the same organ. In the SCLC and NSCLC comparison study, SCLC exhibited greater intratumoural variability (MHI<0.2) compared to NSCLC (MHI>0.8) [30]. This is concordant with the clonality parameter analysis, and points to NSCLC having a more homogenous neo-antigen landscape than SCLC. An interesting follow-up study in our cohort would be to extend the analysis of pairwise comparison of CLM samples resected from the same patient by increasing the number of cases included.

B cells, in contrast to T cells, undergo somatic hypermutation. Previous studies of network repertoires generated from analysis of plasma cells show that B cells may exhibit highly centralized networks, with one clone highly connected to a large number of peripheral (but very similar) clones. The degree distribution of such networks follows a power law function, suggesting reactivity towards a single antigen [17,32]. Very few of the TCR networks generated from our CLM cohort showed evidence of such sequential centralization. Instead, a small number of public clones (<10% of the detected clones) accounted for the majority of network connectivity. This is in line with prior findings from analysis of murine and human TCR repertoires [33], and suggests the existence of a small number of CDR3β sub-sequences that, although composing only a fraction of the entire clonal landscape, are overrepresented in the repertoires of many patients, but also within individual patient repertoires. The finding is also congruent with the finding that a high proportion of the highly connected and public clones were linked to known pathology-associated TCRs in the McPAS database (fig. 3g). Among the most frequent pathologies included influenza, tuberculosis, colorectal cancer, cytomegalovirus and epstein barr virus. Some of these TCR sub-sequences could therefore be conserved in the Norwegian population due to common vaccination or viral exposure [34].

An alternative possibility, more specific to this cohort, is that they could represent a subset of T cells recognizing common tumour associated antigens, or neoantigens, that are conserved in CRC, and these sequences could be potential candidates for further analyses [35,36].

Although statistically significant, the differences between the NACT exposed and non-exposed tumours with respect to T cell infiltration and clonality were moderate, imposing limitations to the interpretation of the data. The observed differences in T cell infiltration seem to be driven by a subgroup of tumours that had a strong T cell response to NACT in the short-interval group. Given that all the included patients had microsatellite stable disease [9], such responses could indicate a CLM subgroup that is immunologically interesting with respect to the response to chemotherapy. For further studies, the time interval between chemotherapy exposure and TCR analysis should be standardized, as we have done in the ongoing METIMMOX trial (NCT03388190), where cytotoxic chemotherapy is administered sequentially with ICI in microsatellite stable mCRC. Although TCR sequencing data suggests the presence of NACT-driven clonal expansion, further exploration of the sequential makeup and possible sequential convergence of antigen binding TCR clones is warranted [37].

Conclusions

Analysis of TCR repertoires in CLM confirmed our previous finding that NACT exposure was associated with a transient increase in T cell infiltration, while a more persistent increase in clonality was observed independently of the timing of NACT administration and liver resection. The findings are consistent with a chemotherapy-driven clonal expansion and T cell response, possibly to tumour neoantigens. The included samples represent an excellent starting point for further studies to identify potential public and private antigenic drivers. The results underline the importance of attention to the timing of drug administration in combination trials, and the standardized, high-throughput workflow supports the inclusion of TCR sequencing analysis in immunotherapy trials.

Availability of Source Code

Project name: airr_tools

Project home page: https://github.com/eirikhoye/airr_tools

Operating system(s): Platform independent

Programming language: R 4.0.5 or higher, and python 3.6 or higher

Other requirements: r-tidyverse 1.2.1, r-alakazam 1.0.2, r-bolstad2, r-ggpubr 0.4.0, imnet, pyspark, findspark

License: Open Source

Data availability

The data sets supporting the results of this article are available in the Adaptive immuneACCESS repository, [DOI to be added upon acceptance, see below for temporary link]. Bioinformatics pipeline, code, metadata and statistical analysis can be found at GitHub at https://github.com/eirikhoye/airr_tools.

FOR REVIEWERS:

Temporary link to Adaptive repository

clients.adaptivebiotech.com

email: hoye-review@adaptivebiotech.com

password: hoye2022review

Abbreviations

ICI: Immune checkpoint inhibitor

CDR3: Complementarity determining region 3

TCR: T cell receptor

CRC: Colorectal cancer

CLM: CRC liver metastasis

MSS: Microsatellite stable

NACT: Neoadjuvant chemotherapy

PCR: polymerase chain reaction

LD: Levenshtein distance

MHI: Morisita Horn index

CI: Confidence interval

SCLC: Small cell lung cancer

NSCLC: Non-small cell lung cancer

Ethical Approval

Written informed consent was obtained from all participating patients. The OSLO-COMET study was approved by Norway's Regional Committees for Medical and Health Research Ethics (ID# 2011/1285/REK sør-øst B).

Additional Files

Supplementary file. Supplementary Figure 1. Kaplan Meier plot comparing the effect of clonality on overall survival. Estimated from time of CLM resection and censored at time of death. Supplementary Figure 2. Comparison of clonality and the location of the primary tumour.

Competing Interests

The authors declare no conflict of interest exists.

Funding

South-Eastern Norway Regional Health Authority (grant#2018014/K.F.), Norwegian Cancer Society (grant#215817/V.G.)

Author's Contributors

E.H.: data curation, formal analysis, investigation, methodology, software, visualization, writing – original draft; V.J.D.: conceptualization, data curation, investigation, project

administration, resources, writing – review & editing; A.T.: data curation, investigation, project administration, resources, writing – review & editing; C.L.A.: data curation, investigation, project administration, resources, writing – review & editing; Å.A.F.: data curation, project administration, resources, writing – review & editing; S.L.: conceptualization, resources, writing – review & editing, B.E.: funding acquisition, project administration, resources, writing – review & editing; E.H.: data curation, resources, writing – review & editing; B.F.: conceptualization, investigation, supervision, writing – review & editing; E.M.I.: conceptualization, supervision, writing – original draft; V.G.: formal analysis, methodology, supervision, writing – original draft; A.H.R.: conceptualization, funding acquisition, supervision, writing – review & editing; K.F.: conceptualization, data curation, formal analysis, funding acquisition, investigation, methodology, project administration, resources, supervision, writing – original draft.

Acknowledgements

Not applicable.

References

1. Le DT, Durham JN, Smith KN, Wang H, Bartlett BR, Aulakh LK, et al.. Mismatch repair deficiency predicts response of solid tumors to PD-1 blockade. *Science*. 357:409–132017;

2. Laumont CM, Vincent K, Hesnard L, Audemard É, Bonneil É, Laverdure J-P, et al.. Noncoding regions are the main source of targetable tumor-specific antigens. *Sci Transl Med*. 2018; doi: 10.1126/scitranslmed.aau5516.

3. Rosati E, Dowds CM, Liaskou E, Henriksen EKK, Karlsen TH, Franke A. Overview of methodologies for T-cell receptor repertoire analysis. *BMC Biotechnol*. 17:612017;

4. Brown AJ, Snapkov I, Akbar R, Pavlović M, Miho E, Sandve GK, et al.. Augmenting adaptive immunity: progress and challenges in the quantitative engineering and analysis of adaptive immune receptor repertoires. *Mol Syst Des Eng*. The Royal Society of Chemistry; 4:701–362019;

5. Bray F, Ferlay J, Soerjomataram I, Siegel RL, Torre LA, Jemal A. Global cancer statistics 2018: GLOBOCAN estimates of incidence and mortality worldwide for 36 cancers in 185 countries. *CA Cancer J Clin*. 68:394–4242018;

6. Ferlay J, Soerjomataram I, Dikshit R, Eser S, Mathers C, Rebelo M, et al.. Cancer incidence and mortality worldwide: sources, methods and major patterns in GLOBOCAN 2012. *Int J Cancer*. 136:E359-862015;

7. Cohen R, Rousseau B, Vidal J, Colle R, Diaz LA Jr, André T. Immune Checkpoint Inhibition in Colorectal Cancer: Microsatellite Instability and Beyond. *Target Oncol*. 15:11–242020;

8. Galon J, Costes A, Sanchez-Cabo F, Kirilovsky A, Mlecnik B, Lagorce-Pagès C, et al.. Type, density, and location of immune cells within human colorectal tumors predict clinical outcome. *Science*. 313:1960–42006;

9. Dagenborg VJ, Marshall SE, Yaqub S, Grzyb K, Boye K, Lund-Iversen M, et al.. Neoadjuvant chemotherapy is associated with a transient increase of intratumoral T-cell density in microsatellite stable colorectal liver metastases. *Cancer Biol Ther*. 21:432–402020;

10. Østrup O, Dagenborg VJ, Rødland EA, Skarpeteig V, Silwal-Pandit L, Grzyb K, et al.. Molecular signatures reflecting microenvironmental metabolism and chemotherapy-induced immunogenic cell death in colorectal liver metastases. Oncotarget.

11. Carlson CS, Emerson RO, Sherwood AM, Desmarais C, Chung MW, Parsons JM, et al.. Using synthetic templates to design an unbiased multiplex PCR assay. *Nat Commun*. Nature Publishing Group; 4:1–92013;

12. Amoriello R, Chernigovskaya M, Greiff V, Carnasciali A, Massacesi L, Barilaro A, et al.. TCR repertoire diversity in Multiple Sclerosis: High-dimensional bioinformatics analysis of sequences from brain, cerebrospinal fluid and peripheral blood. *EBioMedicine*. Elsevier; 2021; doi: 10.1016/j.ebiom.2021.103429.

13. Gupta NT, Heiden JAV, Uduman M, Gadala-maria D, Yaari G, Kleinstein SH. Change-O : a toolkit for analyzing large-scale B cell immunoglobulin repertoire sequencing data. 31:3356–82015;

14. Hill MO. Diversity and evenness: A unifying notation and its consequences. *Ecology*. Wiley; 54:427–321973;

15. Greiff V, Bhat P, Cook SC, Menzel U, Kang W, Reddy ST. A bioinformatic framework for immune repertoire diversity profiling enables detection of immunological status. *Genome Med*. Genome Medicine; 7:3–52015;

16. Curran J, Bolstad W. Bolstad: Bolstad functions.

17. Miho E, Roškar R, Greiff V, Reddy ST. Large-scale network analysis reveals the sequence space architecture of antibody repertoires. *Nat Commun*. 10:13212019;

18. Shannon P, Markiel A, Ozier O, Baliga NS, Wang JT, Ramage D, et al.. Cytoscape: a software environment for integrated models of biomolecular interaction networks. *Genome Res*. 13:2498–5042003;

19. Gillespie CS. Fitting Heavy Tailed Distributions: The poweRlaw Package. *Journal of Statistical Software*. 642015;

20. Amoriello R, Greiff V, Aldinucci A, Bonechi E, Carnasciali A, Peruzzi B, et al.. The TCR Repertoire Reconstitution in Multiple Sclerosis: Comparing One-Shot and Continuous Immunosuppressive Therapies. *Front Immunol*. 11:5592020;

21. Tickotsky N, Sagiv T, Prilusky J, Shifrut E, Friedman N. McPAS-TCR: a manually curated catalogue of pathology-associated T cell receptor sequences. *Bioinformatics*. 33:2924–92017;

22. Page DB, Yuan J, Redmond D, Wen YH, Durack JC, Emerson R, et al.. Deep Sequencing of T-cell Receptor DNA as a Biomarker of Clonally Expanded TILs in Breast Cancer after Immunotherapy. *Cancer Immunology Research*. 4:835–442016;

23. Beausang JF, Wheeler AJ, Chan NH, Hanft VR, Dirbas FM, Jeffrey SS, et al.. T cell receptor sequencing of early-stage breast cancer tumors identifies altered clonal structure of the T cell repertoire. *Proc Natl Acad Sci U S A*. 114:E10409–172017;

24. Sankin A, Chand D, Schoenberg M, Zang X. Human urothelial bladder cancer generates a clonal immune response: The results of T-cell receptor sequencing. *Urol Oncol*. United States; 37:810.e1-810.e52019;

25. Robert L, Tsoi J, Wang X, Emerson R, Homet B, Chodon T, et al.. CTLA4 blockade broadens the peripheral T-cell receptor repertoire. *Clin Cancer Res*. 20:2424–322014;

26. Wang X, Zhang B, Yang Y, Zhu J, Cheng S, Mao Y, et al.. Characterization of Distinct T Cell Receptor Repertoires in Tumor and Distant Non-tumor Tissues from Lung Cancer Patients. *Genomics Proteomics Bioinformatics*. 17:287–962019;

27. Liu J, Yang X, Lu X, Zhang L, Luo W, Cheng Y, et al.. Impact of T-cell receptor and B-cell receptor repertoire on the recurrence of early stage lung adenocarcinoma. *Exp Cell Res*. United States; 394:1121342020;

28. Casarrubios M, Cruz-Bermúdez A, Nadal E, Insa A, García Campelo MDR, Lázaro M, et al.. Pretreatment Tissue TCR Repertoire Evenness Is Associated with Complete Pathologic Response in Patients with NSCLC Receiving Neoadjuvant Chemoimmunotherapy. *Clin Cancer Res*. 27:5878–902021;

29. Lin K-R, Deng F-W, Jin Y-B, Chen X-P, Pan Y-M, Cui J-H, et al.. T cell receptor repertoire profiling predicts the prognosis of HBV-associated hepatocellular carcinoma. *Cancer Med*. 7:3755–622018;

30. Chen M, Chen R, Jin Y, Li J, Hu X, Zhang J, et al.. Cold and heterogeneous T cell repertoire is associated with copy number aberrations and loss of immune genes in small-cell lung cancer. *Nat Commun*. 12:66552021;

31. Reuben A, Zhang J, Chiou S-H, Gittelman RM, Li J, Lee W-C, et al.. Comprehensive T cell repertoire characterization of non-small cell lung cancer. *Nat Commun*. 11:6032020;

32. Bashford-Rogers RJM, Palser AL, Huntly BJ, Rance R, Vassiliou GS, Follows GA, et al.. Network properties derived from deep sequencing of human B-cell receptor repertoires delineate B-cell populations. *Genome Res*. 23:1874–842013;

33. Madi A, Poran A, Shifrut E, Reich-Zeliger S, Greenstein E, Zaretsky I, et al.. T cell receptor repertoires of mice and humans are clustered in similarity networks around conserved public CDR3 sequences. *Elife*. 2017; doi: 10.7554/eLife.22057.

34. Mullins CS, Linnebacher M. Endogenous retrovirus sequences as a novel class of tumor-specific antigens: an example of HERV-H env encoding strong CTL epitopes. *Cancer Immunol Immunother*. 61:1093–1002012;

35. Koesters R, Linnebacher M, Coy JF, Germann A, Schwitalle Y, Findeisen P, et al.. WT1 is a tumor-associated antigen in colon cancer that can be recognized by in vitro stimulated cytotoxic T cells. *Int J Cancer*. 109:385–922004;

36. Wagner S, Mullins CS, Linnebacher M. Colorectal cancer vaccines: Tumor-associated antigens vs neoantigens. *World J Gastroenterol*. 24:5418–322018;

37. Chiou S-H, Tseng D, Reuben A, Mallajosyula V, Molina IS, Conley S, et al.. Global analysis of shared T cell specificities in human non-small cell lung cancer enables HLA inference and antigen discovery. *Immunity*. 54:586-602.e82021;

Variable		N (%)	median (min-max)
Gender	Male	44 (52)	
	Female	41 (48)	
Age; years			65 (57-70)
Microsatellite stable cases		85 (100)	
Primary tumor location			
	Right colon	17 (20)	
	Left colon	30 (35)	
	Rectum	35 (41)	
Overall survival (from CLM surgery); months			56 (37-79)
NACT	No	35 (41)	
	Yes	50 (59)	
Interval NACT and CLM surgery; weeks	Short-interval group	35	7 (2.9-9.5)
	Long-interval group	15	16 (9.5-25.9)

Table 1. Clinical data for cohort used in this study.
Colorectal cancer liver metastasis (CLM), Neoadjuvant chemotherapy (NACT)

Figure Legends

Figure 1. Patient cohort and T cell fractions according of neoadjuvant chemotherapy (NACT) administration groups.

a. Overview of neoadjuvant chemotherapy (NACT) administration groups. We previously determined the NACT administration cut-off level to be 9.5 weeks based on receiver operator curve analysis[9]: The no-NACT group did not receive chemotherapy prior to liver resection, while the short- and long-interval groups received NACT 2.9 – <9.5 and ≥9.5 – 25.9 weeks prior to liver resection, respectively.
b. The mean T cell fraction was higher in the short-interval group than in the no-NACT group. P-values are from two-sided Welch t-test.

Figure 2. Hill diversity and evenness profiles according to neoadjuvant chemotherapy (NACT) administration group

a. Hill diversity profiles for 77 individual datasets by mean (solid line) with 95% confidence interval (shaded area), with q values between zero and ten.
b. Hill evenness profiles illustrated by mean (solid line) with 95% confidence interval (shaded area). The mean of the short- and long-interval groups were almost identical, with q values between zero and ten.
c. Boxplot of TCR repertoire clonality, defined by 10 – AUC of evenness profile. The middle bar denotes median clonality. P-values are from two-sided Welch t-test.
d. Linear regression of T cell clonality and the number of T cells (red line) shows a modest correlation between clonality and a high number of intratumoral T cells.

Figure 3 Network analyses

a. The network connectivity fraction (number of edges to number of nodes) plotted against the number of nodes. A linear regression model was fitted to the data (red

line), and the equation parameters are shown top left. The connectivity of the networks increased exponentially with the network size.

b. Plot of p-values from goodness of fit test to a power law function of TCR repertoire network degree distributions. A p-value greater than 0.1 was considered to indicate power law distribution.
c. Barplot of the number of clonal connections (degrees) against the number of patients in which a clone was detected. Error bars represent 95 % confidence interval.
d. Barplot of the percentage of public versus private clones across all datasets.
e. Comparison of network connectivity generated from TCR repertoires stratified into public and private clones, randomly subsampled to 1000 clones per network (because of the low abundance of public clones).
f. TCR repertoire similarity assessed using the Morisita-Horn index (MHI). MHI gives an estimate of the clonal similarity between two repertoires, weighted by clonal frequency, ranging from 0 (no similarity) to 1 (identical). The figure shows TCR repertoire similarity for the entire cohort, for datasets from different CLM from the same patient (n=4), and for datasets from analysis of two tissue aliquots from the same CLM (n=3).
g. Fraction of TCRs that were detected in the McPAS pathology associated TCR database. For each repertoire, TCR clones were stratified into two groups, the top 10% most highly connected (degree) and public (sharing level) clones, and the top 10% most expanded clones. The fraction was calculated from the number of clones with McPAS hits at Levenshtein distance of 0 in each group, divided by total number of clones among the top 10% in each group.

Figure 4 Visualization of representative CDR3β sequence connectivity networks.
Each node (dot) corresponds to a unique T cell CDR3β amino acid sequence, while connections are made between nodes when LD=1. The size of each node corresponds to its relative clonal abundance, while the colour scale represents the sharing level of the clone,

dark blue are clones unique to the individual patient, while yellow represents clones present across a large number of patients. a and b are networks selected from the short-interval (a) and no-NACT (b) groups. The two networks exhibit similar TCR parameters with respect to numbers of T cells and unique clones, as well as number of connections and connectivity fraction, but exemplify opposite ends of the clonality spectrum. (c) is a network from the short-interval group exemplifying a network with a very high number of T cells and unique clones.

Figure 1

Click here to access/download;Figure;fig1.pdf

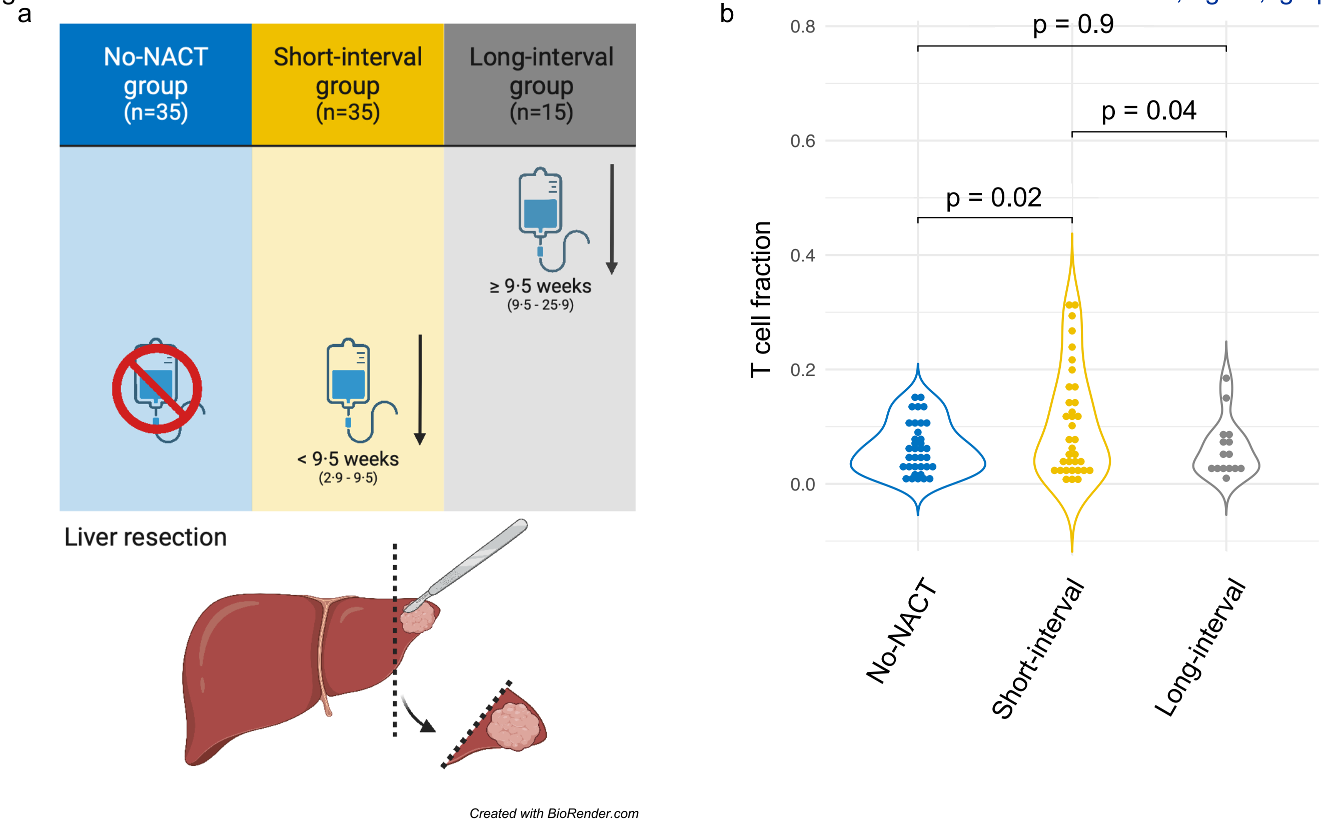

Figure 2

Click here to access/download;Figure;fig2.pdf

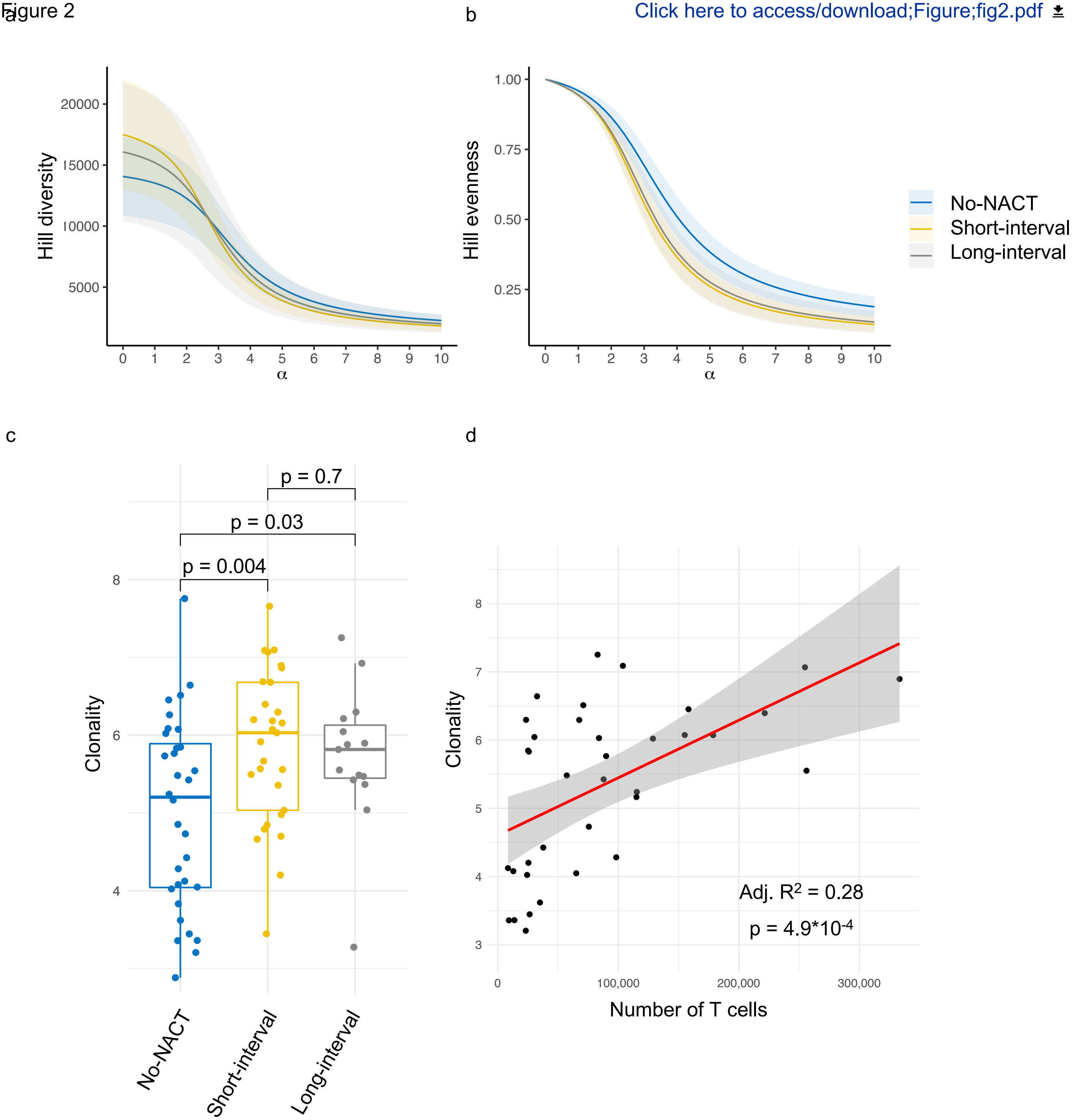

Figure 3

Click here to access/download;Figure;fig3_v2.pdf

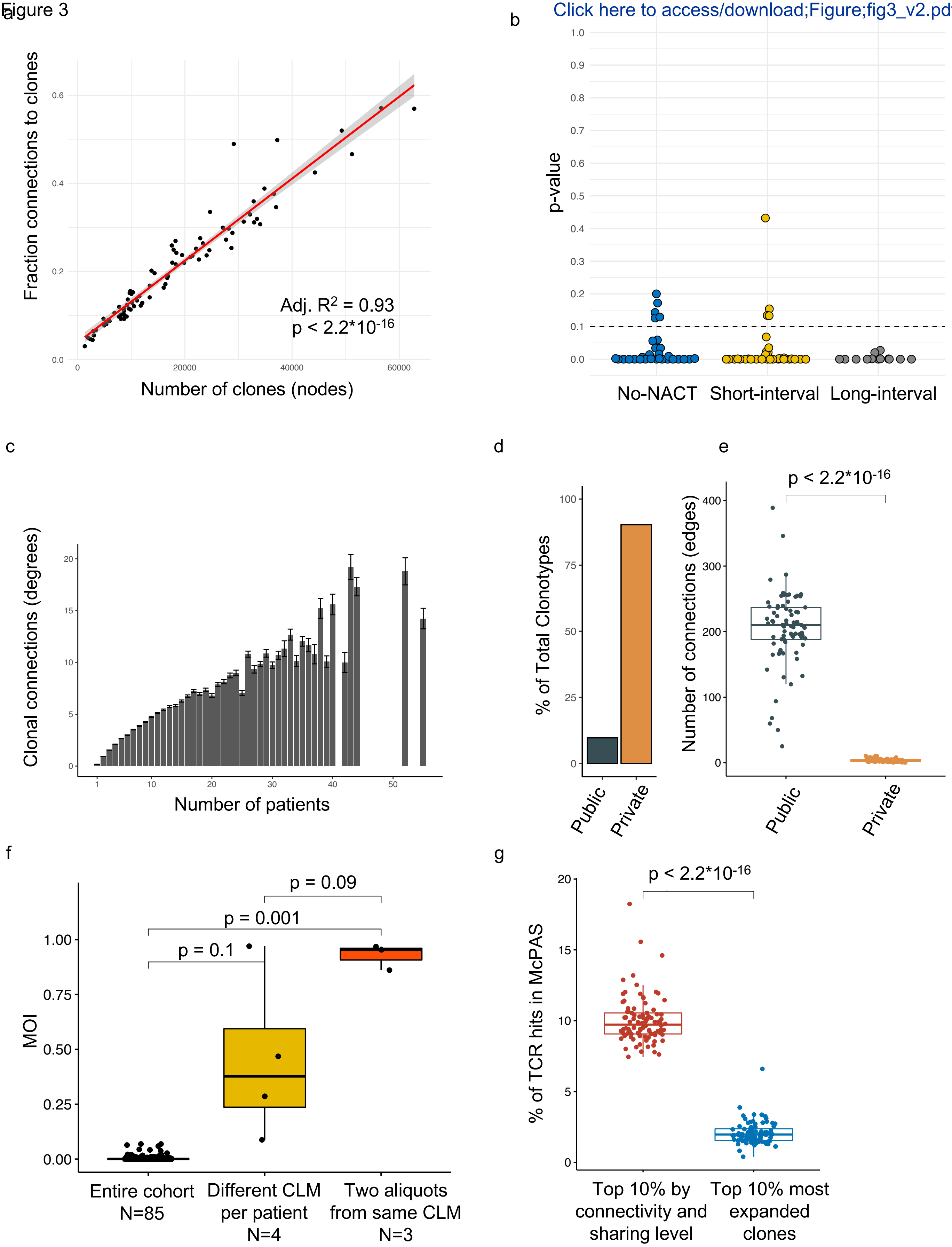

Figure 4

Click here to access/download;Figure;fig4.pdf

a

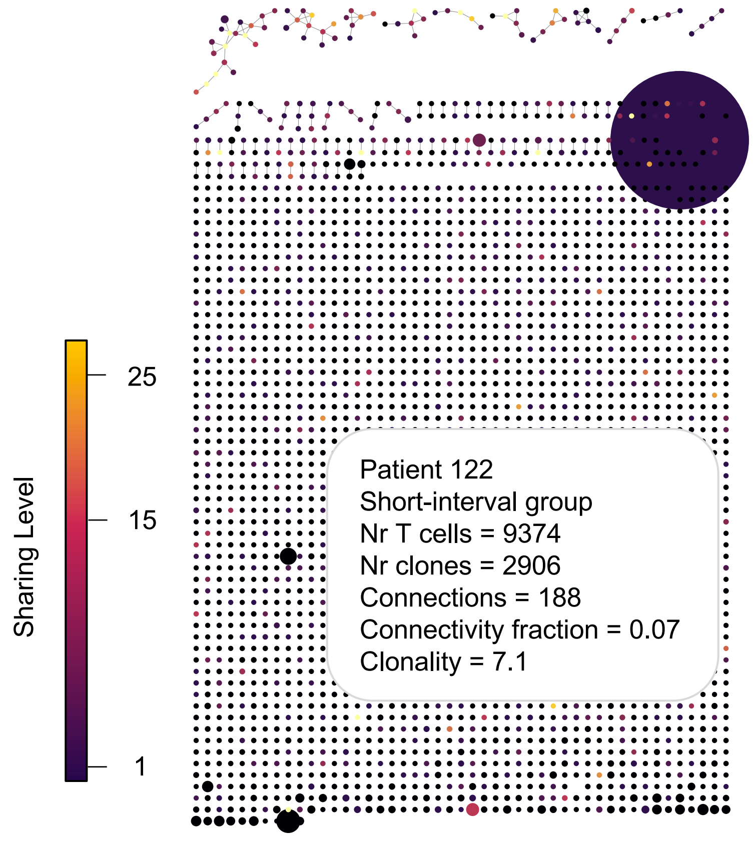

b

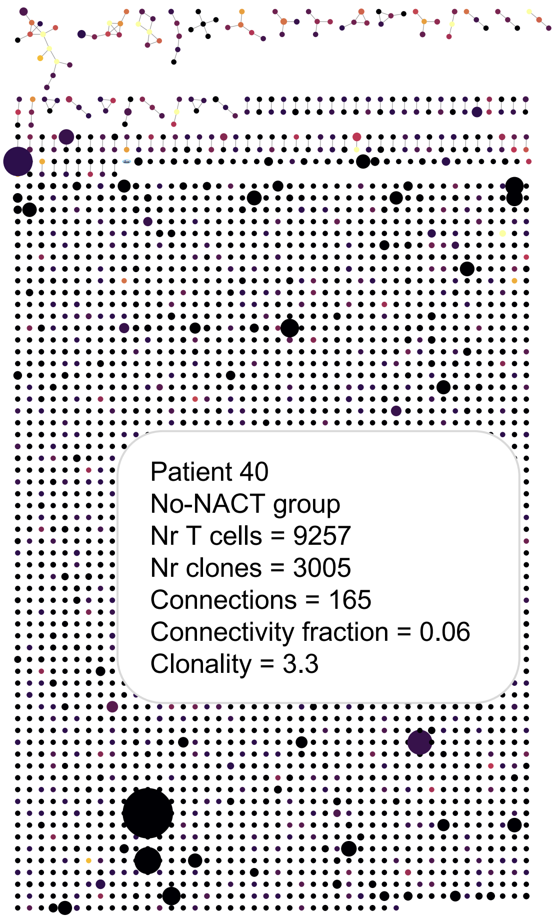

c

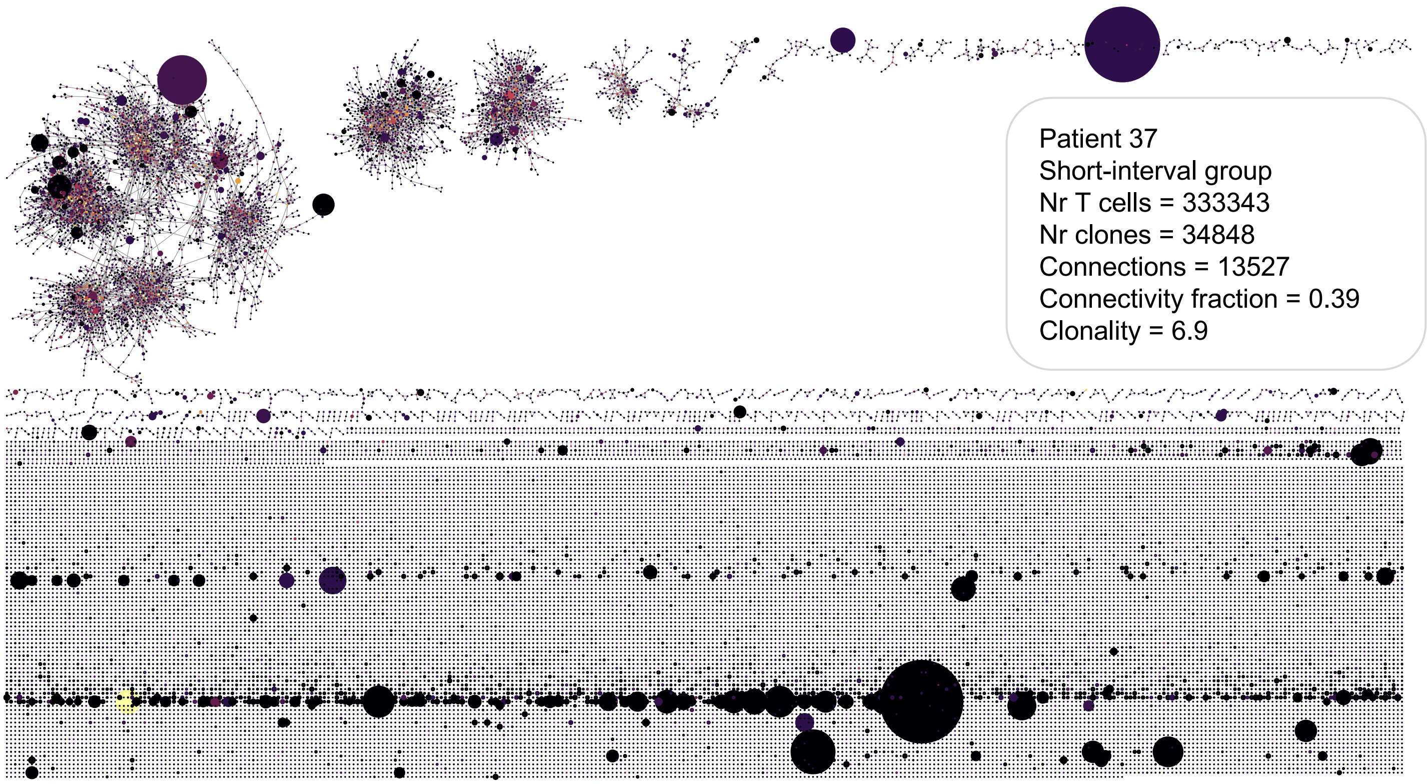

R markdown figures and statistical analysis

Click here to access/download
Supplementary Material
figure_r_code_v3.html

Supplementary File

Click here to access/download
Supplementary Material
Supplementary_file.docx

Cover letter
Click here to access/download;Personal Cover;Cover letter.docx

Oslo University Hospital

Norwegian Radium Hospital
Oslo University Hospital
Postal address:
Trondheimsveien 235
NO-0514 Oslo
Norway

Switchboard:
+47 915 02770

Org.number:
NO 993 467 049 MVA

www.oslo-universitetssykehus.no

Oslo, September 28, 2022

The Editorial Office,
GigaScience

Dear Editor,

Please consider the enclosed manuscript entitled "T cell receptor repertoire sequencing reveals chemotherapy-driven clonal expansion in colorectal liver metastases" by Høye and co-authors for publication in *GigaScience*.

Cytotoxic chemotherapy is hypothesized to induce immunogenic cell death (ICD), causing immune activation, but evidence from the clinical setting is very limited. Colorectal cancer is typically a non-immunogenic cancer with poor responses to immune checkpoint inhibition (ICI); yet, T cell infiltration has been shown to be positively correlated with overall survival and response to immunotherapy. Our group previously identified a transient increase in tissue T cells in colorectal liver metastasis (CLM) samples from patients exposed to neoadjuvant chemotherapy (NACT).

In this work we sequenced and characterised T cell receptor (TCR) repertoires in colorectal liver metastasis (CLM) resection samples from 85 patients. Analysis of TCR repertoires again suggested a transient increase in T cell infiltration after NACT administration, but at the same time, a persistent increase in clonality was observed. These findings are consistent with a chemotherapy-driven clonal expansion of T cells, possibly because of ICD and exposure to tumour associated antigens. Being one of very few studies that is based on analysis of clinical samples, this work therefore represents an important contribution to understanding the role of chemotherapy in immune activation in metastatic colorectal cancer. The results furthermore reemphasise why timing is an important consideration when combining administration of ICI and chemotherapy in clinical trials, and point to TCR sequencing as a powerful tool for analysis of T cell infiltration and clonality in cancer research.

Because the topic is of high relevance, we hope that the manuscript will be of interest to the readership of *GigaScience*. The use of open science practices in this work facilitates reproducibility, and the new datasets will be fully accessible to researchers for integration into future research projects. We are looking forward to your response.

Yours sincerely,

Kjersti Flatmark, MD, PhD, (corresponding author)
Professor of Surgery
Research Group Leader, Department of Tumor Biology
Consultant Surgeon, Department of Gastroenterological Surgery
