# Supplementary material for: T cell receptor repertoire sequencing reveals chemotherapy-driven clonal expansion in colorectal liver metastases
Source: Gigascience. 2023 May 10;12:giad032. doi: 10.1093/gigascience/giad032 (PMC10170408; doi:10.1093/gigascience/giad032)
Supplement: giad032_GIGA-D-22-00270_Revision_1 [file giad032_giga-d-22-00270_revision_1.pdf]

GigaScience

T cell receptor repertoire sequencing reveals chemotherapy-driven clonal expansion in colorectal liver metastases

--Manuscript Draft--

Manuscript Number:	GIGA-D-22-00270R1
Full Title:	T cell receptor repertoire sequencing reveals chemotherapy-driven clonal expansion in colorectal liver metastases
Article Type:	Research
Funding Information:	Helse Sør-Øst RHF (2018014) — Professor Kjersti Flatmark<br>Kreftforeningen (215817) — Dr Victor Greiff
Abstract:	Background<br><br>Colorectal liver metastasis (CLM) is a leading cause of colorectal cancer mortality, and the response to immune checkpoint inhibition (ICI) in microsatellite stable CRC has been disappointing. Administration of cytotoxic chemotherapy may cause increased density of tumour infiltrating T cells, which has been associated with improved response to ICI. This study aimed to quantify and characterize T cell infiltration in CLM using T cell receptor (TCR) repertoire sequencing. Eighty-five resected CLM from patients included in the Oslo CoMet study were subjected to TCR repertoire sequencing. Thirty-five and 15 patients had received neoadjuvant chemotherapy (NACT) within a short or long interval, respectively, prior to resection, while 35 patients had not been exposed to NACT. T cell fractions were calculated, repertoire clonality was analysed based on Hill evenness curves, and TCR sequence convergence was assessed using network analysis.<br><br>Results<br><br>Increased T cell fractions (10.6% vs 6.3%) were detected in CLM exposed to NACT within a short interval prior to resection, while modestly increased clonality was observed in NACT exposed tumours independently of the timing of NACT administration and surgery. While private clones made up >90% of detected clones, network connectivity analysis revealed that public clones contributed the majority of TCR sequence convergence.<br><br>Conclusions<br><br>TCR repertoire sequencing can be used to quantify T cell infiltration and clonality in clinical samples. This study provides evidence to support chemotherapy-driven T cell clonal expansion in CLM in a clinical context.
Corresponding Author:	Kjersti Flatmark, PhD<br>Radiumhospitalet Institutt for kreftforskning: Oslo Universitetssykehus Institutt for kreftforskning<br>Oslo, NORWAY
Corresponding Author Secondary Information:	
Corresponding Author's Institution:	Radiumhospitalet Institutt for kreftforskning: Oslo Universitetssykehus Institutt for kreftforskning
Corresponding Author's Secondary Institution:	
First Author:	Eirik Høye
First Author Secondary Information:	
Order of Authors:	Eirik Høye

Powered by Editorial Manager® and ProduXion Manager® from Aries Systems Corporation

	Vegar Johansen Dagenborg
	Annette Torgunrud
	Christin Lund-Andersen
	Åsmund Avdem Fretland
	Susanne Lorenz
	Bjørn Edwidn
	Eivind Hovig
	Bastian Fromm, PhD
	Else Marit Inderberg, PhD
	Victor Greiff, PhD
	Anne Hansen Ree, PhD
	Kjersti Flatmark, PhD
**Order of Authors Secondary Information:**	

Response to Reviewers:

Reviewer reports:

Reviewer #1: In this study Høye describe an analysis of the T cell receptor (TCR) repertoires of tumours from patients with colorectal liver metastases (CLM), who received different neoadjuvant chemotherapy (NACT) regimes. Their results agreed with their prior results from another cohort with orthogonal techniques: it appears that a short-term treatment (<9.5 weeks) associates with a increased T cell infiltration relative to longer intervals, and that repertoires of NACT-treated tumours in general showed greater clonality, indicative of clonal expansion.

It is an extremely well written and plotted manuscript, employing sensible and robust analyses on a reasonably-sized cohort of patients. Use of the T cell fraction in particular is very appealing potential biomarker that could be used in the adoption of repertoire sequencing for a greater range of clinical conditions, while still supplying the additional information repertoires bring. The data and code were also made available to the reviewers, and there's every indication that these will be suitably available upon publication. I am very happy recommending its publication, as I think it will be of use to the field. I have noted a small number of areas that caught my attention for the authors' consideration: several are places where some additional plotting may help reduce the possibility that the results are confounded, while the rest are minor typographical comments or similar.

Reply:
We extend our heartfelt gratitude to Reviewer 1 for the excellent and very thorough review, and for the constructive comments that improved the manuscript. See below our replies to this very detailed and excellent review.

1) I have a couple of queries about the T cell fraction. While none are major, given the importance of the metric to the study these are probably worth addressing:

a. L143 states that the number of unique CDR3b sequences is used in its calculation. Could the authors please clarify in the text to specify whether different recombinations (potentially using different V/J genes) producing the same CDR3s are grouped together in these calculations?

Reply:
In the event of different V/J gene recombination resulting in the same rearrangement sequence, these were not grouped together, and counted separately. T cell fraction was calculated by the sum of rearranged DNA templates detected in each sample (a proxy for the number of T cells detected), divided by the total number of genomes (calculated from the amount of input gDNA in the first library preparation mix). Counting the same sequential rearrangement produced from different V/J gene rearrangements separately or together will therefore not make a difference on the T cell fraction

calculation. The lines 144-146 have been adjusted to make this clearer.

b. Given that the T cell fraction is calculated using the number of TCR rearrangements per sample, there's a chance that it might be confounded by differences in sequencing depth or errors, which are both in part dependent on the amount of input DNA. To rule these possibilities it might help the reader to include plots showing a lack of correlation between T cell fraction and number of productive TCR rearrangements, and between T cell fraction and amount of input gDNA. Additionally randomly selecting a matched number of sequences per donor prior to metric calculation can help rule out the possibility that these variables might be confounding (similar to how higher q values remove the effects of small clones in the later diversity metrics).

Reply:
Regarding T cell fraction and sequencing depth, all samples were sequenced with the same number of PCR replicates, which is what determines sequencing depth. Sequencing coverage in the Adaptive Biotechnologies ImmunoSEQ platform is calculated as the average number of reads per rearranged DNA template (proxy for the number of T cells) prior to PCR amplification. This number is possible because of the presence of synthetic rearrangements diluted to be present at either 0 or one template in library preparation mix prior to PCR amplification. Sequencing coverage is therefore expected to decrease as the number of T cells present in the tissue increases.

The below plots show the relationship between coverage, input gDNA in the library preparation mix and T cell fraction.

Points were colored based on whether the T cell fraction was greater than 0.15. Dashed lines represent sequencing coverage of 5.

a.Samples with very high T cell fraction leads to low sequencing coverage.
b.There was no correlation between T cell fraction and input gDNA.
c.Nor was there a strong correlation between gDNA and coverage.

All biological samples were run using two PCR replicates. The ImmunoSEQ protocol is optimized for 30 000 to 45 000 rearranged T cell genomes per PCR replicate. The median number of productive rearranged DNA templates (proxy for number of T cells) per PCR replicate in this dataset was 31 532, well within the optimal range. However, among the samples with high T cell fraction, the number was much higher, the maximum being 166 672 rearranged templates. The low coverage for these samples seen in plot a was likely caused by oversaturation of T cells. Ideally, the input DNA in these samples would have been reduced prior to library preparation, so as to have the optimal T cell number per PCR replicate. However, the T cell content for each tissue was not known prior to sequencing. As can be seen from plot b, T cell fraction was not impacted by variation in input gDNA, nor was coverage (plot c).

For T cell fraction analysis, the lower coverage and oversaturation of T cells for CLM tissue with very high T cell content is not of major concern. Potential bias likely reflects an underestimation. However, for clonality analysis low coverage is a major concern, resulting in a bias towards abundant clones. Therefore, samples with low coverage (< 5) were dropped from the downstream clonality analysis in order to ensure unbiased analysis.

The below plot show that there was no association between clonality and coverage.

Linear regression (clonality ~ coverage) intercept = 5.74; slope = -0.015 (95% CI: -0.039 – 0.009); p-value = 0.2. Clonality was evenly distributed over the coverage distribution, and the slight negative trend in clonality for increasing coverage was likely random noise. This shows that Hill evenness based clonality estimates are robust both to variation in absolute T cells, and also robust to variations in sequencing coverage.

These figures have been added to the supplementary file. Lines 135-136 of the

Powered by Editorial Manager® and ProduXion Manager® from Aries Systems Corporation

manuscript have been updated to make the rationale for input gDNA per PCR replicate clearer.

c. Re: Fig 1: while the range of interval length is given, the distribution is not. I wonder if perhaps a (potentially supplementary) plot of duration of interval vs T cell fraction would be instructive. This would also be a potentially independent useful validation of the use of 9.5 mo as a threshold, which it seems was determined by the authors in a significance screen by ROC analysis in their prior publication. As the discussion remarks on the agreement between the results of this study broken by interval length and that previous publication, the authors may also wish to consider repeating that ROC analysis on these data, to see if a similar threshold is produced.

Reply:
We agree that it would be valuable to have an independent validation of the 9.5 months cutoff, however the material produced in this study is not suitable for this task. Samples were selected with the purpose of acquiring sufficient samples in each NACT-interval group for meaningful statistical analysis. The time interval distribution is not independently sampled, and would not be suitable for statistical analysis. The minimum and maximum interval in weeks for the two groups was already described on L117, but we have also added the median.

2) The Hill diversity/evenness data beautifully plotted in Fig 2 a/b is a robust way to illustrate these 'species'-level metrics.
a. However in my experience it can't be expected that the reader will know how to interpret these plots: even many people who study repertoire data are unfamiliar with these more ecological measures. I imagine this is perhaps even more true of the oncologists who may be interested in this study. I wonder if perhaps an explanatory sentence or two in the legend might help? I know there is some of this in the discussion, but that's likely going to be seen after the figures for many readers.

Reply:
Additional explanatory text has been added to the relevant legends. Updated figure 2 a and b legend text.

b. Also I think maybe the x axes would more accurately be labelled 'q values' (rather than 'α').

Reply:
This is correct, we apologize for this mix-up. We have altered the x axis label to q. Figure 2 a and b have been updated.

d.Some other values can be guessed at but aren't specifically defined in the legend, e.g. it only defines the median for Fig 2c and not the whisker/boxes, while the shaded confidence interval area in 2d is similarly not defined. It's also unclear where the T cell count information is coming from in this plot: were orthogonal T cell count data collected, or is this inferred from the repertoires?

Reply:
The relevant figure legends have been updated to include these points. Figure 2 d legend text has been updated.

3) The network analyses of Fig 3/4 are interesting, but currently presented in a way was harder to intrepret than surrounding sections, with several fairly field-specific analyses done with little explanation.
a. The text results of this analysis (beginning on L257. "The mean number of nodes detected was...") is abrupt, discussing results of an analysis that hasn't been established yet. This continued through the paragraph. E.g. it's unclear why the test for power law was performed: while it is well known that clonal repertoires tend to follow such distributions, the authors don't state why they think the network connectivities should.

Reply:

This is a fair point, and we agree that this section may be somewhat abrupt. Our rational for this analysis was to see whether there may possibly be a convergence of TCR sequences based on shared specificity of antigen epitopes. However, in this case there was no evidence for such a convergence. We have updated the text (L259 – L261). We have also added a sentence on the background for why we conducted a test for whether the degree distribution followed a power law distribution. We have updated the text (L266 - L268) to make the purpose and our prior expectations clearer.

b. Following on from my point (1) above, a common confounder in such connectivity networks is the possibility for sequencing and PCR errors to artificially inflate networks. The incidence of such errors could be biased towards certain samples, depending on factors like gDNA input and number of PCR cycles. You would also expect more abundant clonotypes on average to produce greater numbers of erroneous sequences, as a function of their occupancy of the reactions involved, which could contribute to the correlation seen in Fig 3a. Some control analyses or discussion might be useful, to help discount this possibility (with other plots in this figure lending weight to the notion that this is not artefactual).

Reply:
This is a fair point, and we have added another sentence explaining why these sequence convergences occur (L388-390). However, this does not alter the primary conclusion of this manuscript. The sequence convergence in the network analysis were mainly composed of singleton or low abundance clones. Expanded clones were, in contrast, sequentially distinct and disconnected from the other clones in the network analysis, as can be seen in figure 4. The finding from this study therefore suggest that the T cell infiltration is driven by a heterogenous neoantigen landscape, unique to each patient/tumor.

c. Again there are some missing value definitions: 3a shaded area, box/whisker markers in e-g. The 3f y axis label is also mislabelled: 'MOI' should likely be 'MHI'.

Reply:
Figure 3 legend have been updated to include these definitions, and Figure 3f axis label has been changed to MHI. We thank the reviewer for making us aware of these omissions.

4) It is also probably worth noting somewhere in the text that the connectivity difference between public/private sequences is exactly what one would expect given our understanding of biased V(D)J recombination (which underlies the production of public sequences in the first place). https://doi.org/10.1073/pnas.0608907103 would be a classic citation for this, with more recently validations using higher-throughput TCRseq data discussed in https://doi.org/10.1111/imr.12665.
a. It has also similarly been observed that many T cells detectable in tumours are specific for viruses in both humans and mice, e.g. https://doi.org/10.1038/s41586-021-03704-y, https://doi.org/10.1172/JCI150535, and https://doi.org/10.4049%2Fjimmunol.1601064.

Reply:
As mentioned above, we have added another sentence (L388-390) that makes this point, and included the mentioned references. Furthermore, the results from the analysis of TCR sequences present in the McPAS database revealed that the most common pathological associations included influenza, tuberculosis, cytomegalovirus and epstein barr virus. This has already been mentioned in the text. In regards to the conclusion, we think that it is significant that, as can be seen in fig3g, the most expanded clones also had fewer hits in the McPAS database. This suggests these expanded clones are likely specific to private neoantigens.

5) As noted above, both the data and code are available to the reviewers, which is great. However there are a few things that could be changed to align more with the reproducibility and data reuse this journal prides itself on.
a. The Github repo seems to have had a working directory uploaded to it, along with the associated detritus that such folders generate. There are several scripts and tables that seem to be derived from each other (eg X, X_v2), and at least one directory of what I suspect is analyses that didn't make it to the manuscript ('gliph2'). Tidying up

Powered by Editorial Manager® and ProduXion Manager® from Aries Systems Corporation

these redundancies and not needed files would make the repo more navigable.

Reply:
We have tidied up the repository to make it easier to navigate for other researchers. Metadata files have been merged and cleaned up, and a more fleshed out README.md file should make the repository more navigable.

b. Similarly, while many of the scripts are sensible named, commenting is sparse and there's no summary or record of what metadata is recorded where and what scripts do what. While I'm sure the researchers running the analyses know exactly how to navigate this repo, anyone wishing to repeat or reapply these analyses will not, so an expanded README or similar would help a lot. This would be especially useful for the core pipeline of the study, and the key metadata values (e.g. Adaptive sample ID, patient IDs for those with >1 sample, treatment status/interval, ng DNA etc).

Reply:
The README.md file in the repository has been fleshed out to make it clearer. All metadata has been cleaned up so that there are now only three files:
-SampleOverview.tsv (Sequencing metadata and input gDNA)
-metadata.xlsx (clinical metadata and also show which samples were from patients sequenced more than once)
-qcReport.tsv (mainly for sequencing coverage)

c. It looks like the journal's policies call for DOIs for code as well as data: I don't know about their provided Code Ocean option, but generating a Zenodo DOI for a GitHub release is very straightforward, and makes sense seeing as the code is already there.

Reply:
This is a great suggestion; we have generated a Zenodo DOI to the github repository:
DOI: 10.5281/zenodo.7614598
6) A few of the axis tick label fonts are small relative to the rest of the figure (e.g. Fig 3a/c), so making these larger would help the short sighted readers like myself from having to frequently zoom in.

Reply:
The font sizes in Fig 3a/c have been increased.

7) L202-204, sample exclusion. It's not entirely clear to me what "sequencing coverage <5" means here: fewer than five different TCRs, or counts, or something else? In any case it should be stated which of the three test arms (no-NACT/short/long) they fell from.

Reply:
Sequencing coverage was calculated as the mean number of reads per rearranged DNA template in the original library prep mix prior to PCR amplification (which was used in this study as the proxy for number of T cells, as described above). This number can be calculated from post PCR sequencing data because each library preparation mix also contain synthetic rearranged DNA templates diluted to be present at either 0 or 1 template. This allows one to count backwards the number of DNA templates present in the original mix. A general recommendation is that, at a minimum, the number of sequencing reads should exceed the clonal diversity (1). Clonal frequency distribution for all sequencing datasets in this study were at least somewhat skewed, therefore the clone to cell ration was below 1 for all samples. The <5 coverage cut-off was the recommendation from consulting with Adaptive Biotechnologies. Because it is calculated based on reads per cell, not based on reads per clone, it is well above the minimum sequencing depth recommendation. L208 has been updated to include n for each group.

8) L183: the version/accession date of when McPAS was used should be stated. It might even be worth depositing the version of the database that was used somewhere accessible (e.g. the analysis repo), as since Nir Friedman's sad passing it appears that the resource's original URL is no longer working

Powered by Editorial Manager® and ProduXion Manager® from Aries Systems Corporation

Reply:
This is a good suggestion, we have added the file used to query to the github repo, and have added the download date on lines L185-L186.

9) Use of the word 'monoclonal' in L287-288 ("...a very large monoclonal network (Clonality=6.9) is visualized...") seems counter-intuitive, seeing as the sentence later points out how there are tens of thousands of different CDR3s are involved.

Reply:
We have re-phrased some of the sentences in this section to make it clearer. L292, L295 and L298 have been edited.

10) L254, possible typo or unclear abbreviation: "median og y"

Reply:
Thank you for making us aware, we apologize for this mistake and have corrected the typo.

11) Several times 'sequential' is used to mean 'relating to sequences'. I think most people will be able to figure out what this means, but (at least in my specific dialect of English) 'sequential' is mostly only used to describe things which occur in a sequence, rather than describe the sequences in the abstract themselves, so this threw me each time it came up. Changing to 'sequence' (e.g. L41/42, "TCR sequential convergence" could become "TCR sequence convergence") would avoid this if the authors wished.

Reply:
L41 has been changed to TCR sequence convergence.

Reviewer #2: This manuscript by Høye et al analyzed the effects of NACT on the TCR repertoire of TIL in CLM. Using several unique approaches of data analysis, they concluded that NACT increased the T cell infiltration and clonal expansion. Overall, data and conclusion seems to be convincing. The results that "shared clones have higher connectivity of TCR sequences" and "shared clones are enriched with antigen-specific TCRs registered in the database" were novel and interesting. On the other hand, as the authors point out in the discussion, there have been similar studies on other types of cancer, and the "increase in clonality with neoadjuvant chemotherapy" is not novel. It was difficult for me to understand the contents of the Results because the indicators used to evaluate the repertoire were not popular. I think it would be better to move some of the information on the significance of each indicator and the purpose of the analysis from the Discussion to the Results, and make the Results easier to read.

Reply:
We thank reviewer 2 for taking the time and effort to review our manuscript. Both reviewer 1 and 2 have suggested to add more explanatory text to the results section. We have added sentences to lines L259-L261 and L266-L269 in order to make the purpose of the network analysis clearer, as well as expanding the fig 2a and 2b figure legends so that it is easier to interpret for readers who are not familiar with Hill diversity and evenness profiles.

Major points
1. Line 247-248: clonality is evaluated using AUC of Hill diversity profile. However, since the "1-Pielou" index is commonly used in studies on TCR repertoire analysis, it would be better to show the calculation results of 1-Pielou index as a Fig.

Reply:
While many studies on TCR repertoire analysis use 1-Pielou index, the Hill diversity profiles, in a sense, already contain the Pielou index. This is because the Hill function at q=1 is mathematically equivalent to the reciprocal of Shannon entropy, as was proven by Hill in 1973. Pielou index is derived from Shannon entropy, thus is included in the diversity profile in fig 2a. The reason for using the whole profile is because different diversity indexes can yield qualitatively different answers on which repertoire

is the most diverse, thus one should assess them based on a range of diversity estimates, such as that shown in fig2a. The AUC based clonality estimate was primarily intended for making a convenient single point estimate for statistical comparison, but one should consider the entire profile when evaluating clonality, i.e. all of fig2a, b, c.

2. line 238-241: Hill diversity profile was not explained, and it was difficult to understand. It would be helpful to include the information equivalent to Discussion line302-309.

Reply:
This was also a request from reviewer 1, and we have added some additional explanatory sentences in the figure legends. Figure legend 2a and 2b have been extended to make it easier to interpret for readers who are not familiar with Hill diversity and evenness profiles.

3. Fig. 2 claims an "increase in Clonality with neoadjuvant chemotherapy". Although this study did not separate CD4+ T cells from CD8+ T cells in the TCR repertoire analysis, it has been reported that CD4+ T cells have higher Clonality than CD8+ T cells in their repertoires (Gueguen et al. 2021, Zhang et al. 2018). Therefore, the increased Clonality of tumor T-cell repertoires reported in this study may only reflect an increased proportion of CD8+ T cells in tumor-infiltrating T cells (Rudqvist et al. 2018). This point should be mentioned as a limitation in Discussion.

Reply:
This is a fair point, and we have added a sentence to the limitations part of the discussion, L394-L395. We agree that this is a limitation, however Dagenborg et al, 2020 (2) found that, using immunohistochemistry on a subset of the same cohort, both CD4+ and CD8+ T cells had higher infiltration in the short-interval group compared to the no-NACT and long-interval group.

4. Line 257-259: I couldn't figure out why the authors performed network analysis just by reading the result. Please include something like Discussion line 359-363 in the result.

Reply:
This was also requested by Reviewer 1, we have expanded this section to explain the purpose of these analysis, L259-L261.

5. Line 278-279: Please provide a table with a list of target pathogens (flu, tuberculosis, etc.) of the clones that matched the McPAS database. Also, please indicate what type of cancer antigens (differentiation antigen, cancer testis antigen, neoantigen, etc.) are listed as "colorectal cancer".

Reply:
The table of unique CDR3 amino acid hits to the McPAS database, for both the public and private most prevalent clones, have been added to the github repository:
-data/McPAS/public_cdr3_aa_unique_McPAS_hits.csv
-data/McPAS/private_cdr3_aa_unique_McPAS_hits.csv

Regarding McPAS, it is a database of TCR sequences that have been reported in the literature to be associated with pathological conditions. The specific antigens they respond to is not necessarily known. Our understanding of TCR to antigen specificities is limited, due to the vast diversity of both TCR sequences, and potential tumor neoantigens. Exploring TCR:antigen specificities is outside the scope of this work, and not currently possible with sequencing technology alone.

6. Line 281-290: Fig. 4 only shows an example of TCR network, so I felt that the order of presentation should be switched with Fig. 3.

Reply:
While we can understand this point of view, the information in Fig. 3 is essential for understanding Fig. 4. We therefore think the order is correct.

7. Line 340-343: "differences in clonality between NACT exposed and unexposed CLM might be of clinical relevance." I was not sure what this "clinical relevance" meant. Also, if the authors are trying to claim similarity of their tumor microenvironment based solely on the similarity between "differences in TCR repertoire index between immune cold SCLC and immune hot NSCLC" and "differences in TCR repertoire index of CLM with and without NACT", it is an overstatement.

Reply:
We believe that the difference in clonality may reflect differences in response to chemotherapy. This may be important in the context of immunotherapy in mCRC. This hypothesis, where priming the immune system with chemotherapy in a brief time interval prior to immunotherapy, is currently being tested in the randomized control trial METIMMOX (NCT03388190). Regarding the analysis of clonal overlap between repertoires, our findings (Fig. 3f) showed that there was no clonal overlap between patients, while repertoires from the same patient but sampled at different locations of metastasis in the liver showed only modest overlap. Repertoires generated from the same metastatic aliquots had almost complete clonal overlap. Our conclusion from this is again that this is suggestive of a CLM neoantigen landscape that is highly diverse and private to the individual patient. NSCLC in contrast had far higher intratumoral TCR homogeneity, suggesting the neoantigen landscape of these tumors is shared for the majority of tumor cells. This is an important predictor for whether current immunotherapy approaches will have an effect, and may be part of the reason for the very good response in NSCLC compared to the poor response in SCLC and also in mCRC. We agree however that one must be careful comparing these studies, due to possible differences in sampling strategies, etc.

8. Line 352-355 compares the heterogeneity of TCR repertoires in tumors between NSCLC and SCLC, which seemed not relevant to the content of our paper.

Reply:
We think this is interesting in the context of potential response to immunotherapy. The more homogenous TCR repertoire in NSCLC is suggestive of a homogenous neoantigen landscape, which is an important predictor of response to immunotherapy. See also reply above.

Minor points
1. line 242: I do not understand what "increasing value of q" refers to. Is it referring to the "alpha" in Fig2 a, b?

Reply:
We apologize for this mistake, which was also pointed out by reviewer 1. The Fig. 2a and 2b x axis label have been fixed to q instead of alpha.

2. In Fig. 2b, the plot for the treated group was lower than the plot for the untreated group, but I did not immediately understand the correspondence between this and the statement "Clonality was higher in the treated group". Legend stated that "Clonality was evaluated by 10-AUC", which I think should be clearly stated in the RESULT.

Reply:
The evenness profile curves should be read as follows. If the curve follows a straight line (evenness close to 1.0) for the entire range of q values, the clonal frequency distribution is completely even (all clones are identical at the same frequency, a completely heterogenous distribution). The further and more rapidly the curve drops as the value of q increases, the more uneven the clonal frequency distribution. (i.e. the more it is dominated by a small subset of very frequent clones). Legends for Fig2a and 2b have been updated, as was also requested by reviewer 1. The 10-AUC is explained in the methods section, L157-L161.

3. line 250-251: "regression analysis revealed a modest association between a high absolute number of T cells and clonality," I could not understand what this analysis was intended to claim.

Reply:
We included this because it suggests that the higher number of infiltrating T cells is

Powered by Editorial Manager® and ProduXion Manager® from Aries Systems Corporation

driven by clonal expansion.

4. Lines 259-260: I didn't understand what you were trying to argue with this analysis.

Reply:
We have added some explanatory text that explains the purpose of the network analysis. See L259 – 261, this was also requested by reviewer 1.

5. Line 262-264: The authors compared the clonality between repertoire that satisfy the power-law fit test and those that do not. Please provide a dot plot in addition to the average value.

Reply:
While we can understand why an additional plot might be useful, the take home message from the power law was that the networks produced in this study did not follow a power law. Prior studies on antibodies have found that the degree distribution of neoantigen experienced networks follow power law, and our rationale for conducting this analysis was that this might have been an exiting tool for assessing whether the repertoire is antigen experienced or not. However, it appears that T cell repertoires follow different dynamics, and the results of this analysis was negative.

6. Line 264-265: It is an overstatement to conclude that “clonally expanded datasets were not associated with power law degree distribution” because there are only a few repertoires that satisfy the power-law fit test.

Reply:
As also explained above, the overall conclusion from this analysis was that TCR repertoire networks do not follow the same dynamics of antibody repertoires, and power law test are, regrettably, not a suitable tool for assessing if a repertoire is antigen experienced, in this context.

7. Line 268-270: Fig3d might be a mistake for Fig3e. Also, Fig3e does not seem to be a graph comparing "mean connectivity fractions".

Reply:
Again, we apologies for the error in figure reference, which have been corrected. Regarding Fig3e, the repertoires were randomized to 1000 clones each, therefore the numbers are equivalent to fractions which is written in the text.

8. line 270: Fig3e" seems to be a mistake for "Fig3f".

Reply:
Again, we apologies for the error in figure reference, which have been corrected.

9. Line 270-271: "in line with", but I did not understand how the discussion of public clone connectivity corresponds to the discussion of repertoire similarity between samples.

Reply:
We have rewritten this line to instead say “further illuminated by”, to make our intension clearer, L280.

10. Spelling error: line 68 "Characterisation"

Reply:
We have changed this word to characterization.

11. Fig3f, Y-axis seems to be MHI.

Reply:
Corrected, as was also mentioned by reviewer 1.

	References:<br><br>1. Greiff V, Miho E, Menzel U, Reddy ST. Bioinformatic and Statistical Analysis of Adaptive Immune Repertoires. Trends Immunol. 2015;36(11):738–49.<br>2. Dagenborg VJ, Marshall SE, Yaqub S, Grzyb K, Boye K, Lund-Iversen M, et al. Neoadjuvant chemotherapy is associated with a transient increase of intratumoral T-cell density in microsatellite stable colorectal liver metastases. Cancer Biol Ther. 2020 May 3;21(5):432–40.
**Additional Information:**	
**Question**	**Response**
Are you submitting this manuscript to a special series or article collection?	No
**Experimental design and statistics**<br><br>Full details of the experimental design and statistical methods used should be given in the Methods section, as detailed in our Minimum Standards Reporting Checklist. Information essential to interpreting the data presented should be made available in the figure legends.<br><br>Have you included all the information requested in your manuscript?	Yes
**Resources**<br><br>A description of all resources used, including antibodies, cell lines, animals and software tools, with enough information to allow them to be uniquely identified, should be included in the Methods section. Authors are strongly encouraged to cite Research Resource Identifiers (RRIDs) for antibodies, model organisms and tools, where possible.<br><br>Have you included the information requested as detailed in our Minimum Standards Reporting Checklist?	Yes
**Availability of data and materials**<br><br>All datasets and code on which the conclusions of the paper rely must be	Yes

either included in your submission or deposited in publicly available repositories (where available and ethically appropriate), referencing such data using a unique identifier in the references and in the “Availability of Data and Materials” section of your manuscript.<br><br>Have you have met the above requirement as detailed in our Minimum Standards Reporting Checklist?	

Powered by Editorial Manager® and ProduXion Manager® from Aries Systems Corporation

Reviewer reports:

Reviewer #1: In this study Høye describe an analysis of the T cell receptor (TCR) repertoires of tumours from patients with colorectal liver metastases (CLM), who received different neoadjuvant chemotherapy (NACT) regimes. Their results agreed with their prior results from another cohort with orthogonal techniques: it appears that a short-term treatment (<9.5 weeks) associates with a increased T cell infiltration relative to longer intervals, and that repertoires of NACT-treated tumours in general showed greater clonality, indicative of clonal expansion.

It is an extremely well written and plotted manuscript, employing sensible and robust analyses on a reasonably-sized cohort of patients. Use of the T cell fraction in particular is very appealing potential biomarker that could be used in the adoption of repertoire sequencing for a greater range of clinical conditions, while still supplying the additional information repertoires bring. The data and code were also made available to the reviewers, and there's every indication that these will be suitably available upon publication. I am very happy recommending its publication, as I think it will be of use to the field. I have noted a small number of areas that caught my attention for the authors' consideration: several are places where some additional plotting may help reduce the possibility that the results are confounded, while the rest are minor typographical comments or similar.

We extend our heartfelt gratitude to Reviewer 1 for the excellent and very thorough review, and for the constructive comments that improved the manuscript. See below our replies to this very detailed and excellent review.

1) I have a couple of queries about the T cell fraction. While none are major, given the importance of the metric to the study these are probably worth addressing:

> **a. L143 states that the number of unique CDR3b sequences is used in its calculation. Could the authors please clarify in the text to specify whether different recombinations (potentially using different V/J genes) producing the same CDR3s are grouped together in these calculations?**
>
> In the event of different V/J gene recombination resulting in the same rearrangement sequence, these were not grouped together, and counted separately. T cell fraction was calculated by the sum of rearranged DNA templates detected in each sample (a proxy for the number of T cells detected), divided by the total number of genomes (calculated from the amount of input gDNA in the first library preparation mix). Counting the same sequential rearrangement produced from different V/J gene rearrangements separately or together will therefore not make a difference on the T cell fraction calculation. The lines 144-146 have been adjusted to make this clearer.

b. Given that the T cell fraction is calculated using the number of TCR rearrangements per sample, there's a chance that it might be confounded by differences in sequencing depth or errors, which are both in part dependent on the amount of input DNA. To rule these possibilities it might help the reader to include plots showing a lack of correlation between T cell fraction and number of productive TCR rearrangements, and between T cell fraction and amount of input gDNA. Additionally randomly selecting a matched number of sequences per donor prior to metric calculation can help rule out the possibility that these variables might be confounding (similar to how higher q values remove the effects of small clones in the later diversity metrics).

Regarding T cell fraction and sequencing depth, all samples were sequenced with the same number of PCR replicates, which is what determines sequencing depth. Sequencing coverage in the Adaptive Biotechnologies ImmunoSEQ platform is calculated as the average number of reads per rearranged DNA template (proxy for the number of T cells) prior to PCR amplification. This number is possible because of the presence of synthetic rearrangements diluted to be present at either 0 or one template in library preparation mix prior to PCR amplification. Sequencing coverage is therefore expected to decrease as the number of T cells present in the tissue increases.

The below plots show the relationship between coverage, input gDNA in the library preparation mix and T cell fraction.

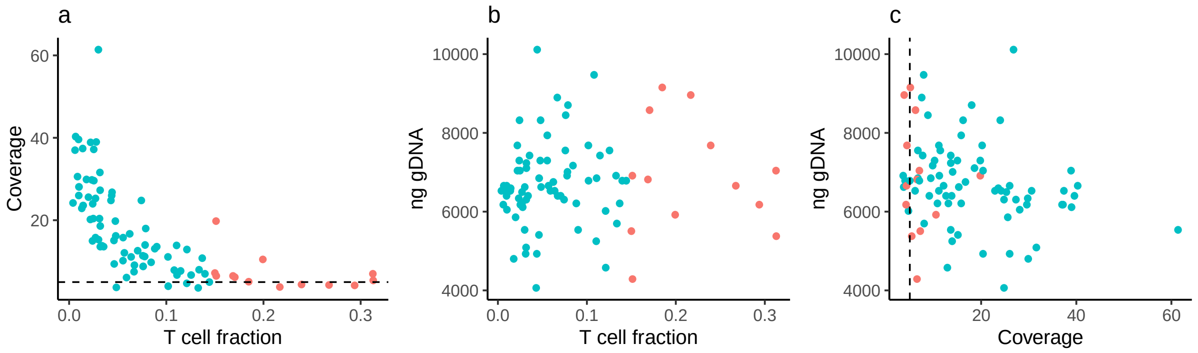

Points were colored based on whether the T cell fraction was greater than 0.15. Dashed lines represent sequencing coverage of 5.

a. Samples with very high T cell fraction leads to low sequencing coverage.
b. There was no correlation between T cell fraction and input gDNA.
c. Nor was there a strong correlation between gDNA and coverage.

All biological samples were run using two PCR replicates. The ImmunoSEQ protocol is optimized for 30 000 to 45 000 rearranged T cell genomes per PCR replicate. The median number of productive rearranged DNA templates (proxy for number of T cells) per PCR replicate in this dataset was 31 532, well within the optimal range. However, among the samples with high T cell fraction, the number was much higher, the maximum being 166 672 rearranged templates. The low coverage for these samples

seen in plot **a** was likely caused by oversaturation of T cells. Ideally, the input DNA in these samples would have been reduced prior to library preparation, so as to have the optimal T cell number per PCR replicate. However, the T cell content for each tissue was not known prior to sequencing. As can be seen from plot **b**, T cell fraction was not impacted by variation in input gDNA, nor was coverage (plot **c**).

For T cell fraction analysis, **the lower coverage and oversaturation of T cells for CLM tissue with very high T cell content is not of major concern**. Potential bias likely reflects an underestimation. However, for clonality analysis low coverage is a major concern, resulting in a bias towards abundant clones. Therefore, samples with low coverage (< 5) were dropped from the downstream clonality analysis in order to ensure unbiased analysis.

The below plot show that there was no association between clonality and coverage.

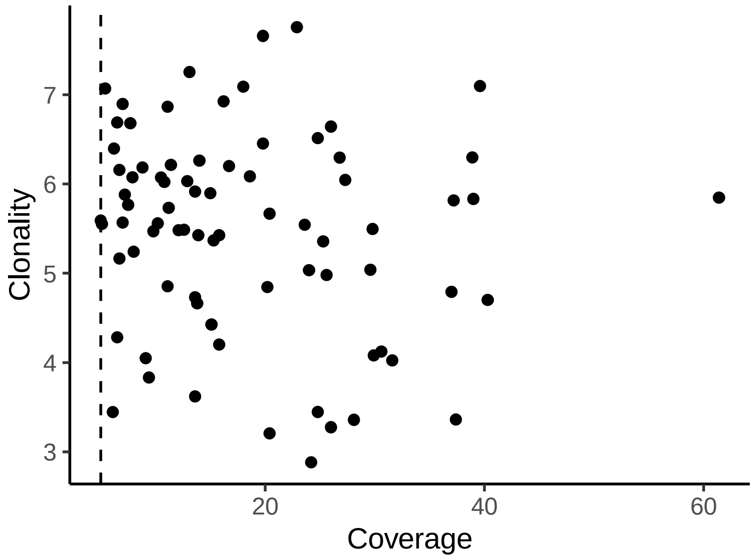

Linear regression (clonality ~ coverage) intercept = **5.74**; slope = **-0.015** (95% CI: -0.039 – 0.009); p-value = **0.2**. Clonality was evenly distributed over the coverage distribution, and the slight negative trend in clonality for increasing coverage was likely random noise. This shows that Hill evenness based clonality estimates are robust both to variation in absolute T cells, and also robust to variations in sequencing coverage.

These figures have been added to the supplementary file. Lines 135-136 of the manuscript have been updated to make the rationale for input gDNA per PCR replicate clearer.

c. Re: Fig 1: while the range of interval length is given, the distribution is not. I wonder if perhaps a (potentially supplementary) plot of duration of interval vs T cell fraction would be instructive. This would also be a potentially independent useful validation of the use of 9.5 mo as a threshold, which it seems was determined by the authors in a significance screen by ROC analysis in their prior publication. As the discussion remarks on the agreement between the results of this study broken by

interval length and that previous publication, the authors may also wish to consider repeating that ROC analysis on these data, to see if a similar threshold is produced.

We agree that it would be valuable to have an independent validation of the 9.5 months cutoff, however the material produced in this study is not suitable for this task. Samples were selected with the purpose of acquiring sufficient samples in each NACT-interval group for meaningful statistical analysis. The time interval distribution is not independently sampled, and would not be suitable for statistical analysis. The minimum and maximum interval in weeks for the two groups was already described on L117, but we have also added the median.

2) The Hill diversity/evenness data beautifully plotted in Fig 2 a/b is a robust way to illustrate these 'species'-level metrics.

a. However in my experience it can't be expected that the reader will know how to interpret these plots: even many people who study repertoire data are unfamiliar with these more ecological measures. I imagine this is perhaps even more true of the oncologists who may be interested in this study. I wonder if perhaps an explanatory sentence or two in the legend might help? I know there is some of this in the discussion, but that's likely going to be seen after the figures for many readers.

Additional explanatory text has been added to the relevant legends. Updated figure 2 a and b legend text.

b. Also I think maybe the x axes would more accurately be labelled 'q values' (rather than 'α').

This is correct, we apologize for this mix-up. We have altered the x axis label to q. Figure 2 a and b have been updated.

d. Some other values can be guessed at but aren't specifically defined in the legend, e.g. it only defines the median for Fig 2c and not the whisker/boxes, while the shaded confidence interval area in 2d is similarly not defined. It's also unclear where the T cell count information is coming from in this plot: were orthogonal T cell count data collected, or is this inferred from the repertoires?

The relevant figure legends have been updated to include these points. Figure 2 d legend text has been updated.

3) The network analyses of Fig 3/4 are interesting, but currently presented in a way was harder to intrepret than surrounding sections, with several fairly field-specific analyses done with little explanation.

a. The text results of this analysis (beginning on L257. "The mean number of nodes detected was...") is abrupt, discussing results of an analysis that hasn't been established yet. This continued through the paragraph. E.g. it's unclear why the test for power law was performed: while it is well known that clonal repertoires tend to

follow such distributions, the authors don't state why they think the network connectivities should.

This is a fair point, and we agree that this section may be somewhat abrupt. Our rational for this analysis was to see whether there may possibly be a convergence of TCR sequences based on shared specificity of antigen epitopes. However, in this case there was no evidence for such a convergence. We have updated the text (L259 – L261). We have also added a sentence on the background for why we conducted a test for whether the degree distribution followed a power law distribution. We have updated the text (L266 - L268) to make the purpose and our prior expectations clearer.

b. Following on from my point (1) above, a common confounder in such connectivity networks is the possibility for sequencing and PCR errors to artificially inflate networks. The incidence of such errors could be biased towards certain samples, depending on factors like gDNA input and number of PCR cycles. You would also expect more abundant clonotypes on average to produce greater numbers of erroneous sequences, as a function of their occupancy of the reactions involved, which could contribute to the correlation seen in Fig 3a. Some control analyses or discussion might be useful, to help discount this possibility (with other plots in this figure lending weight to the notion that this is not artefactual).

This is a fair point, and we have added another sentence explaining why these sequence convergences occur (L388-390). However, this does not alter the primary conclusion of this manuscript. The sequence convergence in the network analysis were mainly composed of singleton or low abundance clones. Expanded clones were, in contrast, sequentially distinct and disconnected from the other clones in the network analysis, as can be seen in figure 4. The finding from this study therefore suggest that the T cell infiltration is driven by a heterogenous neoantigen landscape, unique to each patient/tumor.

c. Again there are some missing value definitions: 3a shaded area, box/whisker markers in e-g. The 3f y axis label is also mislabelled: 'MOI' should likely be 'MHI'.

Figure 3 legend have been updated to include these definitions, and Figure 3f axis label has been changed to MHI. We thank the reviewer for making us aware of these omissions.

4) It is also probably worth noting somewhere in the text that the connectivity difference between public/private sequences is exactly what one would expect given our understanding of biased V(D)J recombination (which underlies the production of public sequences in the first place). https://doi.org/10.1073/pnas.0608907103 would be a classic citation for this, with more recently validations using higher-throughput TCRseq data discussed in https://doi.org/10.1111/imr.12665.

a. It has also similarly been observed that many T cells detectable in tumours are specific for viruses in both humans and mice, e.g. https://doi.org/10.1038/s41586-021-03704-y, https://doi.org/10.1172/JCI150535, and https://doi.org/10.4049%2Fjimmunol.1601064.

As mentioned above, we have added another sentence (L388-390) that makes this point, and included the mentioned references. Furthermore, the results from the analysis of TCR sequences present in the McPAS database revealed that the most common pathological associations included influenza, tuberculosis, cytomegalovirus and epstein barr virus. This has already been mentioned in the text. In regards to the conclusion, we think that it is significant that, as can be seen in fig3g, the most expanded clones also had fewer hits in the McPAS database. This suggests these expanded clones are likely specific to private neoantigens.

5) As noted above, both the data and code are available to the reviewers, which is great. However there are a few things that could be changed to align more with the reproducibility and data reuse this journal prides itself on.

a. The Github repo seems to have had a working directory uploaded to it, along with the associated detritus that such folders generate. There are several scripts and tables that seem to be derived from each other (eg X, X_v2), and at least one directory of what I suspect is analyses that didn't make it to the manuscript ('gliph2'). Tidying up these redundancies and not needed files would make the repo more navigable.

We have tidied up the repository to make it easier to navigate for other researchers. Metadata files have been merged and cleaned up, and a more fleshed out README.md file should make the repository more navigable.

b. Similarly, while many of the scripts are sensible named, commenting is sparse and there's no summary or record of what metadata is recorded where and what scripts do what. While I'm sure the researchers running the analyses know exactly how to navigate this repo, anyone wishing to repeat or reapply these analyses will not, so an expanded README or similar would help a lot. This would be especially useful for the core pipeline of the study, and the key metadata values (e.g. Adaptive sample ID, patient IDs for those with >1 sample, treatment status/interval, ng DNA etc).

The README.md file in the repository has been fleshed out to make it clearer. All metadata has been cleaned up so that there are now only three files:

- SampleOverview.tsv (Sequencing metadata and input gDNA)
- metadata.xlsx (clinical metadata and also show which samples were from patients sequenced more than once)
- qcReport.tsv (mainly for sequencing coverage)

c. It looks like the journal's policies call for DOIs for code as well as data: I don't know about their provided Code Ocean option, but generating a Zenodo DOI for a GitHub release is very straightforward, and makes sense seeing as the code is already there.

This is a great suggestion; we have generated a Zenodo DOI to the github repository: DOI: 10.5281/zenodo.7614598

6) A few of the axis tick label fonts are small relative to the rest of the figure (e.g. Fig 3a/c), so making these larger would help the short sighted readers like myself from having to frequently zoom in.

The font sizes in Fig 3a/c have been increased.

7) L202-204, sample exclusion. It's not entirely clear to me what "sequencing coverage <5" means here: fewer than five different TCRs, or counts, or something else? In any case it should be stated which of the three test arms (no-NACT/short/long) they fell from.

Sequencing coverage was calculated as the mean number of reads per rearranged DNA template in the original library prep mix prior to PCR amplification (which was used in this study as the proxy for number of T cells, as described above). This number can be calculated from post PCR sequencing data because each library preparation mix also contain synthetic rearranged DNA templates diluted to be present at either 0 or 1 template. This allows one to count backwards the number of DNA templates present in the original mix. A general recommendation is that, at a minimum, the number of sequencing reads should exceed the clonal diversity (1). Clonal frequency distribution for all sequencing datasets in this study were at least somewhat skewed, therefore the clone to cell ration was below 1 for all samples. The <5 coverage cut-off was the recommendation from consulting with Adaptive Biotechnologies. Because it is calculated based on reads per cell, not based on reads per clone, it is well above the minimum sequencing depth recommendation. L208 has been updated to include n for each group.

8) L183: the version/accession date of when McPAS was used should be stated. It might even be worth depositing the version of the database that was used somewhere accessible (e.g. the analysis repo), as since Nir Friedman's sad passing it appears that the resource's original URL is no longer working

This is a good suggestion, we have added the file used to query to the github repo, and have added the download date on lines L185-L186.

9) Use of the word 'monoclonal' in L287-288 ("...a very large monoclonal network (Clonality=6.9) is visualized...") seems counter-intuitive, seeing as the sentence later points out how there are tens of thousands of different CDR3s are involved.

We have re-phrased some of the sentences in this section to make it clearer. L292, L295 and L298 have been edited.

10) L254, possible typo or unclear abbreviation: "median og y"

Thank you for making us aware, we apologize for this mistake and have corrected the typo.

11) Several times 'sequential' is used to mean 'relating to sequences'. I think most people will be able to figure out what this means, but (at least in my specific dialect of English)

'sequential' is mostly only used to describe things which occur in a sequence, rather than describe the sequences in the abstract themselves, so this threw me each time it came up. Changing to 'sequence' (e.g. L41/42, "TCR sequential convergence" could become "TCR sequence convergence") would avoid this if the authors wished.

L41 has been changed to TCR sequence convergence.

- Jamie Heather

Reviewer #2: This manuscript by Høye et al analyzed the effects of NACT on the TCR repertoire of TIL in CLM. Using several unique approaches of data analysis, they concluded that NACT increased the T cell infiltration and clonal expansion. Overall, data and conclusion seems to be convincing. The results that "shared clones have higher connectivity of TCR sequences" and "shared clones are enriched with antigen-specific TCRs registered in the database" were novel and interesting. On the other hand, as the authors point out in the discussion, there have been similar studies on other types of cancer, and the "increase in clonality with neoadjuvant chemotherapy" is not novel. It was difficult for me to understand the contents of the Results because the indicators used to evaluate the repertoire were not popular. I think it would be better to move some of the information on the significance of each indicator and the purpose of the analysis from the Discussion to the Results, and make the Results easier to read.

We thank reviewer 2 for taking the time and effort to review our manuscript. Both reviewer 1 and 2 have suggested to add more explanatory text to the results section. We have added sentences to lines L259-L261 and L266-L269 in order to make the purpose of the network analysis clearer, as well as expanding the fig 2a and 2b figure legends so that it is easier to interpret for readers who are not familiar with Hill diversity and evenness profiles.

Major points

1. Line 247-248: clonality is evaluated using AUC of Hill diversity profile. However, since the "1-Pielou" index is commonly used in studies on TCR repertoire analysis, it would be better to show the calculation results of 1-Pielou index as a Fig.

> While many studies on TCR repertoire analysis use 1-Pielou index, the Hill diversity profiles, in a sense, already contain the Pielou index. This is because the Hill function at q=1 is mathematically equivalent to the reciprocal of Shannon entropy, as was proven by Hill in 1973. Pielou index is derived from Shannon entropy, thus is included in the diversity profile in fig 2a. The reason for using the whole profile is because different diversity indexes can yield qualitatively different answers on which repertoire is the most diverse, thus one should assess them based on a range of diversity estimates, such as that shown in fig2a. The AUC based clonality estimate was primarily intended for making a convenient single point estimate for statistical comparison, but one should consider the entire profile when evaluating clonality, i.e. all of fig2a, b, c.

2. line 238-241: Hill diversity profile was not explained, and it was difficult to understand. It would be helpful to include the information equivalent to Discussion line302-309.

> This was also a request from reviewer 1, and we have added some additional explanatory sentences in the figure legends. Figure legend 2a and 2b have been extended to make it easier to interpret for readers who are not familiar with Hill diversity and evenness profiles.

3. Fig. 2 claims an "increase in Clonality with neoadjuvant chemotherapy". Although this study did not separate CD4+ T cells from CD8+ T cells in the TCR repertoire analysis, it has been reported that CD4+ T cells have higher Clonality than CD8+ T cells in their repertoires

(Gueguen et al. 2021, Zhang et al. 2018). Therefore, the increased Clonality of tumor T-cell repertoires reported in this study may only reflect an increased proportion of CD8+ T cells in tumor-infiltrating T cells (Rudqvist et al. 2018). This point should be mentioned as a limitation in Discussion.

This is a fair point, and we have added a sentence to the limitations part of the discussion, L394-L395. We agree that this is a limitation, however Dagenborg et al, 2020 (2) found that, using immunohistochemistry on a subset of the same cohort, both CD4+ and CD8+ T cells had higher infiltration in the short-interval group compared to the no-NACT and long-interval group.

4. Line 257-259: I couldn't figure out why the authors performed network analysis just by reading the result. Please include something like Discussion line 359-363 in the result.

This was also requested by Reviewer 1, we have expanded this section to explain the purpose of these analysis, L259-L261.

5. Line 278-279: Please provide a table with a list of target pathogens (flu, tuberculosis, etc.) of the clones that matched the McPAS database. Also, please indicate what type of cancer antigens (differentiation antigen, cancer testis antigen, neoantigen, etc.) are listed as "colorectal cancer".

The table of unique CDR3 amino acid hits to the McPAS database, for both the public and private most prevalent clones, have been added to the github repository:

- data/McPAS/public_cdr3_aa_unique_McPAS_hits.csv
- data/McPAS/private_cdr3_aa_unique_McPAS_hits.csv

Regarding McPAS, it is a database of TCR sequences that have been reported in the literature to be associated with pathological conditions. The specific antigens they respond to is not necessarily known. Our understanding of TCR to antigen specificities is limited, due to the vast diversity of both TCR sequences, and potential tumor neoantigens. Exploring TCR:antigen specificities is outside the scope of this work, and not currently possible with sequencing technology alone.

6. Line 281-290: Fig. 4 only shows an example of TCR network, so I felt that the order of presentation should be switched with Fig. 3.

While we can understand this point of view, the information in Fig. 3 is essential for understanding Fig. 4. We therefore think the order is correct.

7. Line 340-343: "differences in clonality between NACT exposed and unexposed CLM might be of clinical relevance." I was not sure what this "clinical relevance" meant. Also, if the authors are trying to claim similarity of their tumor microenvironment based solely on the similarity between "differences in TCR repertoire index between immune cold SCLC and immune hot NSCLC" and "differences in TCR repertoire index of CLM with and without NACT", it is an overstatement.

We believe that the difference in clonality may reflect differences in response to chemotherapy. This may be important in the context of immunotherapy in mCRC. This hypothesis, where priming the immune system with chemotherapy in a brief time interval prior to immunotherapy, is currently being tested in the randomized control trial METIMMOX (NCT03388190). Regarding the analysis of clonal overlap between repertoires, our findings (Fig. 3f) showed that there was no clonal overlap between patients, while repertoires from the same patient but sampled at different locations of metastasis in the liver showed only modest overlap. Repertoires generated from the same metastatic aliquots had almost complete clonal overlap. Our conclusion from this is again that this is suggestive of a CLM neoantigen landscape that is highly diverse and private to the individual patient. NSCLC in contrast had far higher intratumoral TCR homogeneity, suggesting the neoantigen landscape of these tumors is shared for the majority of tumor cells. This is an important predictor for whether current immunotherapy approaches will have an effect, and may be part of the reason for the very good response in NSCLC compared to the poor response in SCLC and also in mCRC. We agree however that one must be careful comparing these studies, due to possible differences in sampling strategies, etc.

8. Line 352-355 compares the heterogeneity of TCR repertoires in tumors between NSCLC and SCLC, which seemed not relevant to the content of our paper.

We think this is interesting in the context of potential response to immunotherapy. The more homogenous TCR repertoire in NSCLC is suggestive of a homogenous neoantigen landscape, which is an important predictor of response to immunotherapy. See also reply above.

Minor points

1. line 242: I do not understand what "increasing value of q" refers to. Is it referring to the "alpha" in Fig2 a, b?

We apologize for this mistake, which was also pointed out by reviewer 1. The Fig. 2a and 2b x axis label have been fixed to *q* instead of alpha.

2. In Fig. 2b, the plot for the treated group was lower than the plot for the untreated group, but I did not immediately understand the correspondence between this and the statement "Clonality was higher in the treated group". Legend stated that "Clonality was evaluated by 10-AUC", which I think should be clearly stated in the RESULT.

The evenness profile curves should be read as follows. If the curve follows a straight line (evenness close to 1.0) for the entire range of q values, the clonal frequency distribution is completely even (all clones are identical at the same frequency, a completely heterogenous distribution). The further and more rapidly the curve drops as the value of q increases, the more uneven the clonal frequency distribution. (i.e. the more it is dominated by a small subset of very frequent clones). Legends for Fig2a and 2b have been updated, as was also requested by reviewer 1. The 10-AUC is explained in the methods section, L157-L161.

3. line 250-251: "regression analysis revealed a modest association between a high absolute number of T cells and clonality," I could not understand what this analysis was intended to claim.

We included this because it suggests that the higher number of infiltrating T cells is driven by clonal expansion.

4. Lines 259-260: I didn't understand what you were trying to argue with this analysis.

We have added some explanatory text that explains the purpose of the network analysis. See L259 – 261, this was also requested by reviewer 1.

5. Line 262-264: The authors compared the clonality between repertoire that satisfy the power-law fit test and those that do not. Please provide a dot plot in addition to the average value.

While we can understand why an additional plot might be useful, the take home message from the power law was that the networks produced in this study did not follow a power law. Prior studies on antibodies have found that the degree distribution of neoantigen experienced networks follow power law, and our rationale for conducting this analysis was that this might have been an exiting tool for assessing whether the repertoire is antigen experienced or not. However, it appears that T cell repertoires follow different dynamics, and the results of this analysis was negative.

6. Line 264-265: It is an overstatement to conclude that "clonally expanded datasets were not associated with power law degree distribution" because there are only a few repertoires that satisfy the power-law fit test.

As also explained above, the overall conclusion from this analysis was that TCR repertoire networks do not follow the same dynamics of antibody repertoires, and power law test are, regrettably, not a suitable tool for assessing if a repertoire is antigen experienced, in this context.

7. Line 268-270: Fig3d might be a mistake for Fig3e. Also, Fig3e does not seem to be a graph comparing "mean connectivity fractions".

Again, we apologies for the error in figure reference, which have been corrected. Regarding Fig3e, the repertoires were randomized to 1000 clones each, therefore the numbers are equivalent to fractions which is written in the text.

8. line 270: Fig3e" seems to be a mistake for "Fig3f".

Again, we apologies for the error in figure reference, which have been corrected.

9. Line 270-271: "in line with", but I did not understand how the discussion of public clone connectivity corresponds to the discussion of repertoire similarity between samples.

We have rewritten this line to instead say “further illuminated by”, to make our intension clearer, L280.

10. Spelling error: line 68 "Characterisation"

We have changed this word to characterization.

11. Fig3f, Y-axis seems to be MHI.

Corrected, as was also mentioned by reviewer 1.

References

1. Greiff V, Miho E, Menzel U, Reddy ST. Bioinformatic and Statistical Analysis of Adaptive Immune Repertoires. Trends Immunol. 2015;36(11):738–49.

2. Dagenborg VJ, Marshall SE, Yaqub S, Grzyb K, Boye K, Lund-Iversen M, et al. Neoadjuvant chemotherapy is associated with a transient increase of intratumoral T-cell density in microsatellite stable colorectal liver metastases. Cancer Biol Ther. 2020 May 3;21(5):432–40.

Manuscript

Click here to access/download;Manuscript;CLM-TCRseq_gigascience_reviewer_revised_v2.docx

T cell receptor repertoire sequencing reveals chemotherapy-driven clonal expansion in colorectal liver metastases

Eirik Høye[1,2], Vegar J. Dagenborg[1,3], Annette Torgunrud[1], Christin Lund-Andersen[1,2], Åsmund A. Fretland[4,5], Susanne Lorenz[6], Bjørn Edwin[2,4,5,], Eivind Hovig[7], Bastian Fromm[8], Else M. Inderberg[9], Victor Greiff[10], Anne H. Ree[2,11], Kjersti Flatmark[1,2,3,*]

[1] Department of Tumor Biology, Institute for Cancer Research, The Norwegian Radium Hospital, Oslo University Hospital 0379 Oslo, Norway

[2] Institute of Clinical Medicine, Medical Faculty, University of Oslo, 0318 Oslo, Norway

[3] Department of Gastroenterological Surgery, The Norwegian Radium Hospital 0379 Oslo, Norway

[4] The Intervention Centre, Rikshospitalet, Oslo University Hospital, 0372 Oslo, Norway

[5] Department of Hepato-Pancreato-Biliary Surgery, Rikshospitalet, Oslo University Hospital, 0372 Oslo, Norway

[6] Department of Core Facilities, Institute for Cancer Research, The Norwegian Radium Hospital, Oslo University Hospital, 0379 Oslo, Norway

[7] Center for Bioinformatics, Department of Informatics, University of Oslo, 0316 Oslo, Norway

[8] The Arctic University Museum of Norway, UiT – The Arctic University of Norway, 9037 Tromsø, Norway

[9] Translational Research Unit, Department of Cellular Therapy, Oslo University Hospital, 0379 Oslo, Norway

[10] Department of Immunology, University of Oslo and Oslo University Hospital, 0372 Oslo, Norway

[11] Department of Oncology, Akershus University Hospital, 1478 Lørenskog, Norway

* Corresponding author at: Department of Tumor Biology, Institute for Cancer Research, The Norwegian Radium Hospital, 0379 Oslo

E-mail address: kjersti.flatmark@rr-research.no

Abstract

Background: Colorectal liver metastasis (CLM) is a leading cause of colorectal cancer mortality, and the response to immune checkpoint inhibition (ICI) in microsatellite stable CRC has been disappointing. Administration of cytotoxic chemotherapy may cause increased density of tumour infiltrating T cells, which has been associated with improved response to ICI. This study aimed to quantify and characterize T cell infiltration in CLM using T cell receptor (TCR) repertoire sequencing. Eighty-five resected CLM from patients included in the Oslo CoMet study were subjected to TCR repertoire sequencing. Thirty-five and 15 patients had received neoadjuvant chemotherapy (NACT) within a short or long interval, respectively, prior to resection, while 35 patients had not been exposed to NACT. T cell fractions were calculated, repertoire clonality was analysed based on Hill evenness curves, and TCR sequence convergence was assessed using network analysis.

Results: Increased T cell fractions (10.6% vs 6.3%) were detected in CLM exposed to NACT within a short interval prior to resection, while modestly increased clonality was observed in NACT exposed tumours independently of the timing of NACT administration and surgery. While private clones made up >90% of detected clones, network connectivity analysis revealed that public clones contributed the majority of TCR sequence convergence.

Conclusions: TCR repertoire sequencing can be used to quantify T cell infiltration and clonality in clinical samples. This study provides evidence to support chemotherapy-driven T cell clonal expansion in CLM in a clinical context.

Key words

- Colorectal cancer
- T cell receptor sequencing
- Liver metastasis
- Neoadjuvant chemotherapy

- Clonal expansion
- Clinical samples

Introduction

Increased understanding of how the immune system is involved in cancer development and progression has resulted in development of therapeutic interventions successfully targeting the immune system, such as the immune checkpoint inhibitors (ICI) targeting the PD-1/PD-L1 axis. Mismatch repair deficient cancers with high tumour mutational burden have been shown to respond strongly to ICI regardless of histologic type [1], which has led to interest in identification of specific tumour neoantigens. Characterization of tumour antigen-specific T cells has therefore become an important step to further understand anti-tumour immunity. The non-coding part of the genome is increasingly perceived as a major contributor to tumour neoantigens, as up to 70 % of the genome is transcribed in some form [2]. Abnormally expressed RNA in tumour tissue could therefore contribute as neoantigens in cancers with modest tumour mutational burden. T cells recognise their cognate antigens through interaction with the peptide-MHC complexes presented on the surface of target cells via the complementarity determining region 3 (CDR3) of the T cell receptor (TCR). Recent advances in deep sequencing of the TCR CDR3 region have enabled quantification of T cell clones with the same antigen specificity and characterisation of TCR repertoires across biological compartments and over time [3,4]. Applying this technology to metastatic tumor samples represents an important opportunity to further understand T cell immunity in metastatic cancer and possibly identify neoantigens associated with immune responses.

Colorectal cancer (CRC) accounts for about 10 % of all diagnosed cancers and cancer-related deaths worldwide, and colorectal liver metastasis (CLM) is a leading cause of CRC-related mortality [5]. Surgery is a curative treatment option for a minority of patients with limited metastatic disease, but for most patients with CLM, systemic chemotherapy is the main treatment option, and the survival rates are poor [6]. Durable responses to ICI have been observed in CRC patients with microsatellite instable tumours, but with the majority of patients suffering from microsatellite stable (MSS) cancers, responses are generally disappointing [7]. Still, in MSS CRC there is evidence to suggest that the microenvironmental immune

contexture is important, such as in primary CRC, where a high density of tumour infiltrating T cells was shown to strongly correlate with a favourable long-term outcome [8]. In previous studies of CLM we observed up-regulation of immune-related genes and increased T cell intratumoral densities after exposure to neoadjuvant chemotherapy (NACT) [9,10]. This suggests that NACT can modify the CLM immune microenvironment, possibly by induction of immunogenic cell death, towards a state that could potentially be more responsive to ICI.

In this work, we have sequenced TCR repertoires in resected CLM from 85 patients included in the OSLO-COMET study and repertoires were compared according to NACT exposure to analyse T cell fractions and clonality. In addition, network analysis was used to assess sequential convergence reported to be associated with antigen-experienced repertoires.

Methods

Patient samples

CLM samples were collected from 85 patients included in the *Oslo Randomized Laparoscopic Versus Open Liver Resection for Colorectal Metastases Study* (OSLO-COMET study; NCT01516710) (for clinical data, see Table 1). Written informed consent was obtained from all participating patients. The OSLO-COMET study was approved by Norway's Regional Committees for Medical and Health Research Ethics (ID# 2011/1285/REK sør-øst B). Neoadjuvant chemotherapy (NACT) was administered to 50 patients (59%), while 35 patients (41%) did not receive NACT (no-NACT group) (Fig. 1a). The NACT regimen, the timing of liver resection after NACT, and the number of NACT cycles were not predefined by the OSLO-COMET study protocol, but were decided for the individual patient by a multidisciplinary team [9]. We previously determined that a 9.5-week interval between completion of NACT and liver resection was a cut-off for observing an increase of intra-tumoral T cell density [9]. Applying the cut-off to this cohort, 35 (41%) and 15 (18%) patients had received NACT less, and more than 9.5 weeks prior to liver resection, respectively (hereafter termed the short-interval (median 7, min-max 2.9-9.5 weeks) and long-interval (median 16, min-max 9.5-25.9 weeks) groups. Tumour samples were fresh frozen in liquid nitrogen and stored at -80˚C. The tumour content was evaluated by the study pathologist and the tissues were processed and homogenized as previously described [9]. Briefly, DNA was isolated using the Allprep DNA/RNA/miRNA Universal Kit (Qiagen, Düsseldorf, Germany; Cat. No. 80224) and DNA purity was determined using the Nanodrop 2000 spectrophotometer (Thermo Fisher, Waltham, Massachusetts, USA). Aliquots were diluted to 200 ng/µL, as measured with the QuBit dsDNA Broad Range Assay Kit (Thermo Fisher; Cat. No. Q32850). Three of the 85 patients included in the study had two TCR repertoire datasets from the same metastasis but different tissue aliquot, representing technical replicates. For four patients, TCR repertoire datasets were derived from two separate metastases resected at the same procedure, providing information about potential heterogeneity of TCR repertoires in metastases located in the same liver.

T cell receptor sequencing

TCR repertoire sequencing libraries were prepared using the hsTCRB v3 immunoSEQ library preparation kit (Adaptive Biotechnologies, Seattle, Washington, USA). Briefly, two 16-µL polymerase chain reaction (PCR) replicates were prepared for each biological sample, with gDNA content within the range recommended by the manufacturer for non-lymphoid tissues, which is predicted to yield the optimal 30 000 – 45 000 T cells per PCR replicate. The library preparation protocol uses multiplex PCR with primers for all possible V and J fragments in the TCR-beta gene with adjustment of primer concentrations to account for variable primer efficiencies. Synthetic repertoires with known quantities are present in each PCR reaction to allow accurate quantification of T cells in each sample [11]. Pooled libraries were sequenced using the NextSeq 500/550 Mid Output kit v2.5 (150 cycles) (Illumina, San Diego, California, USA). Bioinformatics processing of raw data was done by Adaptive Biotechnologies proprietary analysis pipeline which provided data on the immunoSEQ rearrangement-level file format. The T cell fraction was determined by dividing the ~~number~~ sum of detected rearranged ~~CDR3β~~ DNA ~~sequences~~ templates (~~as a proxy for the number of T cells~~one rearranged DNA template as a proxy for one T cell) with the total number of genomes in the sample which was calculated by total amount of DNA/6.6 pg (mean DNA content/cell). T cells with identical CDR3β sequence and length were defined as one clonotype for subsequent analyses. As previously described, a clone was considered "private" if detected in only one patient, with "public" clones being present in more than one patient [12], and the sharing level was defined as the number of patients in whom a specific clonotype was detected.

TCR repertoire clonality using Hill diversity and evenness profiles

The alakazam v1.1.0 R package [13] was used to generate Hill diversity and evenness profiles for each dataset, as previously described [14]. The range of q parameters was set between q=0 to q=10, with steps of 0.2. Evenness profiles are defined by the equation $^{\alpha}E = {}^{\alpha}D / SR$ (or the Hill diversity profile divided by Hill diversity at q=0), as previously described in [15]. For

each repertoire, a clonality index, defined as ten minus the area under the curve (AUC), was calculated for individual evenness curves using the sintegral() function from the Bolstad2 v1.0-28 R package [16], which gives a parameter ranging from zero to ten, with higher values signifying more oligoclonal repertoires.

Analysis of within ~~TCR~~ repertoire TCR sequence similarity using Levenshtein distance networks

To analyse TCR sequence similarity within T cell repertoires, the ImNet v0.2.1 package was used to generate Levenshtein distance (LD) [17] matrices for all unique CDR3β sequences in each dataset. These distance matrices were then used to construct CDR3β sequential convergence networks, where connections were built between CDR3β sequences at LD=1. Visualization of the networks was accomplished with Cytoscape v3.9.0 [18]. The resulting networks illustrate the overall similarity of CDR3β sequences within a T cell repertoire and the presence of subclusters of CDR3β sequences. Global parameters calculated for each network included the number of nodes (unique clones) and number of edges (clonal connections), and the network connectivity fraction was calculated by dividing edges with nodes. The powerlaw() R package [19] was used to assess whether the network degree distribution followed a power law function. A goodness of fit test was conducted, where the null hypothesis was power law distribution. X-min value was determined for each distribution. A degree distribution with a power law goodness of fit p-value greater than 0.1 was considered a plausible power law distribution. Local parameters to characterize individual clones included the number of degrees (connections between clones in a network) and the sharing level (number of patients where a clone was detected). As was done previously in [12,20], to compare network connectivity of public and private clones, clones from all repertoires were classified as public or private, subsampled to 1000 clones to compensate for the numerical overrepresentation of private clones, and networks were generated with the same method as above.

To further explore potential associations between TCR connectivity, sharing level, clonal expansion and potential disease pathologies, the McPAS database [21] was used. The

database was downloaded on 10.05.2022, and is available in the code repository. Two groups of TCRs were defined, one consisting of top 10% of clones with highest connectivity and/or sharing level, the other consisting of the top 10% most clonally expanded TCRs. The fraction of TCRs that matched at LD = 0 with known pathology associated TCRs in McPAS was calculated for each sample.

Analysis of overlapping clones between repertoires

The Morisita Horn index (MHI) was used to assess clonal similarity between repertoires. This index shows the overall clonal overlap between two repertoires, weighted by clonal frequency, ranging from 0 (no overlap) to 1 (complete overlap). A combined rearrangement file was downloaded from the immunoSEQ Analyzer. This file format has all clones across all datasets for each row, and the count of that clonotype in each dataset for columns. This was input to the divo R package mh() v1.0.1 function, which yielded pairwise MHI comparison for all datasets. Pairwise comparisons were made between repertoires from all analysed patients; in addition, comparisons were made between repertoires generated from different aliquots from the same metastasis (n=3), and repertoires from different metastases in the same patient (n=4).

Statistical analyses

For T cell fraction analysis, one dataset per patient was included (n=85), while for subsequent analyses, in order to avoid bias due to low frequency clones, eight datasets with sequencing coverage <5 were excluded. Total number of samples available for downstream analysis, n=77 (No-NACT = 32, short-NACT = 30, long-NACT = 15). T cell fractions and clonality were described using mean and 95% confidence intervals (CI). Mean T cell fractions and clonality in the NACT administration groups were compared using the Welch t-test using the compare_means() function from ggpubr v0.4.0. Comparison of clonality against the location of the pCRC was also made. Linear regression was used to analyse the relationship between clonality and the number of T cells, and the network connectivity fraction and number of

clones, using the lm() function in base R v4.0.5. Associations between clonality and primary tumour location (right colon, left colon or rectum) were also analysed using the Welch t-test using the compare_means() function form ggpubr v0.4.0. Overall survival was measured from the time of CLM resection. The last liver resection date was on 28 January 2016, and the censoring date was on 8 January 2020. The Kaplan-Meier method was used to estimate patient survival, while the log-rank test was used to see if there was a difference between survival curves of patients with low, medium or high clonality, using the survival v3.2-12 package. P-values <0.05 were considered to indicate statistical significance.

Role of Funding source

This work was financially supported by the South-Eastern Norway Regional Health Authority [grant #2018014, to KF] to fund the PhD position for EH, and Norwegian Cancer Society [grant #215817, to VG].

Results

High T cell fraction was associated with a short interval between NACT administration and surgery

The mean number of productive CDR3β DNA templates per sample was 83 098, with 95% CI [66 615-99 582]. When normalized against the total amount of genomic DNA, the mean T cell fraction was 8.1%, with 95% CI [6.5-9.6%]. The mean T cell fraction was higher in the short-interval group (10.6% [7.4-13.8%]) compared to the no-NACT group (6.3% [4.8-7.9%]), p=0.02 (Fig. 1b). The T cell fraction in the long-interval group (6.2% [3.4-8.9%]) was also significantly lower compared to the short-interval group (p=0.04), while there was no difference compared to the no-NACT group.

NACT exposure was associated with more clonal TCR repertoires

In total, 1 413 435 unique clones were identified across all datasets, median 15 596 (min-max, 1476-66 976). Hill diversity profiles (Fig. 2a) showed considerable variability at the species richness range (q=0) (mean 16 000, 95% CI [13 611-18 388]). Both the short-interval (18 020 [13 524-22 515]) and long-interval groups (16 079 [10 394-21 764]) exhibited non-significant trends towards a higher number of unique clones compared to the no-NACT group (14 069 [10 833-17 304]). At increasing values of q, the mean in the short- and long-interval groups intersected and fell below the mean of the no-NACT group, but with overlapping CIs. When comparing the Hill evenness profiles (Fig. 2b), which are diversity profiles normalized against the number of unique clones (also called species richness), the short- and long-interval groups had very similar means, and the curve for both groups were lower than for the no-NACT group, with non-overlapping 95% CI. Comparison of clonality based on AUC calculations from individual evenness curves showed that the short-interval (5.8 [5.5-6.2]) and long-interval (5.7 [5.2-6.2]) groups had higher mean values than the no-NACT group (5.0 [4.6-5.4]) (p=0.004 and p=0.03, respectively) (Fig. 2c). Furthermore, regression analysis revealed a modest association between a high absolute number of T cells and clonality (Fig. 2d). There were no associations between clonality and overall survival (Supplementary Fig 1), but a non-

significant trend towards increased clonality was observed when comparing samples from right-sided with left-sided primary tumours (p=0.09, Supplementary Fig 2).

T cell similarity networks clustered around publicly conserved sub-sequences

Networks were generated based on the LD of TCR sequences for each repertoire. The purpose of this analysis was to see if there was a tendency of TCR sequence convergence, which might indicate shared specificity to common antigen epitopes. The mean number of nodes detected per repertoire was 16 056, 95% CI [13 637-18 475], while the mean number of edges was 4133 [3015-5251], generating a mean network connectivity fraction of 19.0% [16.5-21.5%]. The connectivity fraction increased linearly as a function of the total number of clones, from less than 5% in small networks, to greater than 50% in large networks (Fig. 3a). The connectivity fraction did not appear to be associated with the clonality of the network. Furthermore, prior studies on antibody repertoires reported that the clonal degree distribution of LD based networks resemble a power law distribution, whereas naïve networks do not [17,22]. While the majority of the networks in the context of T cell repertoires in this study did not follow a power-law distribution (Fig. 3b), four short-interval and five no-NACT repertoires passed the power law fit test. The mean clonality for the networks that passed the test was 4.4, which was lower than the mean clonality across all datasets of 5.5. Clonally expanded datasets were therefore also not associated with power law degree distributions.

Instead, connectivity was associated with public clonal sharing level, where the number of degrees increased linearly with increasing sharing level (Fig. 3c). The majority of the detected clones were private (90.3 %), while 9.7% of the clones were public. (Fig. 3d). The public clones exhibited a much higher level of connectivity than the private clones, with mean connectivity fraction of 20.5% [19.2%-21.8%] compared to 0.4% [0.3%-0.4%], respectively (Fig. 3e). This finding is further illuminated by the MHI comparisons (Fig. 3f), where repertoires from different patients had very low overlap of mean 0.0007 (min-max, 0-0.07). MHI calculated from repertoires generated from different aliquots from the same metastasis were high, with a mean

of 0.93 (min-max, 0.86-0.97), while repertoires from different metastases in the same patient exhibited moderate overlap, with a mean MHI of 0.5 (min-max=0.09-0.97). The TCR clones with highest connectivity and/or sharing level were more commonly associated with known pathogens (10% of clones), according to the McPAS database, compared with the most expanded TCR clones (3% of clones) (Fig. 3g). Interestingly, the five most common pathologies detected were influenza, tuberculosis, colorectal cancer, cytomegalovirus and epstein barr virus.

Three representative networks are shown in Fig. 4. A network from the short-interval group was in the ~~monoclonal~~ highly clonal end of the spectrum (Clonality=7.1) had a connectivity profile with 2907 clonotypes, 188 connections and a network connectivity fraction of 7% (Sample 122; Fig. 4a**)**. The most expanded clone (2053 T cells) was not detected in any other samples, representing a private clone. A more ~~heteroclonal~~ heterogenous, lowly clonal network (Clonality=3.3) from the no-NACT group had a very similar connectivity profile, exhibiting 3005 clones, 165 connections, and a network connectivity fraction of 6% (Sample 40; Fig. 4b). Finally, a very large, highly clonal ~~monoclonal~~ network (Clonality=6.9) is visualized, with 34 849 clones and 13 527 connections, and a high network connectivity fraction of 39% (Sample 37; Fig. 4c). Again, the majority of expanded clones were private to this sample.

Discussion

In this work, using TCR sequencing, we found that the T cell fraction was significantly higher in tumours with a short interval between NACT exposure and liver resection compared to tumours not exposed to NACT. This finding is in line with previously published work from our group, showing higher T cell density in the short-interval group compared to the no-NACT and long-interval groups [9]. Considering that different parts of the tumours were analysed (whole section immunohistochemistry versus snap frozen tissue from the surgical samples) and that different methods were used to quantify T cells, the concordance is remarkable.

Although a significant increase of infiltrating T cells was detected in the short-interval group only, analysis of Hill diversity curves revealed that both the short- and long-interval groups had more uneven clonal frequency distributions compared to the no-NACT group. As previously explained by Hill [14], the diversity at q=0 corresponds to the total number of clones in the repertoire (clonal richness). Because clonal richness is influenced by sampling depth and the presence of rare clones, the frequencies should also be assessed across a range of diversity parameters [14,15], rather than by commonly used single point estimates, to obtain a complete picture of clonal frequency distributions. At q values greater than 2 (Simpson index [14]), the influence of rare clones on the Hill diversity estimate becomes negligible, and is instead influenced by abundant clones. For our datasets, the short-interval group diversity profile was higher at q=0, corresponding with the higher T cell fraction observed in this group. Yet, it intersected and became similar to the no-NACT profile at higher values of q, indicating that the higher clonal richness was driven by rare clones, likely reflecting the higher T cell fraction in this group. However, the slope of both short-interval and long-interval profiles were steeper than the no-NACT profile, still suggesting qualitative differences in the clonal frequency distribution that could be related to NACT exposure. Hill diversity estimates cannot be used to quantitatively compare the clonality of repertoires if the number of sampled T cells is very different. Instead, Hill evenness profiles, which normalize datasets by clonal richness, will provide a more correct comparison of clonality between repertoires. We therefore calculated

AUC values from Hill evenness profiles to compare the degree of clonal expansion between repertoires. The short- and long-interval groups had more monoclonal frequency distributions compared to the no-NACT group. Taken together, TCR sequencing conducted in this study supports our previous finding that NACT exposure leads to a transient increase of intratumoral T cells in CLM, while at the same time resulting in a persistent increase of TCR repertoire clonality. Both findings are in line with the hypothesis that NACT may cause immunogenic cell death, resulting in clonal expansion and T cell response to tumour antigens, including neoantigens.

This work currently represents, to our knowledge, the largest study describing TCR sequencing in metastatic CRC. While studies have been performed in other cancer entities, including breast cancer [23,24], bladder cancer [25], melanoma [26], lung cancer [27–29] and hepatocellular carcinoma [30], most of these have been smaller studies, including 12 to 40 patients. A notable exception was a study comparing TCR sequencing data from small cell lung cancer (SCLC, n=67) [31] with non-small cell lung cancer (NSCLC, n=236) [32], where SCLC tumours were characterized as "cold and heterogeneous" and less monoclonal compared to NSCLC. The estimated T cell fractions in our samples were at an intermediate level (median 5.7%) compared to a very low value in SCLC and the much higher values observed in NSCLC (medians 1.7% and 21%, respectively). When comparing other TCR parameters, our cohort exhibited a higher number of unique clones than either of the lung cancer cohorts (median 15 596, versus 510 and 3246, for SCLC and NSCLC respectively). While differences in absolute values between the studies may be caused by differences in sampling strategy, the detected differences in clonality based on TCR sequencing between the immunologically "cold" SCLC and "hot" NSCLC suggest that the differences in clonality between NACT exposed and unexposed CLM might be of clinical relevance.

Pairwise comparison of TCR datasets from analysis of two tissue aliquots from the same CLM had a high mean MHI (0.9), indicating almost complete overlap of clonal frequencies. In

contrast, the MHI for datasets from different CLM from the same patient was lower (0.3), but still exhibiting a higher degree of overlap than between repertoires from different CLM patients, where the overlap was almost non-existent (0.0007). Although the numbers are low, this finding indicates high technical reproducibility of the TCR sequencing strategy. It also exemplifies that the immune microenvironment may vary between metastatic lesions from the same cancer within the same organ. In the SCLC and NSCLC comparison study, SCLC exhibited greater intratumoural variability (MHI<0.2) compared to NSCLC (MHI>0.8) [31]. This is concordant with the clonality parameter analysis, and points to NSCLC having a more homogenous neo-antigen landscape than SCLC. An interesting follow-up study in our cohort would be to extend the analysis of pairwise comparison of CLM samples resected from the same patient by increasing the number of cases included.

B cells, in contrast to T cells, undergo somatic hypermutation. Previous studies of network repertoires generated from analysis of plasma cells show that B cells may exhibit highly centralized networks, with one clone highly connected to a large number of peripheral (but very similar) clones. The degree distribution of such networks follows a power law function, suggesting reactivity towards a single antigen [17,22]. Very few of the TCR networks generated from our CLM cohort showed evidence of such sequential centralization. Instead, a small number of public clones (<10% of the detected clones) accounted for the majority of network connectivity. This is in line with prior findings from analysis of murine and human TCR repertoires [33], and suggests the existence of a small number of CDR3β sub-sequences that, although composing only a fraction of the entire clonal landscape, are overrepresented in the repertoires of many patients, but also within individual patient repertoires. The finding is also congruent with the finding that a high proportion of the highly connected and public clones were linked to known pathology-associated TCRs in the McPAS database (fig. 3g). Among the most frequent pathologies included influenza, tuberculosis, colorectal cancer, cytomegalovirus and epstein barr virus. Some of these TCR sub-sequences could therefore be conserved in the Norwegian population due to common vaccination or viral exposure [34].

An alternative possibility, more specific to this cohort, is that they could represent a subset of T cells recognizing common tumour associated antigens, or neoantigens, that are conserved in CRC, and these sequences could be potential candidates for further analyses [35,36]. A third possibility is that the sequence convergence is generated by biases in V(D)J recombination [37,38].

Although statistically significant, the differences between the NACT exposed and non-exposed tumours with respect to T cell infiltration and clonality were moderate, imposing limitations to the interpretation of the data. The methodological approach was also not able to distinguish between different T cell subsets. The observed differences in T cell infiltration seem to be driven by a subgroup of tumours that had a strong T cell response to NACT in the short-interval group. Given that all the included patients had microsatellite stable disease [9], such responses could indicate a CLM subgroup that is immunologically interesting with respect to the response to chemotherapy. For further studies, the time interval between chemotherapy exposure and TCR analysis should be standardized, as we have done in the ongoing METIMMOX trial (NCT03388190), where cytotoxic chemotherapy is administered sequentially with ICI in microsatellite stable mCRC. Although TCR sequencing data suggests the presence of NACT-driven clonal expansion, further exploration of the sequential makeup and possible sequential convergence of antigen binding TCR clones is warranted [39].

Conclusions

Analysis of TCR repertoires in CLM confirmed our previous finding that NACT exposure was associated with a transient increase in T cell infiltration, while a more persistent increase in clonality was observed independently of the timing of NACT administration and liver resection. The findings are consistent with a chemotherapy-driven clonal expansion and T cell response, possibly to tumour neoantigens. The included samples represent an excellent starting point for further studies to identify potential public and private antigenic drivers. The results underline the importance of attention to the timing of drug administration in combination trials,

and the standardized, high-throughput workflow supports the inclusion of TCR sequencing analysis in immunotherapy trials.

Availability of Source Code

Project name: airr_tools

Project home page: https://github.com/eirikhoye/airr_tools

Data and code DOI: 10.5281/zenodo.7614598

Operating system(s): Platform independent

Programming language: R 4.0.5 or higher, and python 3.6 or higher

Other requirements: r-tidyverse 1.2.1, r-alakazam 1.0.2, r-bolstad2, r-ggpubr 0.4.0, imnet, pyspark, findspark

License: Open Source

Data availability

The data sets supporting the results of this article are available in the Adaptive immuneACCESS repository, [DOI to be added upon acceptance, see below for temporary link]. Bioinformatics pipeline, code, metadata and statistical analysis can be found at GitHub at https://github.com/eirikhoye/airr_tools.

FOR REVIEWERS:

Temporary link to Adaptive repository

clients.adaptivebiotech.com

email: hoye-review@adaptivebiotech.com

password: hoye2022review

Abbreviations

ICI: Immune checkpoint inhibitor

CDR3: Complementarity determining region 3

TCR: T cell receptor

CRC: Colorectal cancer
CLM: CRC liver metastasis
MSS: Microsatellite stable
NACT: Neoadjuvant chemotherapy
PCR: polymerase chain reaction
LD: Levenshtein distance
MHI: Morisita Horn index
CI: Confidence interval
SCLC: Small cell lung cancer
NSCLC: Non-small cell lung cancer

Ethical Approval

Written informed consent was obtained from all participating patients. The OSLO-COMET study was approved by Norway's Regional Committees for Medical and Health Research Ethics (ID# 2011/1285/REK sør-øst B).

Additional Files

Supplementary file. Supplementary Figure 1. Kaplan Meier plot comparing the effect of clonality on overall survival. Estimated from time of CLM resection and censored at time of death. Supplementary Figure 2. Comparison of clonality and the location of the primary tumour.

Competing Interests

The authors declare no conflict of interest exists.

Funding

South-Eastern Norway Regional Health Authority (grant#2018014/K.F.), Norwegian Cancer Society (grant#215817/V.G.)

Author’s Contributors

E.H.: data curation, formal analysis, investigation, methodology, software, visualization, writing – original draft; V.J.D.: conceptualization, data curation, investigation, project administration, resources, writing – review & editing; A.T.: data curation, investigation, project administration, resources, writing – review & editing; C.L.A.: data curation, investigation, project administration, resources, writing – review & editing; Å.A.F.: data curation, project administration, resources, writing – review & editing; S.L.: conceptualization, resources, writing – review & editing, B.E.: funding acquisition, project administration, resources, writing – review & editing; E.H.: data curation, resources, writing – review & editing; B.F.: conceptualization, investigation, supervision, writing – review & editing; E.M.I.: conceptualization, supervision, writing – original draft; V.G.: formal analysis, methodology, supervision, writing – original draft; A.H.R.: conceptualization, funding acquisition, supervision, writing – review & editing; K.F.: conceptualization, data curation, formal analysis, funding acquisition, investigation, methodology, project administration, resources, supervision, writing – original draft.

Acknowledgements

Not applicable.

References

1. Le DT, Durham JN, Smith KN, Wang H, Bartlett BR, Aulakh LK, et al.. Mismatch repair deficiency predicts response of solid tumors to PD-1 blockade. *Science*. 357:409–132017;

2. Laumont CM, Vincent K, Hesnard L, Audemard É, Bonneil É, Laverdure J-P, et al.. Noncoding regions are the main source of targetable tumor-specific antigens. *Sci Transl Med*. 2018; doi: 10.1126/scitranslmed.aau5516.

3. Rosati E, Dowds CM, Liaskou E, Henriksen EKK, Karlsen TH, Franke A. Overview of methodologies for T-cell receptor repertoire analysis. *BMC Biotechnol*. 17:612017;

4. Brown AJ, Snapkov I, Akbar R, Pavlović M, Miho E, Sandve GK, et al.. Augmenting adaptive immunity: progress and challenges in the quantitative engineering and analysis of adaptive immune receptor repertoires. *Mol Syst Des Eng*. The Royal Society of Chemistry; 4:701–362019;

5. Bray F, Ferlay J, Soerjomataram I, Siegel RL, Torre LA, Jemal A. Global cancer statistics 2018: GLOBOCAN estimates of incidence and mortality worldwide for 36 cancers in 185 countries. *CA Cancer J Clin*. 68:394–4242018;

6. Ferlay J, Soerjomataram I, Dikshit R, Eser S, Mathers C, Rebelo M, et al.. Cancer incidence and mortality worldwide: sources, methods and major patterns in GLOBOCAN 2012. *Int J Cancer*. 136:E359-862015;

7. Cohen R, Rousseau B, Vidal J, Colle R, Diaz LA Jr, André T. Immune Checkpoint Inhibition in Colorectal Cancer: Microsatellite Instability and Beyond. *Target Oncol*. 15:11–242020;

8. Galon J, Costes A, Sanchez-Cabo F, Kirilovsky A, Mlecnik B, Lagorce-Pagès C, et al.. Type, density, and location of immune cells within human colorectal tumors predict clinical outcome. *Science*. 313:1960–42006;

9. Dagenborg VJ, Marshall SE, Yaqub S, Grzyb K, Boye K, Lund-Iversen M, et al.. Neoadjuvant chemotherapy is associated with a transient increase of intratumoral T-cell density in microsatellite stable colorectal liver metastases. *Cancer Biol Ther*. 21:432–402020;

10. Østrup O, Dagenborg VJ, Rødland EA, Skarpeteig V, Silwal-Pandit L, Grzyb K, et al.. Molecular signatures reflecting microenvironmental metabolism and chemotherapy-induced immunogenic cell death in colorectal liver metastases. Oncotarget.

11. Carlson CS, Emerson RO, Sherwood AM, Desmarais C, Chung MW, Parsons JM, et al.. Using synthetic templates to design an unbiased multiplex PCR assay. *Nat Commun*. Nature Publishing Group; 4:1–92013;

12. Amoriello R, Chernigovskaya M, Greiff V, Carnasciali A, Massacesi L, Barilaro A, et al.. TCR repertoire diversity in Multiple Sclerosis: High-dimensional bioinformatics analysis of sequences from brain, cerebrospinal fluid and peripheral blood. *EBioMedicine*. Elsevier; 2021; doi: 10.1016/j.ebiom.2021.103429.

13. Gupta NT, Heiden JAV, Uduman M, Gadala-maria D, Yaari G, Kleinstein SH. Change-O : a toolkit for analyzing large-scale B cell immunoglobulin repertoire sequencing data. 31:3356–82015;

14. Hill MO. Diversity and evenness: A unifying notation and its consequences. *Ecology*. Wiley; 54:427–321973;

15. Greiff V, Bhat P, Cook SC, Menzel U, Kang W, Reddy ST. A bioinformatic framework for immune repertoire diversity profiling enables detection of immunological status. *Genome Med*. Genome Medicine; 7:3–52015;

16. Curran J, Bolstad W. Bolstad: Bolstad functions.

17. Miho E, Roškar R, Greiff V, Reddy ST. Large-scale network analysis reveals the sequence space architecture of antibody repertoires. *Nat Commun*. 10:13212019;

18. Shannon P, Markiel A, Ozier O, Baliga NS, Wang JT, Ramage D, et al.. Cytoscape: a software environment for integrated models of biomolecular interaction networks. *Genome Res*. 13:2498–5042003;

19. Gillespie CS. Fitting Heavy Tailed Distributions: The poweRlaw Package. *Journal of Statistical Software*. 642015;

20. Amoriello R, Greiff V, Aldinucci A, Bonechi E, Carnasciali A, Peruzzi B, et al.. The TCR Repertoire Reconstitution in Multiple Sclerosis: Comparing One-Shot and Continuous Immunosuppressive Therapies. *Front Immunol*. 11:5592020;

21. Tickotsky N, Sagiv T, Prilusky J, Shifrut E, Friedman N. McPAS-TCR: a manually curated catalogue of pathology-associated T cell receptor sequences. *Bioinformatics*. 33:2924–92017;

22. Bashford-Rogers RJM, Palser AL, Huntly BJ, Rance R, Vassiliou GS, Follows GA, et al.. Network properties derived from deep sequencing of human B-cell receptor repertoires delineate B-cell populations. *Genome Res*. 23:1874–842013;

23. Page DB, Yuan J, Redmond D, Wen YH, Durack JC, Emerson R, et al.. Deep Sequencing of T-cell Receptor DNA as a Biomarker of Clonally Expanded TILs in Breast Cancer after Immunotherapy. *Cancer Immunology Research*. 4:835–442016;

24. Beausang JF, Wheeler AJ, Chan NH, Hanft VR, Dirbas FM, Jeffrey SS, et al.. T cell receptor sequencing of early-stage breast cancer tumors identifies altered clonal structure of the T cell repertoire. *Proc Natl Acad Sci U S A*. 114:E10409–172017;

25. Sankin A, Chand D, Schoenberg M, Zang X. Human urothelial bladder cancer generates a clonal immune response: The results of T-cell receptor sequencing. *Urol Oncol*. United States; 37:810.e1-810.e52019;

26. Robert L, Tsoi J, Wang X, Emerson R, Homet B, Chodon T, et al.. CTLA4 blockade broadens the peripheral T-cell receptor repertoire. *Clin Cancer Res*. 20:2424–322014;

27. Wang X, Zhang B, Yang Y, Zhu J, Cheng S, Mao Y, et al.. Characterization of Distinct T Cell Receptor Repertoires in Tumor and Distant Non-tumor Tissues from Lung Cancer Patients. *Genomics Proteomics Bioinformatics*. 17:287–962019;

28. Liu J, Yang X, Lu X, Zhang L, Luo W, Cheng Y, et al.. Impact of T-cell receptor and B-cell receptor repertoire on the recurrence of early stage lung adenocarcinoma. *Exp Cell Res*. United States; 394:1121342020;

29. Casarrubios M, Cruz-Bermúdez A, Nadal E, Insa A, García Campelo MDR, Lázaro M, et al.. Pretreatment Tissue TCR Repertoire Evenness Is Associated with Complete Pathologic Response in Patients with NSCLC Receiving Neoadjuvant Chemoimmunotherapy. *Clin Cancer Res*. 27:5878–902021;

30. Lin K-R, Deng F-W, Jin Y-B, Chen X-P, Pan Y-M, Cui J-H, et al.. T cell receptor repertoire profiling predicts the prognosis of HBV-associated hepatocellular carcinoma. *Cancer Med*. 7:3755–622018;

31. Chen M, Chen R, Jin Y, Li J, Hu X, Zhang J, et al.. Cold and heterogeneous T cell repertoire is associated with copy number aberrations and loss of immune genes in small-cell lung cancer. *Nat Commun*. 12:66552021;

32. Reuben A, Zhang J, Chiou S-H, Gittelman RM, Li J, Lee W-C, et al.. Comprehensive T cell repertoire characterization of non-small cell lung cancer. *Nat Commun*. 11:6032020;

33. Madi A, Poran A, Shifrut E, Reich-Zeliger S, Greenstein E, Zaretsky I, et al.. T cell receptor repertoires of mice and humans are clustered in similarity networks around conserved public CDR3 sequences. *Elife*. 2017; doi: 10.7554/eLife.22057.

34. Mullins CS, Linnebacher M. Endogenous retrovirus sequences as a novel class of tumor-specific antigens: an example of HERV-H env encoding strong CTL epitopes. *Cancer Immunol Immunother*. 61:1093–1002012;

35. Koesters R, Linnebacher M, Coy JF, Germann A, Schwitalle Y, Findeisen P, et al.. WT1 is a tumor-associated antigen in colon cancer that can be recognized by in vitro stimulated cytotoxic T cells. *Int J Cancer*. 109:385–922004;

36. Wagner S, Mullins CS, Linnebacher M. Colorectal cancer vaccines: Tumor-associated antigens vs neoantigens. *World J Gastroenterol*. 24:5418–322018;

37. Venturi V, Kedzierska K, Price DA, Doherty PC, Douek DC, Turner SJ, et al.. Sharing of T cell receptors in antigen-specific responses is driven by convergent recombination. *Proc Natl Acad Sci U S A*. 103:18691–62006;

38. Elhanati Y, Sethna Z, Callan CG Jr, Mora T, Walczak AM. Predicting the spectrum of TCR repertoire sharing with a data-driven model of recombination. *Immunol Rev*. 284:167–792018;

39. Chiou S-H, Tseng D, Reuben A, Mallajosyula V, Molina IS, Conley S, et al.. Global analysis of shared T cell specificities in human non-small cell lung cancer enables HLA inference and antigen discovery. *Immunity*. 54:586-602.e82021;

Variable		N (%)	median (min-max)
Gender	Male	44 (52)	
	Female	41 (48)	
Age; years			65 (57-70)
Microsatellite stable cases		85 (100)	
Primary tumor location			
	Right colon	17 (20)	
	Left colon	30 (35)	
	Rectum	35 (41)	
Overall survival (from CLM surgery); months			56 (37-79)
NACT	No	35 (41)	
	Yes	50 (59)	
Interval NACT and CLM surgery; weeks	Short-interval group	35	7 (2.9-9.5)
	Long-interval group	15	16 (9.5-25.9)

Table 1. Clinical data for cohort used in this study.
Colorectal cancer liver metastasis (CLM), Neoadjuvant chemotherapy (NACT)

Figure Legends

Figure 1. Patient cohort and T cell fractions according of neoadjuvant chemotherapy (NACT) administration groups.

a. Overview of neoadjuvant chemotherapy (NACT) administration groups. We previously determined the NACT administration cut-off level to be 9.5 weeks based on receiver operator curve analysis[9]: The no-NACT group did not receive chemotherapy prior to liver resection, while the short- and long-interval groups received NACT 2.9 – <9.5 and ≥9.5 – 25.9 weeks prior to liver resection, respectively.
b. The mean T cell fraction was higher in the short-interval group than in the no-NACT group. P-values are from two-sided Welch t-test.

Figure 2. Hill diversity and evenness profiles according to neoadjuvant chemotherapy (NACT) administration group

a. Hill diversity profiles for 77 individual datasets by mean (solid line) with 95% confidence interval (shaded area), with q values between zero and ten. The left part of each curve (q=0) represents the number of unique clones present in the repertoire. The further and more rapidly each curve drops at increasing values of q, the more uneven the clonal frequency distribution (more clonal).
b. Hill evenness profiles illustrated by mean (solid line) with 95% confidence interval (shaded area). Evenness profiles are diversity profiles normalized by the number of unique clones present in the repertoire.
c. Boxplot of TCR repertoire clonality, defined by 10 – AUC of evenness profile. The middle bar denotes median clonality. Box represents first and third quartile, while whiskers represent minimum and maximum values. P-values are from two-sided Welch t-test.
d. Linear regression of T cell clonality and the number of T cells (red line) shows a modest correlation between clonality and a high number of intratumoral T cells. Shaded area

is the 95% confidence interval of the linear regression intercept and slope. Number of T cells is determined from the total number of rearranged DNA templates detected by sequencing.

Figure 3 Network analyses

a. The network connectivity fraction (number of edges to number of nodes) plotted against the number of nodes. A linear regression model was fitted to the data (red line), and the equation parameters are shown top left. The connectivity of the networks increased exponentially with the network size. Shaded area is the 95% confidence interval of the linear regression intercept and slope.
b. Plot of p-values from goodness of fit test to a power law function of TCR repertoire network degree distributions. A p-value greater than 0.1 was considered to indicate power law distribution.
c. Barplot of the number of clonal connections (degrees) against the number of patients in which a clone was detected. Error bars represent 95 % confidence interval.
d. Barplot of the percentage of public versus private clones across all datasets.
e. Comparison of network connectivity generated from TCR repertoires stratified into public and private clones, randomly subsampled to 1000 clones per network (because of the low abundance of public clones). The middle bar denotes the median, while the box represents the first and third quartile, and whiskers represent minimum and maximum values (values outside this range reflect outliers).
f. TCR repertoire similarity assessed using the Morisita-Horn index (MHI). MHI gives an estimate of the clonal similarity between two repertoires, weighted by clonal frequency, ranging from 0 (no similarity) to 1 (identical). The figure shows TCR repertoire similarity for the entire cohort, for datasets from different CLM from the same patient (n=4), and for datasets from analysis of two tissue aliquots from the same CLM (n=3). The middle

bar denotes the median, while the box represents the first and third quartile, and whiskers represent minimum and maximum values.

g. Fraction of TCRs that were detected in the McPAS pathology associated TCR database. For each repertoire, TCR clones were stratified into two groups, the top 10% most highly connected (degree) and public (sharing level) clones, and the top 10% most expanded clones. The fraction was calculated from the number of clones with McPAS hits at Levenshtein distance of 0 in each group, divided by total number of clones among the top 10% in each group. The middle bar denotes the median, while the box represents the first and third quartile, and whiskers represent minimum and maximum values (values outside this range reflect outliers).

Figure 4 Visualization of representative CDR3β sequence connectivity networks. Each node (dot) corresponds to a unique T cell CDR3β amino acid sequence, while connections are made between nodes when LD=1. The size of each node corresponds to its relative clonal abundance, while the colour scale represents the sharing level of the clone, dark blue are clones unique to the individual patient, while yellow represents clones present across a large number of patients. a and b are networks selected from the short-interval (a) and no-NACT (b) groups. The two networks exhibit similar TCR parameters with respect to numbers of T cells and unique clones, as well as number of connections and connectivity fraction, but exemplify opposite ends of the clonality spectrum. (c) is a network from the short-interval group exemplifying a network with a very high number of T cells and unique clones.

Figure 1

Click here to access/download;Figure;fig1.pdf

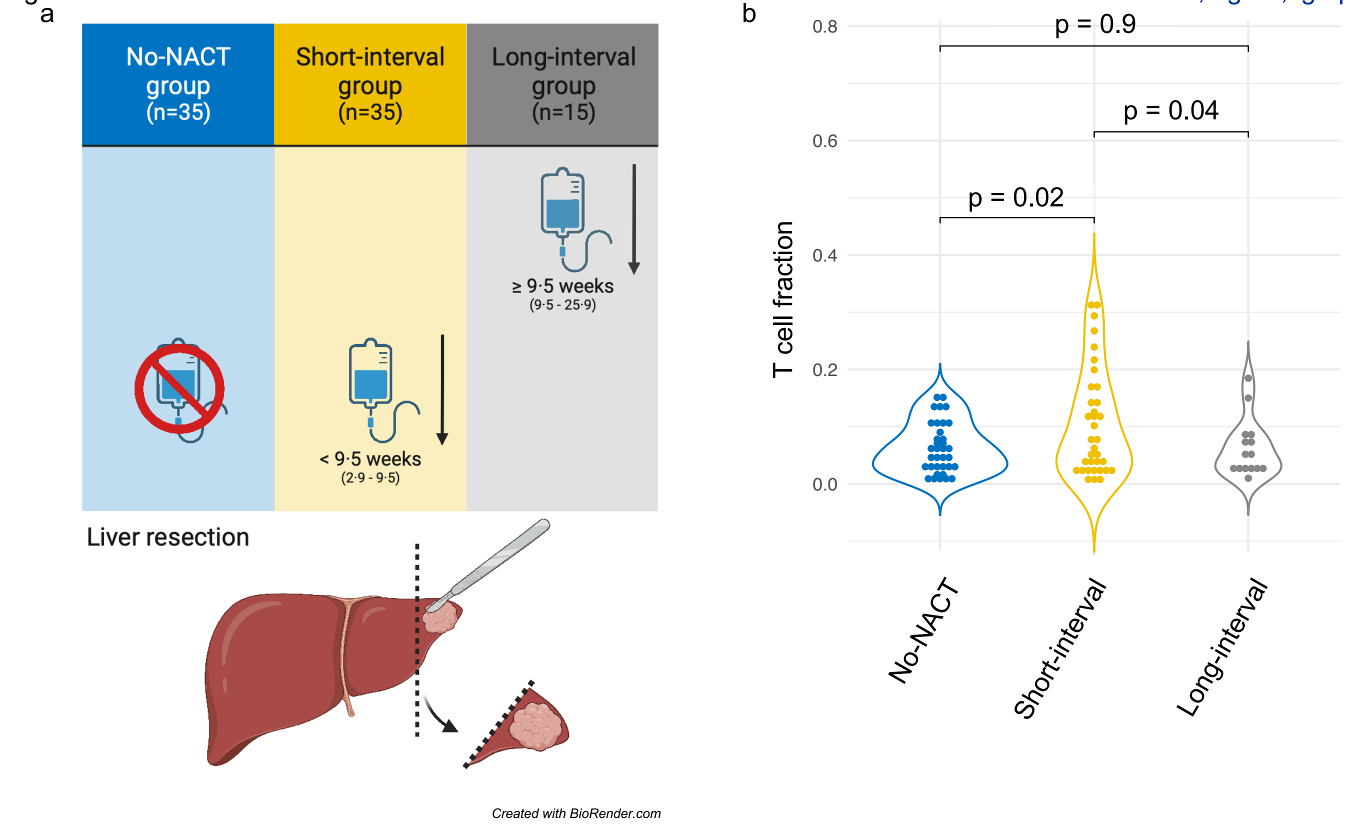

Figure 4

Click here to access/download;Figure;fig4.pdf

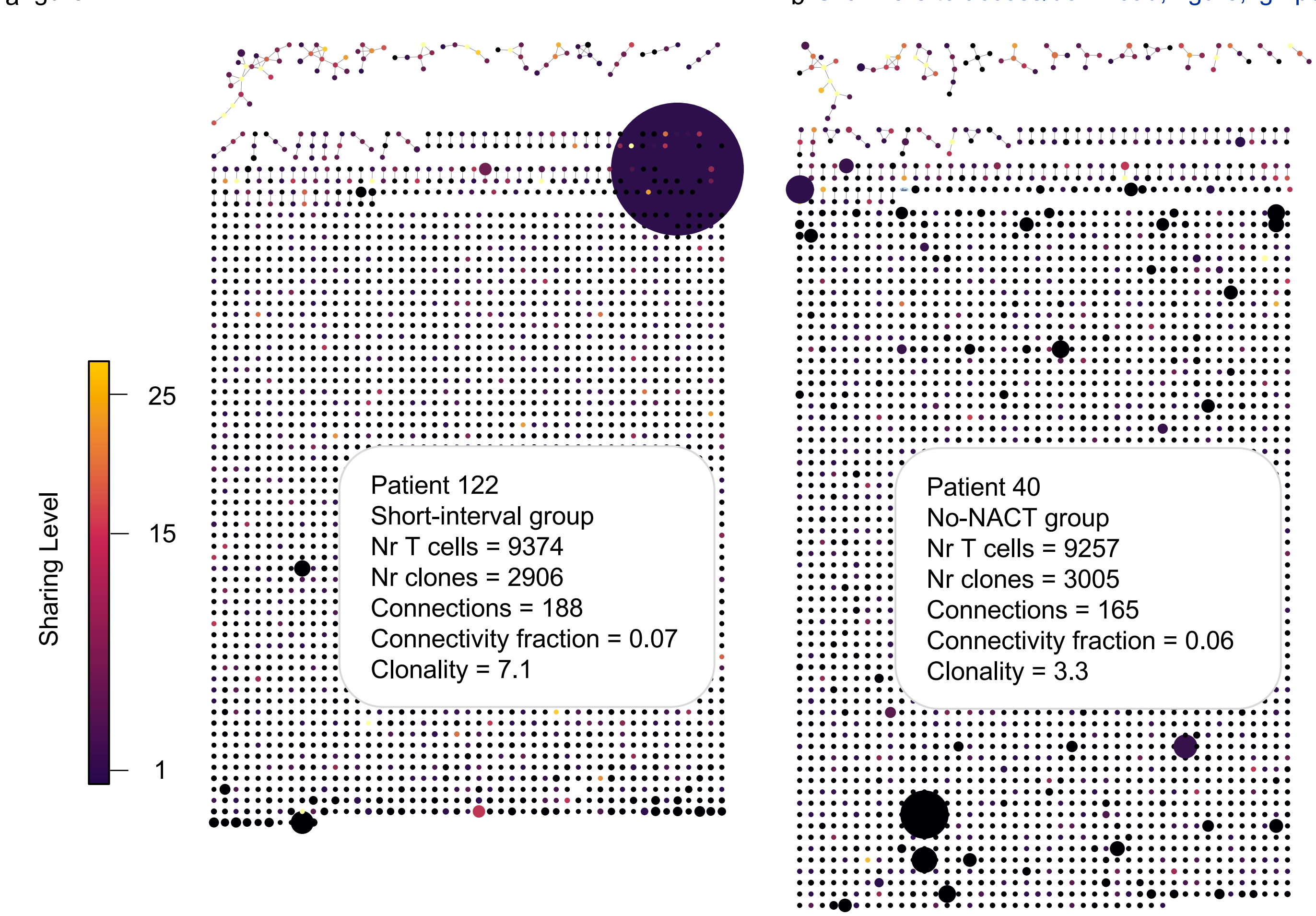

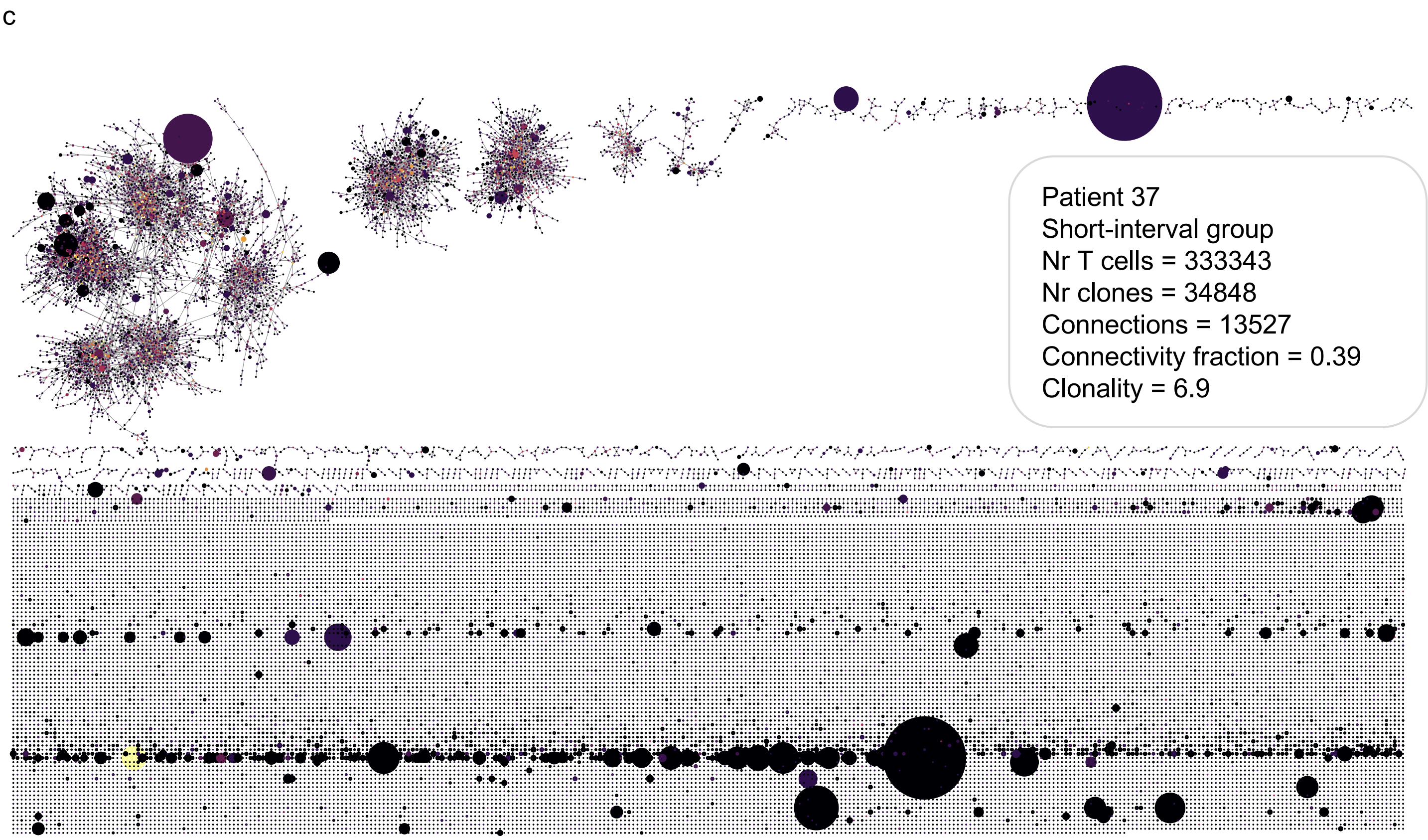

Figure 3

Click here to access/download;Figure;fig3_v3.pdf

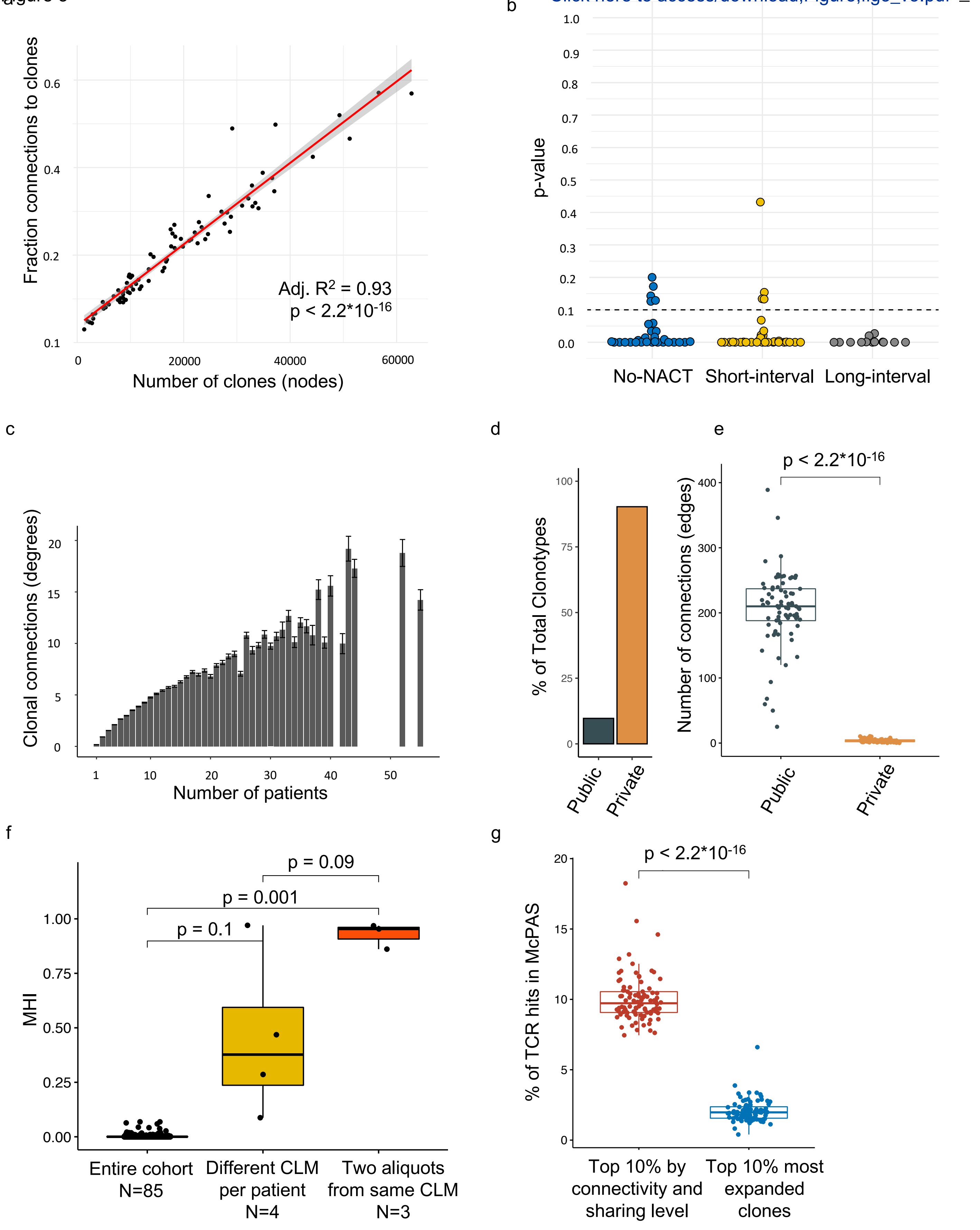

Figure 2

Click here to access/download;Figure;fig2.pdf

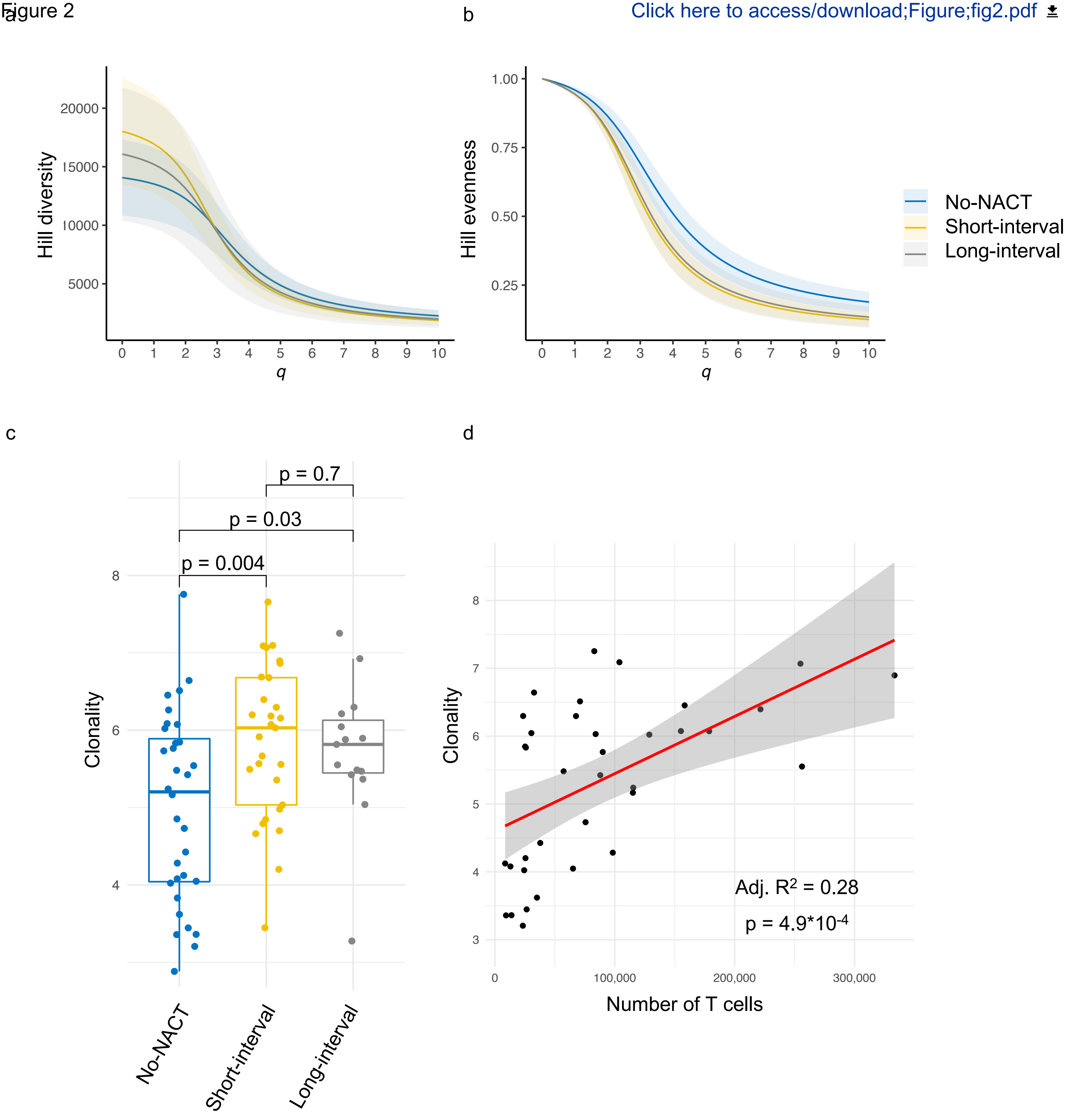

R markdown figures and statistical analysis

Click here to access/download
Supplementary Material
figure_r_code_v3.html

Click here to access/download
Supplementary Material
Supplementary_file_revised_v1.docx

Click here to access/download;Personal Cover;Cover letter.docx

Oslo University Hospital

Norwegian Radium Hospital
Oslo University Hospital
Postal address:
Trondheimsveien 235
NO-0514 Oslo
Norway

Switchboard:
+47 915 02770

Org.number:
NO 993 467 049 MVA

www.oslo-universitetssykehus.no

Oslo, September 28, 2022

The Editorial Office,
GigaScience

Dear Editor,

Please consider the enclosed manuscript entitled “T cell receptor repertoire sequencing reveals chemotherapy-driven clonal expansion in colorectal liver metastases” by Høye and co-authors for publication in *GigaScience*.

Cytotoxic chemotherapy is hypothesized to induce immunogenic cell death (ICD), causing immune activation, but evidence from the clinical setting is very limited. Colorectal cancer is typically a non-immunogenic cancer with poor responses to immune checkpoint inhibition (ICI); yet, T cell infiltration has been shown to be positively correlated with overall survival and response to immunotherapy. Our group previously identified a transient increase in tissue T cells in colorectal liver metastasis (CLM) samples from patients exposed to neoadjuvant chemotherapy (NACT).

In this work we sequenced and characterised T cell receptor (TCR) repertoires in colorectal liver metastasis (CLM) resection samples from 85 patients. Analysis of TCR repertoires again suggested a transient increase in T cell infiltration after NACT administration, but at the same time, a persistent increase in clonality was observed. These findings are consistent with a chemotherapy-driven clonal expansion of T cells, possibly because of ICD and exposure to tumour associated antigens. Being one of very few studies that is based on analysis of clinical samples, this work therefore represents an important contribution to understanding the role of chemotherapy in immune activation in metastatic colorectal cancer. The results furthermore reemphasise why timing is an important consideration when combining administration of ICI and chemotherapy in clinical trials, and point to TCR sequencing as a powerful tool for analysis of T cell infiltration and clonality in cancer research.

Because the topic is of high relevance, we hope that the manuscript will be of interest to the readership of *GigaScience*. The use of open science practices in this work facilitates reproducibility, and the new datasets will be fully accessible to researchers for integration into future research projects. We are looking forward to your response.

Yours sincerely,

Kjersti Flatmark, MD, PhD, (corresponding author)
Professor of Surgery
Research Group Leader, Department of Tumor Biology
Consultant Surgeon, Department of Gastroenterological Surgery
