# Supplementary material for: T cell receptor repertoire sequencing reveals chemotherapy-driven clonal expansion in colorectal liver metastases
Source: Gigascience. 2023 May 10;12:giad032. doi: 10.1093/gigascience/giad032 (PMC10170408; doi:10.1093/gigascience/giad032)
Supplement: giad032_Supplemental_File [file giad032_supplemental_file.docx]

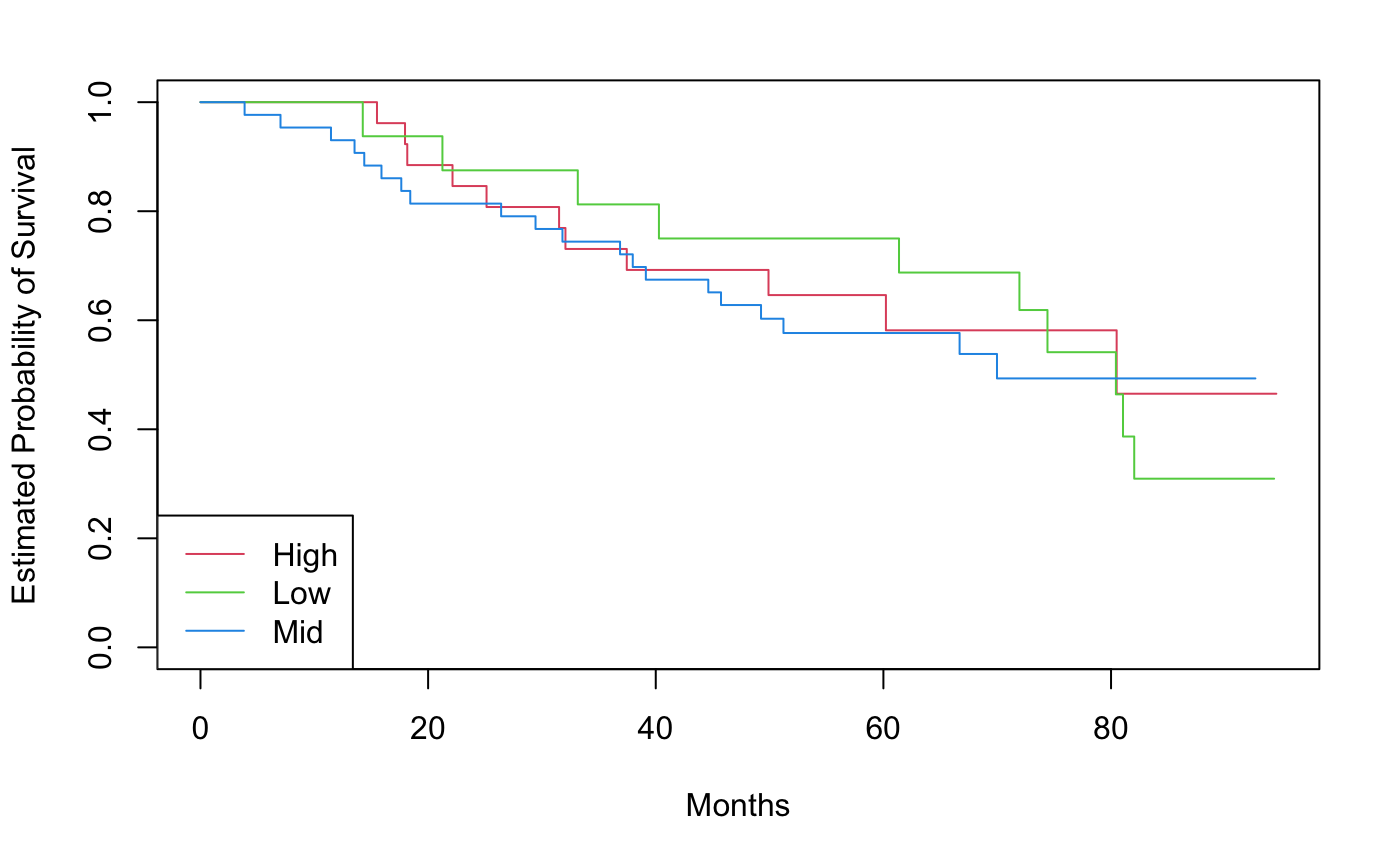


**Supplementary Figure 1.** Kaplan Meier plot of overall survival. The three curves are patients stratified into three levels of clonality, low, medium and high. Overall survival was estimated from time of CLM resection and censored at time of death.

Log-rank test p-value = **0.9**

**Supplementary Figure 2.** Comparison of clonality against the location of the primary tumour.

**Supplementary Figure 3**. Association between **a** sequencing coverage and T cell fraction, **b** ng gDNA and T cell fraction, and **c** ng DNA and sequencing coverage. Red dots are samples with T cell fraction > 0.15. Dotted line is the minimal cut-off point for sequencing coverage (5).

**Supplementary Figure 4**. Comparison of Hill based clonality estimate and sequencing coverage. Dotted line is the minimal cut-off point for sequencing coverage.

Linear regression intercept = **5.74**; Slope = **-0.015** (95% CI: -0.039 – 0.009); p-value = **0.2**.

| McPAS Pathology association | Count | Frequency |
| --- | --- | --- |
| Tuberculosis | 10503 | 29.02% |
| Influenza | 8552 | 23.63% |
| Colorectal cancer | 3784 | 10.45% |
| Cytomegalovirus (CMV) | 3518 | 9.72% |
| Epstein-Barr virus (EBV) | 2802 | 7.74% |
| Neoantigen | 1415 | 3.91% |
| Diabetes type 1 | 936 | 2.59% |
| Celiac disease | 931 | 2.57% |
| Yellow fever virus | 585 | 1.62% |
| Inflammatory bowel disease | 524 | 1.45% |
| Human immunodeficiency virus | 372 | 1.03% |
| Alzheimer’s disease | 284 | 0.78% |
| Melanoma | 277 | 0.77% |
| Psoriatic arthritis | 244 | 0.67% |
| Allergy | 236 | 0.65% |
| Parkinson disease | 139 | 0.38% |
| Rheumatoid arthritis | 125 | 0.35% |
| HTLV-1 | 124 | 0.34% |
| Clear cell renal carcinoma | 120 | 0.33% |
| Tumor associated antigen | 108 | 0.30% |
| Multiple sclerosis | 90 | 0.25% |
| Toxic epidermal necrolysis | 87 | 0.24% |
| Hepatitis C virus | 75 | 0.21% |
| Ulcerative colitis | 66 | 0.18% |
| Breast cancer | 65 | 0.18% |
| Narcolepsy | 49 | 0.14% |
| Merkel cell carcinoma | 42 | 0.12% |
| Carcinoma | 26 | 0.07% |
| Epithelial ovarian cancer | 22 | 0.06% |
| Lung cancer | 20 | 0.06% |
| Calcified aortic stenosis disease | 16 | 0.04% |
| Pollen allergen | 15 | 0.04% |
| Hepatocellular carcinoma | 8 | 0.02% |
| Hepatitis E virus | 8 | 0.02% |
| IgG4-related disease | 7 | 0.02% |
| Acute myeloid leukemia | 7 | 0.02% |
| Covid | 6 | 0.02% |
| Lymphoma | 2 | 0.01% |
| Herpes simplex virus 2 (HSV2) | 2 | 0.01% |
| Nickel contact dermatitis | 1 | 0.003% |
| Cervical cancer | 1 | 0.003% |

**Supplementary table 1**. Clonal amino acid hits for pathogens listed in the McPAS database, for top 10% most public and most connected clones.
