# Supplementary material for: T cell receptor repertoire sequencing reveals chemotherapy-driven clonal expansion in colorectal liver metastases
Source: Gigascience. 2023 May 10;12:giad032. doi: 10.1093/gigascience/giad032 (PMC10170408; doi:10.1093/gigascience/giad032)
Supplement: giad032_Response_to_Reviewer_Comments_Original_Submission [file giad032_response_to_reviewer_comments_original_submission.pdf]

Author's Response To Reviewer Comments

Close

Reviewer reports:

Reviewer #1: In this study Høye describe an analysis of the T cell receptor (TCR) repertoires of tumours from patients with colorectal liver metastases (CLM), who received different neoadjuvant chemotherapy (NACT) regimes. Their results agreed with their prior results from another cohort with orthogonal techniques: it appears that a short-term treatment (<9.5 weeks) associates with a increased T cell infiltration relative to longer intervals, and that repertoires of NACT-treated tumours in general showed greater clonality, indicative of clonal expansion.

It is an extremely well written and plotted manuscript, employing sensible and robust analyses on a reasonably-sized cohort of patients. Use of the T cell fraction in particular is very appealing potential biomarker that could be used in the adoption of repertoire sequencing for a greater range of clinical conditions, while still supplying the additional information repertoires bring. The data and code were also made available to the reviewers, and there's every indication that these will be suitably available upon publication. I am very happy recommending its publication, as I think it will be of use to the field. I have noted a small number of areas that caught my attention for the authors' consideration: several are places where some additional plotting may help reduce the possibility that the results are confounded, while the rest are minor typographical comments or similar.

Reply:
We extend our heartfelt gratitude to Reviewer 1 for the excellent and very thorough review, and for the constructive comments that improved the manuscript. See below our replies to this very detailed and excellent review.

1) I have a couple of queries about the T cell fraction. While none are major, given the importance of the metric to the study these are probably worth addressing:

a. L143 states that the number of unique CDR3b sequences is used in its calculation. Could the authors please clarify in the text to specify whether different recombinations (potentially using different V/J genes) producing the same CDR3s are grouped together in these calculations?

Reply:
In the event of different V/J gene recombination resulting in the same rearrangement sequence, these were not grouped together, and counted separately. T cell fraction was calculated by the sum of rearranged DNA templates detected in each sample (a proxy for the number of T cells detected), divided by the total number of genomes (calculated from the amount of input gDNA in the first library preparation mix). Counting the same sequential rearrangement produced from different V/J gene rearrangements separately or together will therefore not make a difference on the T cell fraction calculation. The lines 144-146 have been adjusted to make this clearer.

b. Given that the T cell fraction is calculated using the number of TCR rearrangements per sample, there's a chance that it might be confounded by differences in sequencing depth or errors, which are both in part dependent on the amount of input DNA. To rule these possibilities it might help the reader to include plots showing a lack of correlation between T cell fraction and number of productive TCR rearrangements, and between T cell fraction and amount of input gDNA. Additionally randomly selecting a matched number of sequences per donor prior to metric calculation can help rule out the possibility that these variables might be confounding (similar to how higher q values remove the effects of small clones in the later diversity metrics).

Reply:
Regarding T cell fraction and sequencing depth, all samples were sequenced with the same number of PCR replicates, which is what determines sequencing depth. Sequencing coverage in the Adaptive Biotechnologies ImmunoSEQ platform is calculated as the average number of reads per rearranged DNA template (proxy for the number of T cells) prior to PCR amplification. This number is possible because of the presence of synthetic rearrangements diluted to be present at either 0 or one template in library preparation mix prior to PCR amplification. Sequencing coverage is therefore expected to decrease as the number of T cells present in the tissue increases.

The below plots show the relationship between coverage, input gDNA in the library preparation mix and T cell fraction.

Points were colored based on whether the T cell fraction was greater than 0.15. Dashed lines represent sequencing coverage of 5.

a. Samples with very high T cell fraction leads to low sequencing coverage.
b. There was no correlation between T cell fraction and input gDNA.
c. Nor was there a strong correlation between gDNA and coverage.

All biological samples were run using two PCR replicates. The ImmunoSEQ protocol is optimized for 30 000 to 45 000 rearranged T cell genomes per PCR replicate. The median number of productive rearranged DNA templates (proxy for number of T cells) per PCR replicate in this dataset was 31 532, well within the optimal range. However, among the samples with high T cell fraction, the number was much higher, the maximum being 166 672 rearranged templates. The low coverage for these samples seen in plot a was likely caused by oversaturation of T cells. Ideally, the input DNA in these samples would have been reduced prior to library preparation, so as to have the optimal T cell number per PCR replicate. However, the T cell content for each tissue was not known prior to sequencing. As can be seen from plot b, T cell fraction was not impacted by variation in input gDNA, nor was coverage (plot c).

For T cell fraction analysis, the lower coverage and oversaturation of T cells for CLM tissue with very high T cell content is not of major concern. Potential bias likely reflects an underestimation. However, for clonality analysis low coverage is a major concern, resulting in a bias towards abundant clones. Therefore, samples with low coverage (< 5) were dropped from the downstream clonality analysis in order to ensure unbiased analysis.

The below plot show that there was no association between clonality and coverage.

Linear regression (clonality ~ coverage) intercept = 5.74; slope = -0.015 (95% CI: -0.039 – 0.009); p-value = 0.2. Clonality was evenly distributed over the coverage distribution, and the slight negative trend in clonality for increasing coverage was likely random noise. This shows that Hill evenness based clonality estimates are robust both to variation in absolute T cells, and also robust to variations in sequencing coverage.

These figures have been added to the supplementary file. Lines 135-136 of the manuscript have been updated to make the rationale for input gDNA per PCR replicate clearer.

c. Re: Fig 1: while the range of interval length is given, the distribution is not. I wonder if perhaps a (potentially supplementary) plot of duration of interval vs T cell fraction would be instructive. This would also be a potentially independent useful validation of the use of 9.5 mo as a threshold, which it seems was determined by the authors in a significance screen by ROC analysis in their prior publication. As the discussion remarks on the agreement between the results of this study broken by interval length and that previous publication, the authors may also wish to consider repeating that ROC analysis on these data, to see if a similar threshold is produced.

Reply:
We agree that it would be valuable to have an independent validation of the 9.5 months cutoff, however the material produced in this study is not suitable for this task. Samples were selected with the purpose of acquiring sufficient samples in each NACT-interval group for meaningful statistical analysis. The time interval distribution is not independently sampled, and would not be suitable for statistical analysis. The minimum and maximum interval in weeks for the two groups was already described on L117, but we have also added the median.

2) The Hill diversity/evenness data beautifully plotted in Fig 2 a/b is a robust way to illustrate these 'species'-level metrics.
a. However in my experience it can't be expected that the reader will know how to interpret these plots: even many people who study repertoire data are unfamiliar with these more ecological measures. I imagine this is perhaps even more true of the oncologists who may be interested in this study. I wonder if perhaps an explanatory sentence or two in the legend might help? I know there is some of this in the discussion, but that's likely going to be seen after the figures for many readers.

Reply:
Additional explanatory text has been added to the relevant legends. Updated figure 2 a and b legend text.

b. Also I think maybe the x axes would more accurately be labelled 'q values' (rather than 'α').

Reply:
This is correct, we apologize for this mix-up. We have altered the x axis label to q. Figure 2 a and b have been updated.

d. Some other values can be guessed at but aren't specifically defined in the legend, e.g. it only defines the median for Fig 2c and not the whisker/boxes, while the shaded confidence interval area in 2d is similarly not defined. It's also unclear where the T cell count information is coming from in this plot: were orthogonal T cell count data collected, or is this inferred from the repertoires?

Reply:
The relevant figure legends have been updated to include these points. Figure 2 d legend text has been updated.

3) The network analyses of Fig 3/4 are interesting, but currently presented in a way was harder to intrepret than surrounding sections, with several fairly field-specific analyses done with little explanation.
a. The text results of this analysis (beginning on L257. "The mean number of nodes detected was...") is abrupt, discussing results of an analysis that hasn't been established yet. This continued through the paragraph. E.g. it's unclear why the test for power law was performed: while it is well known that clonal repertoires tend to follow such distributions, the authors don't state why they think the network connectivities should.

Reply:
This is a fair point, and we agree that this section may be somewhat abrupt. Our rational for this analysis was to see whether there may possibly be a convergence of TCR sequences based on shared specificity of antigen epitopes. However, in this case there was no evidence for such a convergence. We have updated the text (L259 – L261). We have also added a sentence on the background for why we conducted a test for whether the degree distribution followed a power law distribution. We have updated the text (L266 - L268) to make the purpose and our prior expectations clearer.

b. Following on from my point (1) above, a common confounder in such connectivity networks is the possibility for sequencing and PCR errors to artificially inflate networks. The incidence of such errors could be biased towards certain samples, depending on factors like gDNA input and number of PCR cycles. You would also expect more abundant clonotypes on average to produce greater numbers of erroneous sequences, as a function of their occupancy of the reactions involved, which could contribute to the

correlation seen in Fig 3a. Some control analyses or discussion might be useful, to help discount this possibility (with other plots in this figure lending weight to the notion that this is not artefactual).

Reply:
This is a fair point, and we have added another sentence explaining why these sequence convergences occur (L388-390). However, this does not alter the primary conclusion of this manuscript. The sequence convergence in the network analysis were mainly composed of singleton or low abundance clones. Expanded clones were, in contrast, sequentially distinct and disconnected from the other clones in the network analysis, as can be seen in figure 4. The finding from this study therefore suggest that the T cell infiltration is driven by a heterogenous neoantigen landscape, unique to each patient/tumor.

c. Again there are some missing value definitions: 3a shaded area, box/whisker markers in e-g. The 3f y axis label is also mislabelled: 'MOI' should likely be 'MHI'.

Reply:
Figure 3 legend have been updated to include these definitions, and Figure 3f axis label has been changed to MHI. We thank the reviewer for making us aware of these omissions.

4) It is also probably worth noting somewhere in the text that the connectivity difference between public/private sequences is exactly what one would expect given our understanding of biased V(D)J recombination (which underlies the production of public sequences in the first place). https://doi.org/10.1073/pnas.0608907103 would be a classic citation for this, with more recently validations using higher-throughput TCRseq data discussed in https://doi.org/10.1111/imr.12665.
a. It has also similarly been observed that many T cells detectable in tumours are specific for viruses in both humans and mice, e.g. https://doi.org/10.1038/s41586-021-03704-y, https://doi.org/10.1172/JCI150535, and https://doi.org/10.4049%2Fjimmunol.1601064.

Reply:
As mentioned above, we have added another sentence (L388-390) that makes this point, and included the mentioned references. Furthermore, the results from the analysis of TCR sequences present in the McPAS database revealed that the most common pathological associations included influenza, tuberculosis, cytomegalovirus and epstein barr virus. This has already been mentioned in the text. In regards to the conclusion, we think that it is significant that, as can be seen in fig3g, the most expanded clones also had fewer hits in the McPAS database. This suggests these expanded clones are likely specific to private neoantigens.

5) As noted above, both the data and code are available to the reviewers, which is great. However there are a few things that could be changed to align more with the reproducibility and data reuse this journal prides itself on.
a. The Github repo seems to have had a working directory uploaded to it, along with the associated detritus that such folders generate. There are several scripts and tables that seem to be derived from each other (eg X, X_v2), and at least one directory of what I suspect is analyses that didn't make it to the manuscript ('gliph2'). Tidying up these redundancies and not needed files would make the repo more navigable.

Reply:
We have tidied up the repository to make it easier to navigate for other researchers. Metadata files have been merged and cleaned up, and a more fleshed out README.md file should make the repository more navigable.

b. Similarly, while many of the scripts are sensible named, commenting is sparse and there's no summary or record of what metadata is recorded where and what scripts do what. While I'm sure the researchers running the analyses know exactly how to navigate this repo, anyone wishing to repeat or reapply these analyses will not, so an expanded README or similar would help a lot. This would be especially useful for the core pipeline of the study, and the key metadata values (e.g. Adaptive sample ID, patient IDs for those with >1 sample, treatment status/interval, ng DNA etc).

Reply:
The README.md file in the repository has been fleshed out to make it clearer. All metadata has been cleaned up so that there are now only three files:
- SampleOverview.tsv (Sequencing metadata and input gDNA)
- metadata.xlsx (clinical metadata and also show which samples were from patients sequenced more than once)
- qcReport.tsv (mainly for sequencing coverage)

c. It looks like the journal's policies call for DOIs for code as well as data: I don't know about their provided Code Ocean option, but generating a Zenodo DOI for a GitHub release is very straightforward, and makes sense seeing as the code is already there.

Reply:
This is a great suggestion; we have generated a Zenodo DOI to the github repository:
DOI: 10.5281/zenodo.7614598
6) A few of the axis tick label fonts are small relative to the rest of the figure (e.g. Fig 3a/c), so making these larger would help the short sighted readers like myself from having to frequently zoom in.

Reply:
The font sizes in Fig 3a/c have been increased.

7) L202-204, sample exclusion. It's not entirely clear to me what "sequencing coverage <5" means here: fewer than five different TCRs, or counts, or something else? In any case it should be stated which of the three test arms (no-NACT/short/long) they fell from.

Reply:
Sequencing coverage was calculated as the mean number of reads per rearranged DNA template in the original library prep mix prior to PCR amplification (which was used in this study as the proxy for number of T cells, as described above). This number can be calculated from post PCR sequencing data because each library preparation mix also contain synthetic rearranged DNA templates diluted to be present at either 0 or 1 template. This allows one to count backwards the number of DNA templates present in the original mix. A general recommendation is that, at a minimum, the number of sequencing reads should exceed the clonal diversity (1). Clonal frequency distribution for all sequencing datasets in this study were at least somewhat skewed, therefore the clone to cell ration was below 1 for all samples. The <5 coverage cut-off was the recommendation from consulting with Adaptive Biotechnologies. Because it is calculated based on reads per cell, not based on reads per clone, it is well above the minimum sequencing depth recommendation. L208 has been updated to include n for each group.

8) L183: the version/accession date of when McPAS was used should be stated. It might even be worth depositing the version of the database that was used somewhere accessible (e.g. the analysis repo), as since Nir Friedman's sad passing it appears that the resource's original URL is no longer working

Reply:
This is a good suggestion, we have added the file used to query to the github repo, and have added the download date on lines L185-L186.

9) Use of the word 'monoclonal' in L287-288 ("...a very large monoclonal network (Clonality=6.9) is visualized...") seems counter-intuitive, seeing as the sentence later points out how there are tens of thousands of different CDR3s are involved.

Reply:
We have re-phrased some of the sentences in this section to make it clearer. L292, L295 and L298 have been edited.

10) L254, possible typo or unclear abbreviation: "median og y"

Reply:

Thank you for making us aware, we apologize for this mistake and have corrected the typo.

11) Several times 'sequential' is used to mean 'relating to sequences'. I think most people will be able to figure out what this means, but (at least in my specific dialect of English) 'sequential' is mostly only used to describe things which occur in a sequence, rather than describe the sequences in the abstract themselves, so this threw me each time it came up. Changing to 'sequence' (e.g. L41/42, "TCR sequential convergence" could become "TCR sequence convergence") would avoid this if the authors wished.

Reply:
L41 has been changed to TCR sequence convergence.

Reviewer #2: This manuscript by Høye et al analyzed the effects of NACT on the TCR repertoire of TIL in CLM. Using several unique approaches of data analysis, they concluded that NACT increased the T cell infiltration and clonal expansion. Overall, data and conclusion seems to be convincing. The results that "shared clones have higher connectivity of TCR sequences" and "shared clones are enriched with antigen-specific TCRs registered in the database" were novel and interesting. On the other hand, as the authors point out in the discussion, there have been similar studies on other types of cancer, and the "increase in clonality with neoadjuvant chemotherapy" is not novel. It was difficult for me to understand the contents of the Results because the indicators used to evaluate the repertoire were not popular. I think it would be better to move some of the information on the significance of each indicator and the purpose of the analysis from the Discussion to the Results, and make the Results easier to read.

Reply:
We thank reviewer 2 for taking the time and effort to review our manuscript. Both reviewer 1 and 2 have suggested to add more explanatory text to the results section. We have added sentences to lines L259-L261 and L266-L269 in order to make the purpose of the network analysis clearer, as well as expanding the fig 2a and 2b figure legends so that it is easier to interpret for readers who are not familiar with Hill diversity and evenness profiles.

Major points
1. Line 247-248: clonality is evaluated using AUC of Hill diversity profile. However, since the "1-Pielou" index is commonly used in studies on TCR repertoire analysis, it would be better to show the calculation results of 1-Pielou index as a Fig.

Reply:
While many studies on TCR repertoire analysis use 1-Pielou index, the Hill diversity profiles, in a sense, already contain the Pielou index. This is because the Hill function at q=1 is mathematically equivalent to the reciprocal of Shannon entropy, as was proven by Hill in 1973. Pielou index is derived from Shannon entropy, thus is included in the diversity profile in fig 2a. The reason for using the whole profile is because different diversity indexes can yield qualitatively different answers on which repertoire is the most diverse, thus one should assess them based on a range of diversity estimates, such as that shown in fig2a. The AUC based clonality estimate was primarily intended for making a convenient single point estimate for statistical comparison, but one should consider the entire profile when evaluating clonality, i.e. all of fig2a, b, c.

2. line 238-241: Hill diversity profile was not explained, and it was difficult to understand. It would be helpful to include the information equivalent to Discussion line302-309.

Reply:
This was also a request from reviewer 1, and we have added some additional explanatory sentences in the figure legends. Figure legend 2a and 2b have been extended to make it easier to interpret for readers who are not familiar with Hill diversity and evenness profiles.

3. Fig. 2 claims an "increase in Clonality with neoadjuvant chemotherapy". Although this study did not separate CD4+ T cells from CD8+ T cells in the TCR repertoire analysis, it has been reported that CD4+ T

cells have higher Clonality than CD8+ T cells in their repertoires (Gueguen et al. 2021, Zhang et al. 2018). Therefore, the increased Clonality of tumor T-cell repertoires reported in this study may only reflect an increased proportion of CD8+ T cells in tumor-infiltrating T cells (Rudqvist et al. 2018). This point should be mentioned as a limitation in Discussion.

Reply:
This is a fair point, and we have added a sentence to the limitations part of the discussion, L394-L395. We agree that this is a limitation, however Dagenborg et al, 2020 (2) found that, using immunohistochemistry on a subset of the same cohort, both CD4+ and CD8+ T cells had higher infiltration in the short-interval group compared to the no-NACT and long-interval group.

4. Line 257-259: I couldn't figure out why the authors performed network analysis just by reading the result. Please include something like Discussion line 359-363 in the result.

Reply:
This was also requested by Reviewer 1, we have expanded this section to explain the purpose of these analysis, L259-L261.

5. Line 278-279: Please provide a table with a list of target pathogens (flu, tuberculosis, etc.) of the clones that matched the McPAS database. Also, please indicate what type of cancer antigens (differentiation antigen, cancer testis antigen, neoantigen, etc.) are listed as "colorectal cancer".

Reply:
The table of unique CDR3 amino acid hits to the McPAS database, for both the public and private most prevalent clones, have been added to the github repository:
- data/McPAS/public_cdr3_aa_unique_McPAS_hits.csv
- data/McPAS/private_cdr3_aa_unique_McPAS_hits.csv

Regarding McPAS, it is a database of TCR sequences that have been reported in the literature to be associated with pathological conditions. The specific antigens they respond to is not necessarily known. Our understanding of TCR to antigen specificities is limited, due to the vast diversity of both TCR sequences, and potential tumor neoantigens. Exploring TCR:antigen specificities is outside the scope of this work, and not currently possible with sequencing technology alone.

6. Line 281-290: Fig. 4 only shows an example of TCR network, so I felt that the order of presentation should be switched with Fig. 3.

Reply:
While we can understand this point of view, the information in Fig. 3 is essential for understanding Fig. 4. We therefore think the order is correct.

7. Line 340-343: "differences in clonality between NACT exposed and unexposed CLM might be of clinical relevance." I was not sure what this "clinical relevance" meant. Also, if the authors are trying to claim similarity of their tumor microenvironment based solely on the similarity between "differences in TCR repertoire index between immune cold SCLC and immune hot NSCLC" and "differences in TCR repertoire index of CLM with and without NACT", it is an overstatement.

Reply:
We believe that the difference in clonality may reflect differences in response to chemotherapy. This may be important in the context of immunotherapy in mCRC. This hypothesis, where priming the immune system with chemotherapy in a brief time interval prior to immunotherapy, is currently being tested in the randomized control trial METIMMOX (NCT03388190). Regarding the analysis of clonal overlap between repertoires, our findings (Fig. 3f) showed that there was no clonal overlap between patients, while repertoires from the same patient but sampled at different locations of metastasis in the liver showed only modest overlap. Repertoires generated from the same metastatic aliquots had almost complete clonal overlap. Our conclusion from this is again that this is suggestive of a CLM neoantigen landscape that is highly diverse and private to the individual patient. NSCLC in contrast had far higher intratumoral TCR

homogeneity, suggesting the neoantigen landscape of these tumors is shared for the majority of tumor cells. This is an important predictor for whether current immunotherapy approaches will have an effect, and may be part of the reason for the very good response in NSCLC compared to the poor response in SCLC and also in mCRC. We agree however that one must be careful comparing these studies, due to possible differences in sampling strategies, etc.

8. Line 352-355 compares the heterogeneity of TCR repertoires in tumors between NSCLC and SCLC, which seemed not relevant to the content of our paper.

Reply:
We think this is interesting in the context of potential response to immunotherapy. The more homogenous TCR repertoire in NSCLC is suggestive of a homogenous neoantigen landscape, which is an important predictor of response to immunotherapy. See also reply above.

Minor points
1. line 242: I do not understand what "increasing value of q" refers to. Is it referring to the "alpha" in Fig2 a, b?

Reply:
We apologize for this mistake, which was also pointed out by reviewer 1. The Fig. 2a and 2b x axis label have been fixed to q instead of alpha.

2. In Fig. 2b, the plot for the treated group was lower than the plot for the untreated group, but I did not immediately understand the correspondence between this and the statement "Clonality was higher in the treated group". Legend stated that "Clonality was evaluated by 10-AUC", which I think should be clearly stated in the RESULT.

Reply:
The evenness profile curves should be read as follows. If the curve follows a straight line (evenness close to 1.0) for the entire range of q values, the clonal frequency distribution is completely even (all clones are identical at the same frequency, a completely heterogenous distribution). The further and more rapidly the curve drops as the value of q increases, the more uneven the clonal frequency distribution. (i.e. the more it is dominated by a small subset of very frequent clones). Legends for Fig2a and 2b have been updated, as was also requested by reviewer 1. The 10-AUC is explained in the methods section, L157-L161.

3. line 250-251: "regression analysis revealed a modest association between a high absolute number of T cells and clonality," I could not understand what this analysis was intended to claim.

Reply:
We included this because it suggests that the higher number of infiltrating T cells is driven by clonal expansion.

4. Lines 259-260: I didn't understand what you were trying to argue with this analysis.

Reply:
We have added some explanatory text that explains the purpose of the network analysis. See L259 – 261, this was also requested by reviewer 1.

5. Line 262-264: The authors compared the clonality between repertoire that satisfy the power-law fit test and those that do not. Please provide a dot plot in addition to the average value.

Reply:
While we can understand why an additional plot might be useful, the take home message from the power law was that the networks produced in this study did not follow a power law. Prior studies on antibodies have found that the degree distribution of neoantigen experienced networks follow power law, and our rationale for conducting this analysis was that this might have been an exiting tool for assessing whether the repertoire is antigen experienced or not. However, it appears that T cell repertoires follow different

dynamics, and the results of this analysis was negative.

6. Line 264-265: It is an overstatement to conclude that “clonally expanded datasets were not associated with power law degree distribution” because there are only a few repertoires that satisfy the power-law fit test.

Reply:
As also explained above, the overall conclusion from this analysis was that TCR repertoire networks do not follow the same dynamics of antibody repertoires, and power law test are, regrettably, not a suitable tool for assessing if a repertoire is antigen experienced, in this context.

7. Line 268-270: Fig3d might be a mistake for Fig3e. Also, Fig3e does not seem to be a graph comparing "mean connectivity fractions".

Reply:
Again, we apologies for the error in figure reference, which have been corrected. Regarding Fig3e, the repertoires were randomized to 1000 clones each, therefore the numbers are equivalent to fractions which is written in the text.

8. line 270: Fig3e" seems to be a mistake for "Fig3f".

Reply:
Again, we apologies for the error in figure reference, which have been corrected.

9. Line 270-271: "in line with", but I did not understand how the discussion of public clone connectivity corresponds to the discussion of repertoire similarity between samples.

Reply:
We have rewritten this line to instead say “further illuminated by”, to make our intension clearer, L280.

10. Spelling error: line 68 "Characterisation"

Reply:
We have changed this word to characterization.

11. Fig3f, Y-axis seems to be MHI.

Reply:
Corrected, as was also mentioned by reviewer 1.

References:

1. Greiff V, Miho E, Menzel U, Reddy ST. Bioinformatic and Statistical Analysis of Adaptive Immune Repertoires. Trends Immunol. 2015;36(11):738–49.
2. Dagenborg VJ, Marshall SE, Yaqub S, Grzyb K, Boye K, Lund-Iversen M, et al. Neoadjuvant chemotherapy is associated with a transient increase of intratumoral T-cell density in microsatellite stable colorectal liver metastases. Cancer Biol Ther. 2020 May 3;21(5):432–40.

Close
