# Supplementary material for: T cell receptor repertoire sequencing reveals chemotherapy-driven clonal expansion in colorectal liver metastases
Source: Gigascience. 2023 May 10;12:giad032. doi: 10.1093/gigascience/giad032 (PMC10170408; doi:10.1093/gigascience/giad032)
Supplement: giad032_Response_to_Reviewer_Comments_Revision_1 [file giad032_response_to_reviewer_comments_revision_1.pdf]

Author's Response To Reviewer Comments

Close

Reviewer reports second revision:

Reviewer reports:
Reviewer #2:

Comment:
The authors have mostly responded appropriately, and the revised version become more readable. It would be good to make additional revisions to the following points.

Reply:
We extend our gratitude to reviewer #2 for taking the extended time to further improve our manuscript. See our revisions and explanatory comments below.

Response to
Comment:
Major 3: It would be good to state more clearly in the discussion that "CD8 has higher clonality than CD4."

Reply:
While it would very interesting if the increased clonality was CD8 compared to CD4, the methodological approach used in this study is not able to distinguish between such subtypes. We have rephrased one of the sentences in the limitations part of the discussion, L400, to make this a little clearer.

Comment:
Major 5: Please provide a table or pie chart showing the composition of TCR target antigens (Flu X%, CMV Y%, etc.) that hit the McPAS database, rather than listing them as in Lines 287-289.

Reply:
We have added a supplementary table in the supplementary file, showing the frequencies of pathology associations with TCRs in our dataset. Supplementary table 1 & L290

Comment:
Major 7: Please describe the details of Clinical relevance with specific examples.
Regarding the L352-354, please clarify that this is a "working hypothesis" of the authors and that it is "CURRENTLY BEING TESTED".

Reply:
We have expanded this section with a sentence mentioning our working hypothesis, that NACT invoke immunogenic cell death, resulting in improved responses to immunotherapy. L356-359

Comment:
Major 8: I could not understand the message of the statement until I read the reply "This is an important predictor for whether current immunotherapy approaches will have an effect, and may be part of the reason for the very good response in NSCLC compared to the poor response in SCLC and also in mCRC. effect, and may be part of the reason for the very good response in NSCLC compared to the poor response in SCLC and also in mCRC". How about including the equivalent of this in the main text?

Reply:
This is a fair point; we have rephrased the end of this section so that this message is clearly explained. L353-355

Comment:
Minor 5,6: I understood the meaning when I read the reply.
Is there a Figure corresponding to Line 266 "The connectivity fraction did not appear to be associated with the clonality of the network."?
I think the authors had better change the structure of the paragraph to make sure the following logic is conveyed.

Reply:
We did not do a separate analysis of connectivity fraction and clonality because the linear relationship seen in figure 3a showed it was purely a function of how many clones was present in the network. We changed the sentence from “The connectivity fraction did not appear to be associated with the clonality of the network” to “This observation suggest the network connectivity fraction was not associated with the clonality of the T cell repertoire frequency distributions”. L265-266

Comment:
In the BCR, the higher the clonality of a repatriate, the more the network tends to satisfy the power-low condition" (is this understanding correct?). On the other hand, in the TCR, the clonality was lower in the networks that met power-low. Thus, the authors concluded that "T cell repertoires follow different dynamics, and the results of this analysis was negative."

Reply:
We added this sentence at the end of the section: “suggesting that T cell repertoires follow different dynamics compared to B cell repertoires.” L273-274

Close
