# Supplementary material for: T cell receptor repertoire sequencing reveals chemotherapy-driven clonal expansion in colorectal liver metastases
Source: Gigascience. 2023 May 10;12:giad032. doi: 10.1093/gigascience/giad032 (PMC10170408; doi:10.1093/gigascience/giad032)
Supplement: giad032_Reviewer_1_Report_Original_Submission — James M Heather -- 11/28/2022 Reviewed [file giad032_reviewer_1_report_original_submission.pdf]

Reviewer Report

Title: T cell receptor repertoire sequencing reveals chemotherapy-driven clonal expansion in colorectal liver metastases

Version: Original Submission **Date:** 11/28/2022

Reviewer name: James M Heather

Reviewer Comments to Author:

In this study HÃ¸ye describe an analysis of the T cell receptor (TCR) repertoires of tumours from patients with colorectal liver metastases (CLM), who received different neoadjuvant chemotherapy (NACT) regimes. Their results agreed with their prior results from another cohort with orthogonal techniques: it appears that a short-term treatment (<9.5 weeks) associates with a increased T cell infiltration relative to longer intervals, and that repertoires of NACT-treated tumours in general showed greater clonality, indicative of clonal expansion.

It is an extremely well written and plotted manuscript, employing sensible and robust analyses on a reasonably-sized cohort of patients. Use of the T cell fraction in particular is very appealing potential biomarker that could be used in the adoption of repertoire sequencing for a greater range of clinical conditions, while still supplying the additional information repertoires bring. The data and code were also made available to the reviewers, and there's every indication that these will be suitably available upon publication. I am very happy recommending its publication, as I think it will be of use to the field. I have noted a small number of areas that caught my attention for the authors' consideration: several are places where some additional plotting may help reduce the possibility that the results are confounded, while the rest are minor typographical comments or similar.

1) I have a couple of queries about the T cell fraction. While none are major, given the importance of the metric to the study these are probably worth addressing:

a. L143 states that the number of unique CDR3b sequences is used in its calculation. Could the authors please clarify in the text to specify whether different recombinations (potentially using different V/J genes) producing the same CDR3s are grouped together in these calculations?

b. Given that the T cell fraction is calculated using the number of TCR rearrangements per sample, there's a chance that it might be confounded by differences in sequencing depth or errors, which are both in part dependent on the amount of input DNA. To rule these possibilities it might help the reader to include plots showing a lack of correlation between T cell fraction and number of productive TCR rearrangements, and between T cell fraction and amount of input gDNA. Additionally randomly selecting a matched number of sequences per donor prior to metric calculation can help rule out the possibility that these variables might be confounding (similar to how higher q values remove the effects of small clones in the later diversity metrics).

c. Re: Fig 1: while the range of interval length is given, the distribution is not. I wonder if perhaps a (potentially supplementary) plot of duration of interval vs T cell fraction would be instructive. This would also be a potentially independent useful validation of the use of 9.5 mo as a threshold, which it seems was determined by the authors in a significance screen by ROC analysis in their prior publication. As the

discussion remarks on the agreement between the results of this study broken by interval length and that previous publication, the authors may also wish to consider repeating that ROC analysis on these data, to see if a similar threshold is produced.
2) The Hill diversity/evenness data beautifully plotted in Fig 2 a/b is a robust way to illustrate these 'species'-level metrics.
a. However in my experience it can't be expected that the reader will know how to interpret these plots: even many people who study repertoire data are unfamiliar with these more ecological measures. I imagine this is perhaps even more true of the oncologists who may be interested in this study. I wonder if perhaps an explanatory sentence or two in the legend might help? I know there is some of this in the discussion, but that's likely going to be seen after the figures for many readers.
b. Also I think maybe the x axes would more accurately be labelled 'q values' (rather than 'Î±').
c. Some other values can be guessed at but aren't specifically defined in the legend, e.g. it only defines the median for Fig 2c and not the whisker/boxes, while the shaded confidence interval area in 2d is similarly not defined. It's also unclear where the T cell count information is coming from in this plot: were orthogonal T cell count data collected, or is this inferred from the repertoires?
3) The network analyses of Fig 3/4 are interesting, but currently presented in a way was harder to intrepret than surrounding sections, with several fairly field-specific analyses done with little explanation.
a. The text results of this analysis (beginning on L257. "The mean number of nodes detected was...") is abrupt, discussing results of an analysis that hasn't been established yet. This continued through the paragraph. E.g. it's unclear why the test for power law was performed: while it is well known that clonal repertoires tend to follow such distributions, the authors don't state why they think the network connectivities should.
b. Following on from my point (1) above, a common confounder in such connectivity networks is the possibility for sequencing and PCR errors to artificially inflate networks. The incidence of such errors could be biased towards certain samples, depending on factors like gDNA input and number of PCR cycles. You would also expect more abundant clonotypes on average to produce greater numbers of erroneous sequences, as a function of their occupancy of the reactions involved, which could contribute to the correlation seen in Fig 3a. Some control analyses or discussion might be useful, to help discount this possibility (with other plots in this figure lending weight to the notion that this is not artefactual).
c. Again there are some missing value definitions: 3a shaded area, box/whisker markers in e-g. The 3f y axis label is also mislabelled: 'MOI' should likely be 'MHI'.
4) It is also probably worth noting somewhere in the text that the connectivity difference between public/private sequences is exactly what one would expect given our understanding of biased V(D)J recombination (which underlies the production of public sequences in the first place).
https://doi.org/10.1073/pnas.0608907103 would be a classic citation for this, with more recently validations using higher-throughput TCRseq data discussed in https://doi.org/10.1111/imr.12665.
a. It has also similarly been observed that many T cells detectable in tumours are specific for viruses in both humans and mice, e.g. https://doi.org/10.1038/s41586-021-03704-y,
https://doi.org/10.1172/JCI150535, and https://doi.org/10.4049%2Fjimmunol.1601064.
5) As noted above, both the data and code are available to the reviewers, which is great. However there are a few things that could be changed to align more with the reproducibility and data reuse this journal

prides itself on.
a. The Github repo seems to have had a working directory uploaded to it, along with the associated detritus that such folders generate. There are several scripts and tables that seem to be derived from each other (eg X, X_v2), and at least one directory of what I suspect is analyses that didn't make it to the manuscript ('gliph2'). Tidying up these redundancies and not needed files would make the repo more navigable.
b. Similarly, while many of the scripts are sensible named, commenting is sparse and there's no summary or record of what metadata is recorded where and what scripts do what. While I'm sure the researchers running the analyses know exactly how to navigate this repo, anyone wishing to repeat or reapply these analyses will not, so an expanded README or similar would help a lot. This would be especially useful for the core pipeline of the study, and the key metadata values (e.g. Adaptive sample ID, patient IDs for those with >1 sample, treatment status/interval, ng DNA etc).
c. It looks like the journal's policies call for DOIs for code as well as data: I don't know about their provided Code Ocean option, but generating a Zenodo DOI for a GitHub release is very straightforward, and makes sense seeing as the code is already there.
6) A few of the axis tick label fonts are small relative to the rest of the figure (e.g. Fig 3a/c), so making these larger would help the short sighted readers like myself from having to frequently zoom in.
7) L202-204, sample exclusion. It's not entirely clear to me what "sequencing coverage <5" means here: fewer than five different TCRs, or counts, or something else? In any case it should be stated which of the three test arms (no-NACT/short/long) they fell from.
8) L183: the version/accession date of when McPAS was used should be stated. It might even be worth depositing the version of the database that was used somewhere accessible (e.g. the analysis repo), as since Nir Friedman's sad passing it appears that the resource's original URL is no longer working
9) Use of the word 'monoclonal' in L287-288 ("...a very large monoclonal network (Clonality=6.9) is visualized...") seems counter-intuitive, seeing as the sentence later points out how there are tens of thousands of different CDR3s are involved.
10) L254, possible typo or unclear abbreviation: "median og y"
11) Several times 'sequential' is used to mean 'relating to sequences'. I think most people will be able to figure out what this means, but (at least in my specific dialect of English) 'sequential' is mostly only used to describe things which occur in a sequence, rather than describe the sequences in the abstract themselves, so this threw me each time it came up. Changing to 'sequence' (e.g. L41/42, "TCR sequential convergence" could become "TCR sequence convergence") would avoid this if the authors wished.
- Jamie Heather

Methods

Are the methods appropriate to the aims of the study, are they well described, and are necessary controls included? Choose an item.

Conclusions

Are the conclusions adequately supported by the data shown? Choose an item.

Reporting Standards

Does the manuscript adhere to the journal's guidelines on minimum standards of reporting? Choose an item.

Choose an item.

Statistics

Are you able to assess all statistics in the manuscript, including the appropriateness of statistical tests used? Choose an item.

Quality of Written English

Please indicate the quality of language in the manuscript: Choose an item.

Declaration of Competing Interests

Please complete a declaration of competing interests, considering the following questions:

- Have you in the past five years received reimbursements, fees, funding, or salary from an organisation that may in any way gain or lose financially from the publication of this manuscript, either now or in the future?
- Do you hold any stocks or shares in an organisation that may in any way gain or lose financially from the publication of this manuscript, either now or in the future?
- Do you hold or are you currently applying for any patents relating to the content of the manuscript?
- Have you received reimbursements, fees, funding, or salary from an organization that holds or has applied for patents relating to the content of the manuscript?
- Do you have any other financial competing interests?
- Do you have any non-financial competing interests in relation to this paper?

If you can answer no to all of the above, write 'I declare that I have no competing interests' below. If your reply is yes to any, please give details below.

I declare that I have no competing interests

I agree to the open peer review policy of the journal. I understand that my name will be included on my report to the authors and, if the manuscript is accepted for publication, my named report including any attachments I upload will be posted on the website along with the authors' responses. I agree for my report to be made available under an Open Access Creative Commons CC-BY license (http://creativecommons.org/licenses/by/4.0/). I understand that any comments which I do not wish to be included in my named report can be included as confidential comments to the editors, which will not be published.

Choose an item.

To further support our reviewers, we have joined with Publons, where you can gain additional credit to further highlight your hard work (see: https://publons.com/journal/530/gigascience). On publication of this paper, your review will be automatically added to Publons, you can then choose whether or not to claim your Publons credit. I understand this statement.

Yes Choose an item.
