# Supplementary material for: T cell receptor repertoire sequencing reveals chemotherapy-driven clonal expansion in colorectal liver metastases
Source: Gigascience. 2023 May 10;12:giad032. doi: 10.1093/gigascience/giad032 (PMC10170408; doi:10.1093/gigascience/giad032)
Supplement: giad032_Reviewer_2_Report_Original_Submission — Satoshi Ueha -- 12/2/2022 Reviewed [file giad032_reviewer_2_report_original_submission.pdf]

Reviewer Report

Title: T cell receptor repertoire sequencing reveals chemotherapy-driven clonal expansion in colorectal liver metastases

Version: Original Submission **Date:** 12/2/2022

Reviewer name: Satoshi Ueha

Reviewer Comments to Author:

This manuscript by HÃ¸ye et al analyzed the effects of NACT on the TCR repertoire of TIL in CLM. Using several unique approaches of data analysis, they concluded that NACT increased the T cell infiltration and clonal expansion. Overall, data and conclusion seems to be convincing. The results that "shared clones have higher connectivity of TCR sequences" and "shared clones are enriched with antigen-specific TCRs registered in the database" were novel and interesting. On the other hand, as the authors point out in the discussion, there have been similar studies on other types of cancer, and the "increase in clonality with neoadjuvant chemotherapy" is not novel. It was difficult for me to understand the contents of the Results because the indicators used to evaluate the repertoire were not popular. I think it would be better to move some of the information on the significance of each indicator and the purpose of the analysis from the Discussion to the Results, and make the Results easier to read.

Major points

1. Line 247-248: clonality is evaluated using AUC of Hill diversity profile. However, since the "1-Pielou" index is commonly used in studies on TCR repertoire analysis, it would be better to show the calculation results of 1-Pielou index as a Fig.
2. line 238-241: Hill diversity profile was not explained, and it was difficult to understand. It would be helpful to include the information equivalent to Discussion line302-309.
3. Fig. 2 claims an "increase in Clonality with neoadjuvant chemotherapy". Although this study did not separate CD4+ T cells from CD8+ T cells in the TCR repertoire analysis, it has been reported that CD4+ T cells have higher Clonality than CD8+ T cells in their repertoires (Gueguen et al. 2021, Zhang et al. 2018). Therefore, the increased Clonality of tumor T-cell repertoires reported in this study may only reflect an increased proportion of CD8+ T cells in tumor-infiltrating T cells (Rudqvist et al. 2018). This point should be mentioned as a limitation in Discussion.
4. Line 257-259: I couldn't figure out why the authors performed network analysis just by reading the result. Please include something like Discussion line 359-363 in the result.
5. Line 278-279: Please provide a table with a list of target pathogens (flu, tuberculosis, etc.) of the clones that matched the McPAS database. Also, please indicate what type of cancer antigens (differentiation antigen, cancer testis antigen, neoantigen, etc.) are listed as "colorectal cancer".
6. Line 281-290: Fig. 4 only shows an example of TCR network, so I felt that the order of presentation should be switched with Fig. 3.
7. Line 340-343: "differences in clonality between NACT exposed and unexposed CLM might be of clinical relevance." I was not sure what this "clinical relevance" meant. Also, if the authors are trying to claim similarity of their tumor microenvironment based solely on the similarity between "differences in

TCR repertoire index between immune cold SCLC and immune hot NSCLC" and "differences in TCR repertoire index of CLM with and without NACT", it is an overstatement.
8. Line 352-355 compares the heterogeneity of TCR repertoires in tumors between NSCLC and SCLC, which seemed not relevant to the content of our paper.
Minor points
1. line 242: I do not understand what "increasing value of q" refers to. Is it referring to the "alpha" in Fig2 a, b?
2. In Fig. 2b, the plot for the treated group was lower than the plot for the untreated group, but I did not immediately understand the correspondence between this and the statement "Clonality was higher in the treated group". Legend stated that "Clonality was evaluated by 10-AUC", which I think should be clearly stated in the RESULT.
3. line 250-251: "regression analysis revealed a modest association between a high absolute number of T cells and clonality," I could not understand what this analysis was intended to claim.
4. Lines 259-260: I didn't understand what you were trying to argue with this analysis.
5. Line 262-264: The authors compared the clonality between repertoire that satisfy the power-law fit test and those that do not. Please provide a dot plot in addition to the average value.
6. Line 264-265: It is an overstatement to conclude that "clonally expanded datasets were not associated with power law degree distribution" because there are only a few repertoires that satisfy the power-law fit test.
7. Line 268-270: Fig3d might be a mistake for Fig3e. Also, Fig3e does not seem to be a graph comparing "mean connectivity fractions".
8. line 270: Fig3e" seems to be a mistake for "Fig3f".
9. Line 270-271: "in line with", but I did not understand how the discussion of public clone connectivity corresponds to the discussion of repertoire similarity between samples.
10. Spelling error: line 68 "Characterisation"
11. Fig3f, Y-axis seems to be MHI.

Methods

Are the methods appropriate to the aims of the study, are they well described, and are necessary controls included? Choose an item.

Conclusions

Are the conclusions adequately supported by the data shown? Choose an item.

Reporting Standards

Does the manuscript adhere to the journal's guidelines on [minimum standards of reporting?]() Choose an item.

Choose an item.

Statistics

Are you able to assess all statistics in the manuscript, including the appropriateness of statistical tests used? Choose an item.

Quality of Written English

Please indicate the quality of language in the manuscript: Choose an item.

Declaration of Competing Interests

Please complete a declaration of competing interests, considering the following questions:

- Have you in the past five years received reimbursements, fees, funding, or salary from an organisation that may in any way gain or lose financially from the publication of this manuscript, either now or in the future?
- Do you hold any stocks or shares in an organisation that may in any way gain or lose financially from the publication of this manuscript, either now or in the future?
- Do you hold or are you currently applying for any patents relating to the content of the manuscript?
- Have you received reimbursements, fees, funding, or salary from an organization that holds or has applied for patents relating to the content of the manuscript?
- Do you have any other financial competing interests?
- Do you have any non-financial competing interests in relation to this paper?

If you can answer no to all of the above, write 'I declare that I have no competing interests' below. If your reply is yes to any, please give details below.

I declare that I have no competing interests.

I agree to the open peer review policy of the journal. I understand that my name will be included on my report to the authors and, if the manuscript is accepted for publication, my named report including any attachments I upload will be posted on the website along with the authors' responses. I agree for my report to be made available under an Open Access Creative Commons CC-BY license (http://creativecommons.org/licenses/by/4.0/). I understand that any comments which I do not wish to be included in my named report can be included as confidential comments to the editors, which will not be published.

Choose an item.

To further support our reviewers, we have joined with Publons, where you can gain additional credit to further highlight your hard work (see: https://publons.com/journal/530/gigascience). On publication of this paper, your review will be automatically added to Publons, you can then choose whether or not to claim your Publons credit. I understand this statement.

Yes Choose an item.
