# Supplementary material for: T cell receptor repertoire sequencing reveals chemotherapy-driven clonal expansion in colorectal liver metastases
Source: Gigascience. 2023 May 10;12:giad032. doi: 10.1093/gigascience/giad032 (PMC10170408; doi:10.1093/gigascience/giad032)
Supplement: giad032_Reviewer_2_Report_Revision_1 — Satoshi Ueha -- 2/16/2023 Reviewed [file giad032_reviewer_2_report_revision_1.pdf]

Reviewer Report

Title: T cell receptor repertoire sequencing reveals chemotherapy-driven clonal expansion in colorectal liver metastases

Version: Revision 1 **Date:** 2/16/2023

Reviewer name: Satoshi Ueha

Reviewer Comments to Author:

The authors have mostly responded appropriately, and the revised version become more readable. It would be good to make additional revisions to the following points.
Response to
Major 3: It would be good to state more clearly in the discussion that "CD8 has higher clonality than CD4."
Major 5: Please provide a table or pie chart showing the composition of TCR target antigens (Flu X%, CMV Y%, etc.) that hit the McPAS database, rather than listing them as in Lines 287-289.
Major 7: Please describe the details of Clinical relevance with specific examples.
Regarding the L352-354, please clarify that this is a "working hypothesis" of the authors and that it is "CURRENTLY BEING TESTED".
Major 8: I could not understand the message of the statement until I read the reply "This is an important predictor for whether current immunotherapy approaches will have an effect, and may be part of the reason for the very good response in NSCLC compared to the poor response in SCLC and also in mCRC. effect, and may be part of the reason for the very good response in NSCLC compared to the poor response in SCLC and also in mCRC". How about including the equivalent of this in the main text?
Minor 5,6: I understood the meaning when I read the reply.
Is there a Figure corresponding to Line 266 "The connectivity fraction did not appear to be associated with the clonality of the network."?
I think the authors had better change the structure of the paragraph to make sure the following logic is conveyed.
In the BCR, the higher the clonality of a repatriate, the more the network tends to satisfy the power-low condition" (is this understanding correct?). On the other hand, in the TCR, the clonality was lower in the networks that met power-low. Thus, the authors concluded that "T cell repertoires follow different dynamics, and the results of this analysis was negative."

Methods

Are the methods appropriate to the aims of the study, are they well described, and are necessary controls included? Choose an item.

Conclusions

Are the conclusions adequately supported by the data shown? Choose an item.

Reporting Standards

Does the manuscript adhere to the journal's guidelines on [minimum standards of reporting?]() Choose an item.

Choose an item.

Statistics

Are you able to assess all statistics in the manuscript, including the appropriateness of statistical tests used? Choose an item.

Quality of Written English

Please indicate the quality of language in the manuscript: Choose an item.

Declaration of Competing Interests

Please complete a declaration of competing interests, considering the following questions:

- Have you in the past five years received reimbursements, fees, funding, or salary from an organisation that may in any way gain or lose financially from the publication of this manuscript, either now or in the future?
- Do you hold any stocks or shares in an organisation that may in any way gain or lose financially from the publication of this manuscript, either now or in the future?
- Do you hold or are you currently applying for any patents relating to the content of the manuscript?
- Have you received reimbursements, fees, funding, or salary from an organization that holds or has applied for patents relating to the content of the manuscript?
- Do you have any other financial competing interests?
- Do you have any non-financial competing interests in relation to this paper?

If you can answer no to all of the above, write 'I declare that I have no competing interests' below. If your reply is yes to any, please give details below.

I declare that I have no competing interests.

I agree to the open peer review policy of the journal. I understand that my name will be included on my report to the authors and, if the manuscript is accepted for publication, my named report including any attachments I upload will be posted on the website along with the authors' responses. I agree for my report to be made available under an Open Access Creative Commons CC-BY license (http://creativecommons.org/licenses/by/4.0/). I understand that any comments which I do not wish to be included in my named report can be included as confidential comments to the editors, which will not be published.

Choose an item.

To further support our reviewers, we have joined with Publons, where you can gain additional credit to further highlight your hard work (see: https://publons.com/journal/530/gigascience). On publication of this paper, your review will be automatically added to Publons, you can then choose whether or not to claim your Publons credit. I understand this statement.

Yes Choose an item.
